# Effectiveness of treatments for acute and subacute mechanical non-specific low back pain: a systematic review with network meta-analysis

**DOI:** 10.1136/bjsports-2020-103596

**Published:** 2021-04-13

**Authors:** Silvia Gianola, Silvia Bargeri, Gabriele Del Castillo, Davide Corbetta, Andrea Turolla, Anita Andreano, Lorenzo Moja, Greta Castellini

**Affiliations:** 1 Unit of Clinical Epidemiology, IRCCS Istituto Ortopedico Galeazzi, Milano, Lombardia, Italy; 2 Department of Biomedical Sciences for Health, University of Milan, Milano, Lombardia, Italy; 3 Physiotherapy Degree Course, Universita Vita-Salute San Raffaele, Milano, Lombardia, Italy; 4 Rehabilitation and Functional Recovery Department, IRCCS Ospedale San Raffaele, Milano, Lombardia, Italy; 5 Laboratory of Rehabilitation Technologies, IRCCS San Camillo Hospital, Venezia, Veneto, Italy; 6 Bicocca Bioinformatics Biostatistics and Bioimaging Centre - B4, School of Medicine and Surgery, University of Milan–Bicocca, Milano, Lombardia, Italy

**Keywords:** lower back, rehabilitation, pharmacology, disability, evidence based review

## Abstract

**Objective:**

To assess the effectiveness of interventions for acute and subacute non-specific low back pain (NS-LBP) based on pain and disability outcomes.

**Design:**

A systematic review of the literature with network meta-analysis.

**Data sources:**

Medline, Embase and CENTRAL databases were searched from inception until 17 October 2020.

**Eligibility criteria for selecting studies:**

Randomised clinical trials (RCTs) involving adults with NS-LBP who experienced pain for less than 6 weeks (acute) or between 6 and 12 weeks (subacute).

**Results:**

Forty-six RCTs (n=8765) were included; risk of bias was low in 9 trials (19.6%), unclear in 20 (43.5%), and high in 17 (36.9%). At immediate-term follow-up, for pain decrease, the most efficacious treatments against an inert therapy were: exercise (standardised mean difference (SMD) −1.40; 95% confidence interval (CI) −2.41 to –0.40), heat wrap (SMD −1.38; 95% CI −2.60 to –0.17), opioids (SMD −0.86; 95% CI −1.62 to –0.10), manual therapy (SMD −0.72; 95% CI −1.40 to –0.04) and non-steroidal anti-inflammatory drugs (NSAIDs) (SMD −0.53; 95% CI −0.97 to –0.09). Similar findings were confirmed for disability reduction in non-pharmacological and pharmacological networks, including muscle relaxants (SMD -0.24; 95% CI -0.43 to -0.04). Mild or moderate adverse events were reported in the opioids (65.7%), NSAIDs (54.3%) and steroids (46.9%) trial arms.

**Conclusion:**

With uncertainty of evidence, NS-LBP should be managed with non-pharmacological treatments which seem to mitigate pain and disability at immediate-term. Among pharmacological interventions, NSAIDs and muscle relaxants appear to offer the best harm–benefit balance.

## Background

Low back pain is a common symptom in people of all ages and socioeconomic status. The worldwide point prevalence of low back pain (acute, subacute and chronic) was 7.83% (95% CI 7.04 to 8.64) in 2017, with 577 million people affected at any one time.^1^ In 2017, low back pain was responsible for around 65 million years lived with disability, representing a deterioration of about 17.5% since 2007 mainly owing to population growth and ageing, with the greatest increase recorded for low-income and middle-income countries.^2^ People more often leave their job because of low back pain than diabetes, hypertension, neoplasm, asthma, heart and respiratory disease combined.^3^ About one in four adults in the USA had low back pain that lasted for at least 24 hours within the previous 3 months, with 7.6% adults reporting at least one episode of severe acute low back pain within a 1-year period.^4^ Moderate-to-severe pain and impairment of motor and psychological functions due to low back pain are the primary reasons for seeking medical consultation from a general practitioner.^5^

Despite its high prevalence, low back pain has a generally good prognosis. While a specific cause of low back pain can seldom be identified, the most prevalent type is mechanical, non-specific low back pain (NS-LBP).^6^ Most episodes of acute and subacute NS-LBP improve significantly within 6 weeks, and the average pain intensity is moderate (6 on a 100-point scale; 95% CI 3 to 10) by 12 months. However, two-thirds of people with low back pain still experience pain at 3 months (67%, 95% CI 50% to 83%) and at 12 months (65%, 95% CI 54% to 75%).^7^

Most guidelines agree on the first line of care in case of acute episode: advice, reassurance and encouragement to engage in light physical activity.^8^ When second-line treatment is needed, a range of therapeutic interventions (pharmacological and physiotherapy) for acute NS-LBP are available. The relative effects of various treatment options, when each option is compared against all others, are not well known. This uncertainty is reflected in the variety of recommendations in recent guidelines for acute NS-LBP.^8 9^ We explored the relative efficacy of currently available treatments for acute and subacute mechanical NS-LBP in terms of benefit and harm via a systematic review of the literature and network meta-analysis (NMA).

## Methods

### Protocol

The systematic review protocol was developed using guidance from the Preferred Reporting Items for Systematic Review and Meta-Analysis Protocols (PRISMA-P) statement,^10^ registered in the PROSPERO database (CRD42018102527, available at: http://www.crd.york.ac.uk/) and published.^11^ The methods have been described in the published protocol and are reported briefly here. We followed the PRISMA extension for NMA for reporting of the results.^12^ Additional sections specific to NMA are reported according to Chaimani *et al* 13 (see online supplemental A).

10.1136/bjsports-2020-103596.supp1 Supplementary data

### Eligibility criteria

Randomised controlled trials (RCTs) had to involve both adult men and women who had experienced pain for up to 12 weeks due to acute or subacute NS-LBP.^14^ Non-pharmacological treatments (eg, manual therapy) including acupuncture and dry needling or pharmacological treatments for improving pain and/or reducing disability considering any delivery parameters were included. The comparator was an inert treatment encompassing sham/placebo treatment or no treatment.

### Outcomes

The primary outcomes were pain intensity and disability. The secondary outcomes were any occurrence of adverse events (eg, number of events, number of participants who experienced an event). Follow-up was classified as immediate-term (closest to 1 week), short-term (closest to 1-month assessment), medium-term (closest to 3–6 months) and long-term effects (closest to 12 months).

### Data sources

We searched the following electronic databases since the inception date up to 27 February 2019 and updated on 17 October 2020: Medline (PubMed), CENTRAL and Embase (Elsevier, EMBASE.com) using the appropriate Thesaurus and free-text terms (see the study protocol for the search strategy).^11^ Additional studies were identified by scanning the reference lists of relevant reviews and contacting the study authors. No restriction on language or publication period was applied. Studies published in a language other than English for which no translation could be obtained were classified as potentially eligible but were not entered in the final review.

### Study selection

We tested the eligibility criteria by piloting a small sample (10 trials). Two independent reviewers screened the title and the abstract of the publications retrieved by the search strategy and assessed the full text for potential inclusion. Studies not meeting the inclusion criteria were discarded. Disagreements between reviewers were resolved by discussion and consultation with a third reviewer, if necessary. Covidence software^15^ was used to manage this phase.

### Data extraction

We designed and piloted a data collection form created with Excel (Microsoft). Two reviewers independently extracted the study characteristics and outcome data. Disagreements were resolved through discussion or with assistance from a third reviewer, if necessary. From each study we extracted: name of first author, year of publication, setting, number of centres and population definition (acute/subacute), number, sex and age of participants, type of intervention and its duration, primary and secondary study outcomes data at interested time point of follow-up.

All relevant arm-level final value scores were extracted. When these were lacking, the final value data were derived from the difference between the baseline and the mean change values. The SDs were imputed (eg, using the average of the available SD for the same instrument or baseline SD for the same intervention within study when different instruments are used).^16^ Not enough information was present to perform a secondary analysis using mean change values.

When per-protocol and intention-to-treat analyses were reported, we prioritised intention-to-treat data as the effect of assignment to intervention might be more appropriate to inform stakeholder about effects of interventions in a healthcare perspective.^17^ When population had a duration of pain exceeding for a few weeks over the definition of subacute NS-LBP and when the outcomes of interest were missing, we contacted the corresponding study authors to obtain data.

### Risk of bias (RoB) within individual studies

Two reviewers independently assessed the RoB of the included trials. We assessed the RoB for each study using the following RoB assessment tools recommended by the Cochrane Collaboration^16^: random sequence generation, allocation concealment, blinding of participants, providers and outcome assessment, incomplete outcome data (dropouts) and selective outcome reporting. In the selective outcome data, we accounted for a broader assessment considering also the selective non-reporting RoB due to missing results in index meta-analyses (eg, missing or unavailable outcome results crosschecked from method plans) according to published criteria by Page *et al*.^18–20^ For each study, the items were scored as high, low or unclear (not enough information reported) RoB.^16^

In order to obtain an overall RoB assessment,^21^ the certainty of evidence of the Grading of Recommendation Assessment, Development and Evaluation (GRADE) approach, allocation concealment, blinding of outcome assessment and incomplete outcome data were all carefully examined to classify each study as: low RoB when all three criteria are met; high risk when at least one criterion was not met and moderate in the remaining cases. Since allocation concealment, blinding of outcome assessment and incomplete outcome data were not expected to vary in importance across the primary outcomes, we summarised the RoB of each study. Disagreements were resolved through discussion or arbitration with a third review author.

### Small study effects

Small study effects were assessed for each outcome (when >10 RCTs were available) using the *netfunnel* command in Stata 15^22^ generating a comparison-adjusted funnel plot for a network of interventions. In the absence of small study effects, the comparison-adjusted funnel plot should be symmetric around the zero line.

### Certainty of evidence

We assessed the certainty of evidence contributing to the network estimate of the main outcomes by means of the GRADE framework. The five GRADE domains were applied: study limitations, indirectness, inconsistency (heterogeneity and incoherence), imprecision and publication bias by Confidence in Network Meta-Analysis (CINeMA), a web application that simplifies evaluation of confidence in the findings from an NMA.^23^ The framework combines judgments about direct evidence with their statistical contribution to NMA results, enabling evaluation of the credibility of NMA treatment effects. Online supplemental P and Q include the operational criteria used to form judgements for each domain.

### Data synthesis and analysis

#### Pairwise comparisons

Conventional pairwise meta-analysis for each outcome was performed using a random effects model for each treatment comparison with at least two studies.^24^

#### Summary of the network

For the network analysis, according to the PRISMA-NMAs,^12^ the eligible interventions are reported in the study protocol^11^ and the process leading to node grouping and nodes adopted is described in online supplemental D, box 1 and box 2, respectively.^25^

#### Assumption of transitivity

To ensure transitivity and enough statistical power for robust conclusions, a sufficient number of trials and treatment comparisons with sufficient data were evaluated. Judgement of treatments’ network connection was presented and evaluated graphically by network plot.

Transitivity is the assumption that the distributions of effect modifiers (covariates associated with intervention effects) are balanced across comparisons in the network in order to allow the estimation effects for indirect comparisons.^26 27^ To our knowledge, no robust effect modifiers are established in NS-LBP trials,^28^ thus we supposed the following potential effect modifiers based on clinical and methodological experience: stage of low back pain, presence of leg pain or sciatica, mean age, percentage of male participants, baseline severity, length of treatment, number of randomised subjects and psychological assessment. Judgement of transitivity was based on visualisation of tables and box plots of these variables by trials, by interventions and by head-to-head comparisons (online supplemental E) in order to assess any dissimilarity between comparisons in the network that could threaten the assumption of transitivity. We assessed the insufficient reporting of effect modifiers and the pairwise comparisons containing few studies as limitation of the transitivity assessment.^29^ In fact, outlier treatment comparisons (ie, insufficiently study’s characteristics reported) were carefully appraised. Non-eligible treatment arms (eg, bed rest advice) or non-eligible comparisons (eg, head-to-head comparison of the same intervention) were not considered.^30^

#### Network meta-analysis

After checking the shared nodes in the compared interventions and covariates for any effect modifiers, we assumed that people with NS-LBP meeting the inclusion criteria were, in principle, equally likely to be randomised to any of the eligible NS-LBP interventions.

Random effects NMA within frequentist setting was conducted for connected networks.^26 31–33^ We presented the interval plot results for each intervention compared with reference standard (inert treatment) and the league table for estimates of all interventions against all by outcomes. Then, in order to identify the superiority of the interventions, we estimated the probability of being the best, the mean rank and the surface under cumulative ranking (SUCRA) which expresses the percentage of effectiveness or safety of a treatment that can be ranked first without uncertainty.^34^ We estimated all cumulative ranking probabilities (line plots of the cumulative probabilities vs ranks) for each treatment and outcome^35^ setting up to 8780 draws and 50 000 replicates. All analyses were performed using Stata V.15 with *mvmeta* command and network graphs package.^22 32 36 37^

Results were summarised using the standardised mean differences (SMDs) when different outcome measurements were reported for each trial. The uncertainty of all estimates is expressed with their 95% CI. Details on the analyses are provided in the published protocol.^11^ Difference in the methods between the protocol and the present review are reported in online supplemental B.

#### Assessment of network inconsistency (heterogeneity and incoherence)

Variation in treatment effects between studies (ie, heterogeneity) and variation between direct and indirect sources of evidence (ie, incoherence) are two concepts related to the inconsistency.^27 29^

The assessment of statistical heterogeneity in the entire network was based on the magnitude of the heterogeneity variance parameter (τ2) estimated by using NMA models.^38^ We assumed equal heterogeneity across all treatment comparisons accounting for correlations induced by multiarm studies.^39 40^ Then, to assess presence of global inconsistency, we used a full design-by-treatment interaction random effects model (global χ^2^ test). If the null hypothesis of inconsistency parameters being equal to zero was not rejected, we fit a consistency model. We presented local inconsistency estimates using forest plots and side-splitting for direct and indirect estimates in each available comparison (online supplemental I and J). When global significant inconsistency was found,^26 32 33^ multiple strategies were explored.^33^ We first checked the dataset for data extraction errors or outlier effect sizes among comparisons (visually inspected by pairwise meta-analysis). Then, we tried to interpret the significant inconsistency parameters separating indirect from direct evidence (side-splitting) and finally we explored the observed inconsistency using prespecified covariates in network meta-regression analyses and subgroup analyses. If any strategy explained the inconsistency, we presented only forest plots grouped into direct and indirect estimates (network forests).^33^

#### Meta-regression and subgroup analyses

We performed network meta-regression random effects within a frequentist framework with *metareg* command in Stata using aggregate-level data to examine relationship between treatments effects and each specified covariate (age, percentage of male, stage of low back pain, baseline severity of pain, presence of leg pain or sciatica, RoB).^41^

When inconsistency remains unexplained by meta-regression, we explored the treatments effects performing subgroup analyses into pharmacological and non-pharmacological interventions groups.^42^

## Results

### Study selection

**Figure 1:**
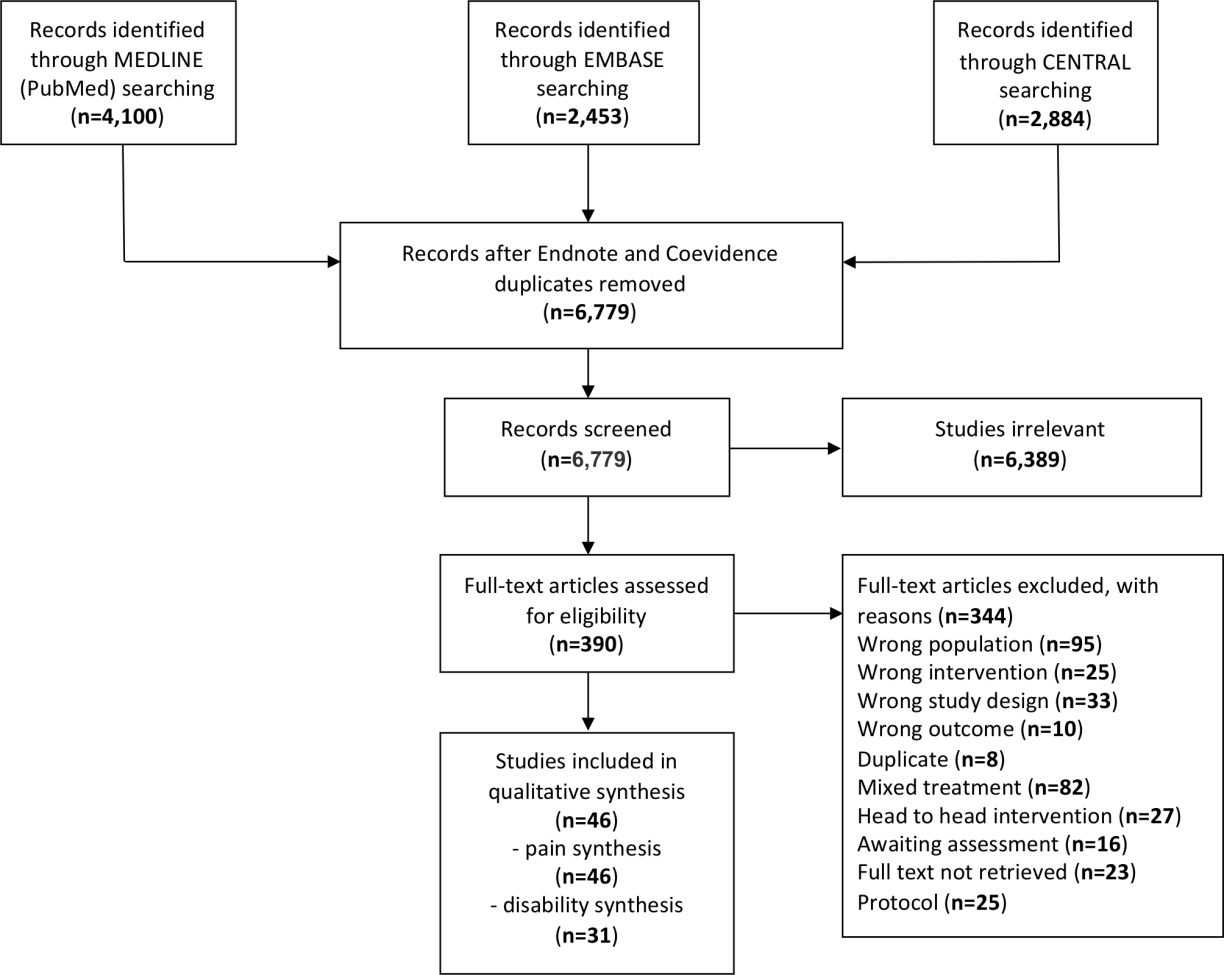
Flow chart of study selection.

After removal of duplicates, 6779 records were retrieved and 6389 records were discarded. The full text of the remaining 390 records was examined and 344 did not meet the inclusion criteria: 95 involved a different study population (eg, chronic pain), 82 had mixed treatments (eg, manual therapy plus usual care), 25 described interventions not pertinent to the present study (eg, bed rest), 27 were head-to-head interventions (eg, exercise vs exercise), 10 reported outcomes not pertinent to the present study (eg, cost-effectiveness related to pain), 33 had a study design other than RCT, 8 were further duplicates, 25 were protocols, 16 were awaiting assessment for language (original not in English or Italian) and in 23 instances the full text could not be retrieved. In total, 18 authors were contacted; four of the eight who responded provided useful data for our analysis. Finally, 46 studies were included (citations in References in online supplemental C). The study flow diagram is illustrated in figure 1.

### Study and participant characteristics

A total of 8765 participants were included in 46 trials. The sample size of trials ranged between 21.5 and 91.3 participants (IQR) with a median of 39.5 participants each. Most studies involved people with acute NS-LBP (n=30 trials). Overall, 22 were multicentre and 24 were single-centre trials. The median year of publication of RCTs was 2003 (IQR 1995–2013). The median age of participants was 40.4 years old (IQR: 37–43) and the median percentage of males was 52% (IQR 43.7%–60%)

Table 1 presents the general characteristics of the studies and the participants. No important concerns were raised regarding the violation of the transitivity assumption when the potential effect modifiers were evaluated. Studies and participants characteristics stratified by trials, by interventions and by head-to-head comparisons are summarised in online supplemental E. The inconsistency assessment is reported globally and locally in online supplemental J, table 1 and table 2, respectively.

**Table 1:** General characteristics

Study characteristic	No. (%) of RCTs (N=46)
Year of publication	
1961–1970	1 (2.2)
1971–1980	2 (4.3)
1981–1990	7 (15.2)
1991–2000	8 (17.4)
2001–2010	16 (34.8)
2011–2019	12 (26.1)
Intervention*	
Acupuncture	2 (1.7)
Back school	2 (1.7)
Cognitive behavioural therapy	4 (3.3)
Education	5 (4.2)
Exercise	7 (5.8)
Heat wrap	5 (4.2)
Inert treatment	34 (28.3)
Manual therapy	12 (10.0)
Muscle relaxant	10 (8.3)
NSAIDs	18 (15.0)
Opioids	3 (2.5)
Paracetamol	5 (4.2)
Physical therapy	1 (0.8)
Steroids	3 (2.5)
Usual care	9 (7.5)
Length of treatment*	
≤7 days	66 (55)
>7 days	29 (24.2)
Not reported	25 (20.8)
Stage of NS-LBP	
Acute NS-LBP	30 (65.2)
Subacute NS-LBP	2 (4.4)
Acute and subacute	14 (30.4)
Presence of leg pain or sciatica**†**	
Yes	15 (31.2)
No	19 (39.6)
Not stated	14 (29.2)
Study setting	
Multicentre	22 (47.8)
Single centre	24 (52.2)
Outcomes and follow-up	
Pain (n=46)	
At immediate-term (1 week)	35 (76.1)
At short-term (1 month)	16 (34.8)
At medium-term (3–6 months)	13 (28.3)
At long-term (12 months)	9 (19.6)
Disability (n=31)	
At immediate-term (1 week)	21 (67.7)
At short-term (1 month)	14 (45.2)
At medium-term (3–6 months)	11 (35.5)
At longterm (12 months)	7 (22.6)
Any adverse event	26 (56.5)

*The total number of interventions is higher due to multiarms trials (n=120).

†One study involved three patient subgroups (one with leg pain, two without leg pain) (n=48).

NSAIDs, non-steroidal anti-inflammatory drugs; NS-LBP, non-specific low back pain.

### RoB assessment

O nline supplemental F, table 1 and figure 1 summarise the RoB assessments. Of the 46 studies, 9 (19.6%) had low RoB, 20 (43.5%) unclear RoB and 17 (36.9%) high RoB.

### Pain

**Figure 2:**
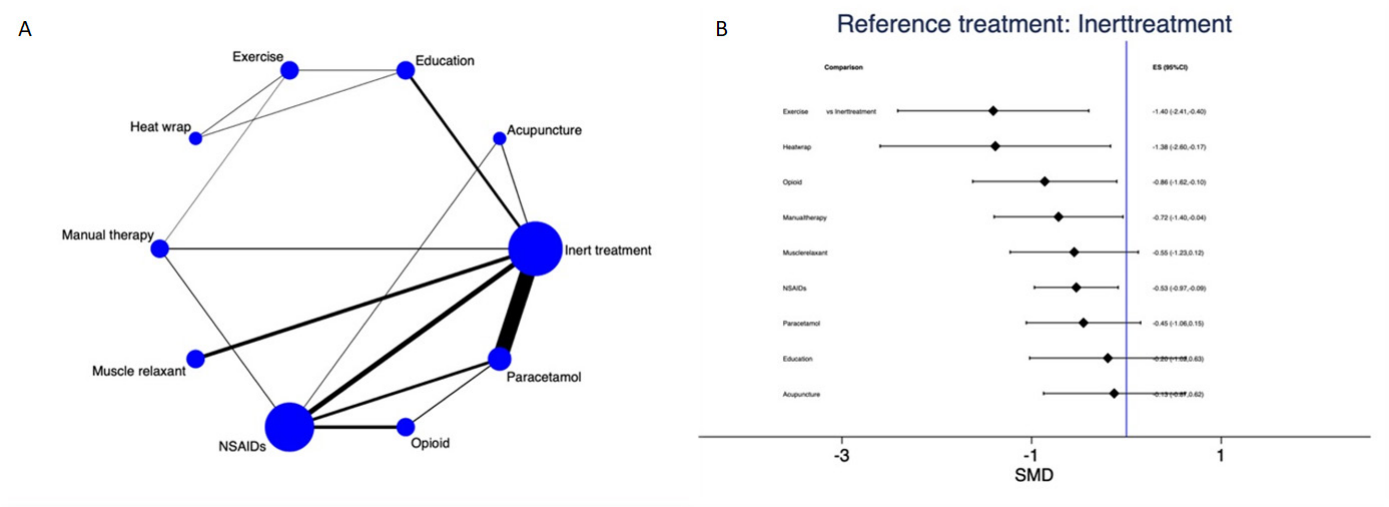
Pain at immediate-term (1 week): network plot (A) and interval plot (B). NSAIDs, non-steroidal anti-inflammatory drugs; SMD, standardised mean difference.

Pain was assessed in 35 studies at immediate-term (1 week) of follow-up, in 16 studies at 1 month, in 13 studies at 3–6 months and in 9 studies at 12 months. No evidence of publication bias was present (online supplemental N). Under consistency (p value=0.52), the NMA of pain at 1 week (16/35 studies involving 2905 subjects with data provided for 15 direct comparisons between 10 different treatment nodes, figure 2A) showed that exercise (SMD −1.40; 95% CI −2.41 to –0.40), heat wrap (SMD −1.38; 95% CI −2.60 to –0.17), opioids (SMD −0.86; 95% CI −1.62 to –0.10), manual therapy (SMD −0.72; 95% CI −1.40 to –0.04) and non-steroidal anti-inflammatory drugs (NSAIDs) (SMD −0.53; 95% CI −0.97 to –0.09) significantly reduced pain compared with inert treatment (figure 2B). The contribution matrix of direct and indirect evidence is depicted in online supplemental O, figure 1A. Pairwise meta-analyses and forest plot of NMA data are presented in online supplemental H, table 1 and online supplemental I, figure 1, respectively. Table 2 presents NMA estimates of all interventions against all. The ranking of treatments based on cumulative probability plots and SUCRAs is presented in online supplemental M, figure 1 and table 2. The most effective treatment was exercise (89.2%) and the least effective was inert treatment (10.7%).

**Table 2:** League table presenting all network meta-analysis estimates of pain outcome at immediate-term (1 week)

Inert treatment									
0.13 (−0.62, 0.87)	Acupuncture								
0.20 (−0.63, 1.02)	0.07 (−1.04, 1.17)	Education							
**1.40** (**0.40, 2.41**)	**1.28** (**0.04, 2.51**)	**1.21** (**0.31, 2.11**)	Exercise						
**1.38** (**0.17, 2.60**)	1.25 (−0.16, 2.67)	**1.19** (**0.17, 2.20**)	−0.02 (−1.03, 0.99)	Heat wrap					
**0.72** (**0.04, 1.40**)	0.59 (−0.38, 1.55)	0.52 (−0.44, 1.48)	−0.69 (−1.67, 0.29)	−0.67 (−1.92, 0.58)	Manual therapy				
0.55 (−0.12, 1.23)	0.42 (−0.58, 1.43)	0.36 (−0.71, 1.42)	−0.85 (−2.07, 0.36)	−0.83 (−2.22, 0.56)	−0.17 (−1.12, 0.79)	Muscle relaxant			
**0.53** (**0.09, 0.97**)	0.40 (−0.35, 1.15)	0.33 (−0.58, 1.25)	−0.88 (−1.93, 0.18)	−0.85 (−2.12, 0.41)	−0.19 (−0.89, 0.51)	−0.02 (−0.83, 0.78)	NSAIDs		
**0.86** (**0.10, 1.62**)	0.73 (−0.25, 1.71)	0.67 (−0.44, 1.77)	−0.54 (−1.77, 0.68)	−0.52 (−1.93, 0.89)	0.15 (−0.80, 1.09)	0.31 (−0.71, 1.33)	0.33 (−0.32, 0.98)	Opioids	
0.45 (−0.15, 1.06)	0.32 (−0.57, 1.22)	0.26 (−0.75, 1.26)	−0.95 (−2.10, 0.19)	−0.93 (−2.27, 0.41)	−0.26 (−1.11, 0.58)	−0.10 (−1.00, 0.81)	−0.08 (−0.63, 0.48)	−0.41 (−1.15, 0.34)	Paracetamol

Interventions are reported in alphabetical order from left to right except for reference treatment (inert treatment). The estimate is in the cell where the column-defining treatment and the row-defining treatment intersect. For efficacy SMD>0 favours the row-defining treatment whereas SMD<0 favours the column-defining treatment. Significant results are given in bold.

NSAIDs, non-steroidal anti-inflammatory drugs; SMD, standardised mean difference.

Under consistency (p value=0.36), the NMA of pain at short-term (1 month) (11/16 studies involving 2378 subjects with data provided for 10 direct comparisons between nine different treatment nodes, online supplemental G, figure 1A) showed that manual therapy (SMD −0.83; 95%CI −1.44 to –0.22) significantly reduced pain compared with inert treatment (online supplemental L, figure 1A). The contribution matrix of direct and indirect evidence is presented in online supplemental O, figure 1B. Pairwise meta-analyses and forest plot of NMA data are presented in online supplemental H, table 2 and online supplemental I, figure 2, respectively. Online supplemental M, table 1A presents NMA estimates of all interventions against all. The ranking of treatments based on cumulative probability plots and SUCRAs is presented in online supplemental M, figure 2 and table 2. The most effective treatment was manual therapy (91.1%) and the least effective was education (4.9%).

The NMA of pain at medium-term (3–6 months) (11/13 studies involving 2458 subjects with data provided for 10 different treatment nodes, online supplemental G, figure 1B) showed a disconnected network. Pairwise meta-analyses are presented in online supplemental H, table 3: manual therapy was superior to inert treatment in reducing pain at 3–6 months.

**Figure 3:**
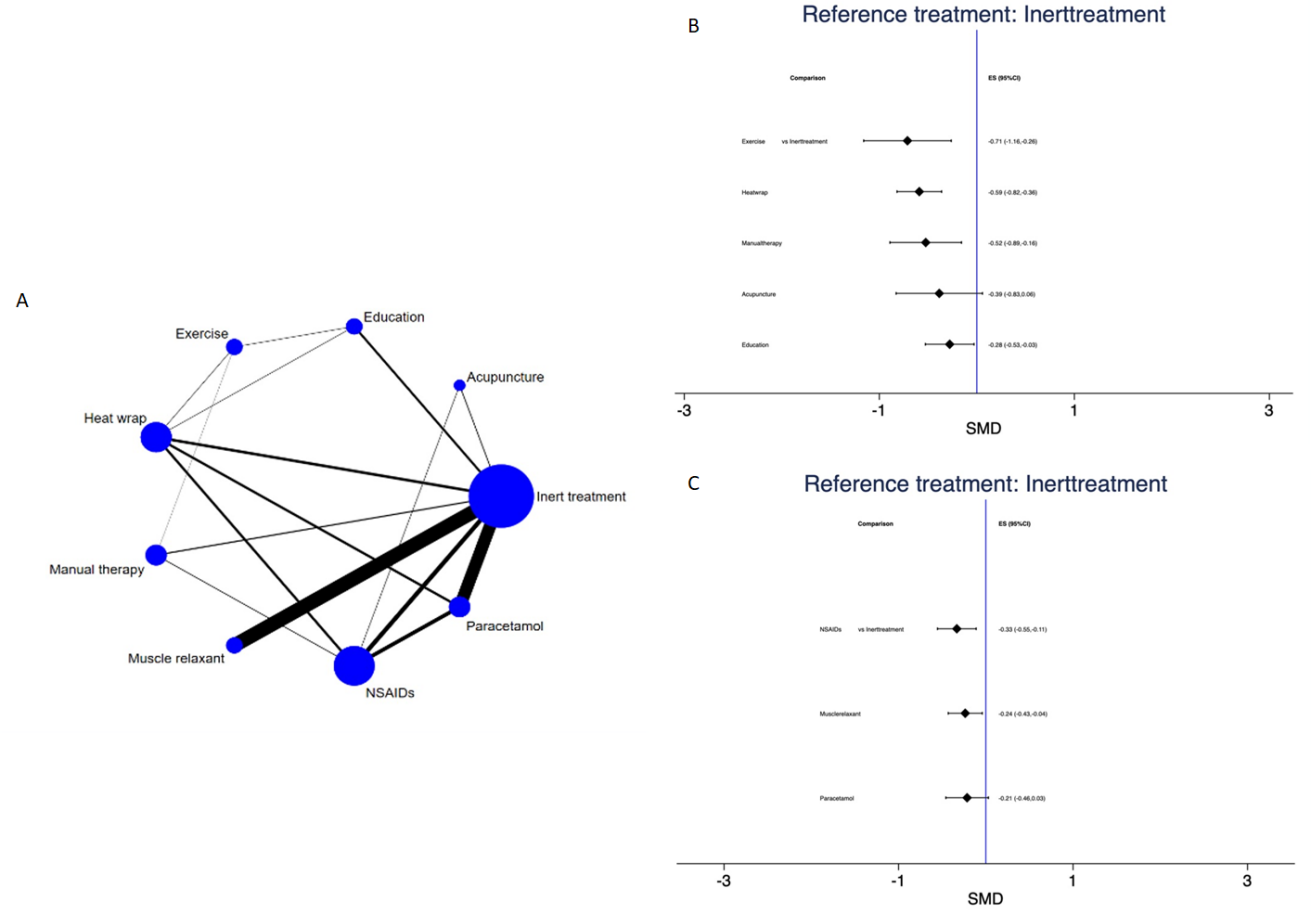
Disability at immediate-term (1 week): network plot (A) and interval plot (B) for non-pharmacological interventions and (C) for pharmacological interventions. NSAIDs, non-steroidal anti-inflammatory drugs; SMD, standardised mean difference.

Under consistency (p value=1), the NMA of pain at long-term (12 months) (5/9 studies involving 938 subjects with data for four direct comparisons between five different treatment nodes, online supplemental G, figure 1C) showed no statistically significant intervention against inert treatment (online supplemental L, figure 1B). The contribution matrix of direct and indirect evidence is presented in online supplemental O, figure 1C. Pairwise meta-analyses and forest plot of NMA data are presented in online supplemental H, table 4 and online supplemental I, figure 3, respectively. Online supplemental M, table 1B presents NMA estimates of all interventions against all. The ranking of treatments based on cumulative probability plots and SUCRAs is presented in online supplemental M, figure 3 and table 2. The most effective treatment was cognitive behavioural therapy (CBT) (73.7%) and the least effective was inert treatment (15.3%).

### Disability

Disability was assessed in 21 studies at 1 week of follow-up, in 14 studies at 1 month, in 11 studies at 3–6 months and in 7 studies at 12 months. No evidence of publication bias was present (online supplemental N).

The NMA of disability at immediate-term (1 week) (15/21 studies involving 4167 subjects with data provided for 16 direct comparisons between nine different treatment nodes, figure 3A) showed sources of inconsistency (p value=0.001). Pairwise meta-analyses and forest plot of NMA data are presented in online supplemental H, table 5 and online supplemental I, figure 4, respectively. Strategies to explore inconsistency are reported in online supplemental J for meta-regression and in online supplemental K for subgroup analysis. Inconsistency was explained by subgroup analysis. In the non-pharmacological group, exercise (SMD −0.71; 95% CI −1.16 to –0.26), heat wrap (SMD −0.59; 95% CI −0.82 to –0.36), manual therapy (SMD −0.52; 95% CI −0.89 to –0.16) and education (SMD −0.28; 95% CI −0.53 to –0.03) were statistically significant compared with inert treatment (figure 3B). Online supplemental K, table 1A presents NMA estimates of all interventions against all. The ranking of treatments based on cumulative probability plots and SUCRAs showed that the most effective treatment was manual therapy (80.3%) and the least effective was inert treatment (2.9%) (online supplemental K, table 2A). In the pharmacological group, NSAIDs (SMD −0.33; 95% CI −0.55 to -0.11) and muscle relaxants (SMD −0.24; 95% CI −0.43 to -0.04) were statistically significant compared with inert treatment (figure 3C). Online supplemental K, table 1B presents NMA estimates of all interventions against all. The ranking of treatments based on cumulative probability plots and SUCRAs showed that the most effective treatment was NSAIDs (94.6%) and the least effective was inert treatment (7.9%) (online supplemental K, table 2B).

The NMA of disability at short-term (1 month) (11/14 studies involving 2463 subjects with data provided for 13 direct comparisons between 10 different treatment nodes, online supplemental G, figure 2A) showed sources of inconsistency (p value=0.0107). Pairwise meta-analyses and forest plot of NMA data are presented in online supplemental H, table 6 and online supplemental I, figure 5, respectively. Manual therapy was statistically significant compared with education and exercise and a positive trend was found in favour of low-dose steroids compared to NSAIDs.

Strategies to explore inconsistency are reported in online supplemental J for meta-regression and in online supplemental K for subgroup analysis. Inconsistency was not explained by any strategy.

The NMA of disability at medium-term (3–6 months) (9/11 studies involving 1404 subjects with data provided for nine different treatment nodes, online supplemental G, figure 2B) was disconnected; pairwise meta-analyses are presented in online supplemental H, table 7: low-dose steroids were statistically significant compared to NSAIDs as well as manual therapy compared to education and exercise.

Under consistency (p value=0.77), the NMA of disability at long-term (12 months) (6/7 studies involving 1031 subjects with data provided for five intervention nodes, online supplemental G, figure 2C) showed that no intervention was statistically significant against inert treatment (online supplemental L, figure 2A). The contribution matrix of direct and indirect evidence is presented in online supplemental O, figure 2A. Pairwise meta-analyses and forest plot of NMA data are presented in online supplemental H, table 8 and online supplemental I, figure 6, respectively. Online supplemental M, table 3A presents NMA estimates of all interventions against all. The ranking of treatments based on cumulative probability plots and SUCRAs is presented in online supplemental M, figure 4 and table 4. The most effective treatment was CBT (68.5%) and the least effective was inert treatment (22.7%).

### Adverse events

Twenty-six studies (56.5%) reported adverse events. No events were reported for acupuncture, education, exercise or manual therapy. Mild-moderate events occurred with the use of heat wrap, muscle relaxants, NSAIDs, opioids, paracetamol, steroids and inert treatment. No study reported treatment-related disabling events or death and only one reported three severe adverse events (one in the NSAIDs arm and two in the inert treatment arm). Mild or moderate adverse events occurred most often in the opioids (65.7%), the NSAIDs (54.3%) and the steroids arm (46.9%). But because adverse events reporting was heterogeneous for number of people with NS-LBP and number of events, we cannot quantitate these data (table 3).

**Table 3:** Adverse events reported as number of people with NS-LBP experiencing adverse events and number of events classified from grade 1–5

Study (Author, year)	Category of intervention	Adverse events						
		n	%	AE 1 (mild), n	AE 2 (moderate), n	AE 3 (severe), n	AE 4 (disabling), n	AE 5 (death), n
Shin, 2013	Acupuncture	0	0	–	–	–	–	–
Mayer, 2005	Education	0	0	0	0	0	0	0
Traeger, 2019	Education	0	0	–	–	–	–	–
Mayer, 2005	Exercise	0	0	0	0	0	0	0
Mayer, 2005	Heat wrap	0	0	0	0	0	0	0
Nadler, 2002	Heat wrap	–	6.2	–	–	–	–	–
Nadler, 2003b	Heat wrap	–	15	–	–	–	–	–
Nadler, 2003a	Heat wrap	1	1.1	–	–	–	–	–
Santilli, 2006	Manual therapy	0	0	–	–	–	–	–
Takamoto, 2015	Manual therapy	0	0	–	–	–	–	–
Takamoto, 2015	Manual therapy	0	0	–	–	–	–	–
von Heymann, 2013	Manual therapy	0	0	–	–	–	–	–
Berry, 1988	Muscle relaxant	25	42,4	–	–	–	–	–
Hindle, 1972	Muscle relaxant	0	0	0	0	0	0	0
Ketenci, 2005	Muscle relaxant	–	18; 10; 5; 5*	–	–	0	0	0
Ketenci, 2005	Muscle relaxant	–	28; 3; 15*	–	–	0	0	0
Ralph, 2008	Muscle relaxant	–	–	74	–	–	–	–
Serfer, 2009	Muscle relaxant	–	–	69	–	–	–	–
Serfer, 2009	Muscle relaxant	–	–	85	–	–	–	–
Tuzun, 2003	Muscle relaxant	–	–	4	–	–	–	–
Amlie, 1987	NSAIDs	18	13	14	6	1	–	–
Dreiser, 2003	NSAIDs	15	12.1†	–	–	0	0	0
Dreiser, 2003	NSAIDs	17	13.9†	–	–	0	0	0
Eken, 2014	NSAIDs	4	8.7	4	–	–	–	–
Goldie, 1968	NSAIDs	8	32	–	–	–	–	–
Miki, 2018	NSAIDs	5	7.9†	–	–	–	–	–
Nadler, 2002	NSAIDs	–	10.4	–	–	–	–	–
Nadler, 2003b	NSAIDs	–	25	–	–	–	–	–
Nadler, 2003a	NSAIDs	0	0	–	–	–	–	–
Sae-Jung, 2016	NSAIDs	4	12	–	–	–	–	–
Shin, 2013	NSAIDs	0	0	–	–	–	–	–
Szpalski, 1994	NSAIDs	1	2.7	–	–	–	–	–
Veenema, 2000	NSAIDs	–	–	8	–	–	–	–
Videman, 1984	NSAIDs	19	54.3	–	–	–	–	–
von Heymann, 2013	NSAIDs	0	0	–	–	–	–	–
Eken, 2014	Opioid	7	15.5	6	1	–	–	–
Veenema, 2000	Opioid	–	–	41	–	–	–	–
Videman, 1984	Opioid	23	65.7	–	–	–	–	–
Eken, 2014	Paracetamol	4	8.7	4	–	–	–	–
Miki, 2018	Paracetamol	1	1.6†	–	–	–	–	–
Nadler, 2002	Paracetamol	–	4.4	–	–	–	–	–
Williams, 2014	Paracetamol	99	18.0†	–	–	–	–	–
Williams, 2014	Paracetamol	99	18.0†	–	–	–	–	–
Eskin, 2014	Steroids	–	–	0	0	0	0	0
Sae-Jung, 2016	Steroids	15	46.9†	–	–	–	–	–
Amlie, 1987	Inert treatment	24	17	19	8	2	–	–
Berry, 1988	Inert treatment	12	22.6	–	–	–	–	–
Dreiser, 2003	Inert treatment	25	19.8†	–	–	0	0	0
Eskin, 2014	Inert treatment	–	–	0	0	0	0	0
Goldie, 1968	Inert treatment	5	20	–	–	–	–	–
Hindle, 1972	Inert treatment	0	0	0	0	0	0	0
Ketenci, 2005	Inert treatment	–	22; 4*	–	–	0	0	0
Nadler, 2003b	Inert treatment	–	12	–	–	–	–	–
Nadler, 2003a	Inert treatment	0	0	–	–	–	–	–
Nadler, 2003a	Inert treatment	0	0	–	–	–	–	–
Ralph, 2008	Inert treatment	–	–	26	–	–	–	–
Santilli, 2006	Inert treatment	0	0	–	–	–	–	–
Serfer, 2009	Inert treatment	–	–	34	–	–	–	–
Szpalski, 1994	Inert treatment	–		–	–	–	–	–
Takamoto, 2015	Inert treatment	0	0	–	–	–	–	–
Traeger, 2019	Inert treatment	0	0	–	–	–	–	–
Tuzun, 2003	Inert treatment	–	–	4	–	–	–	–
von Heymann, 2013	Inert treatment	0	0	–	–	–	–	–
Williams, 2014	Inert treatment	98	18.0	–	–	–	–	–

All references of included studies are provided in online supplemental C.

*Percentage of people with NS-LBP reporting specific adverse events (eg, headache, diarrhea, dyspepsia).

†Percentages were reported slightly different in the primary studies (unclear about randomised people with NS-LBP).

NS-LBP, non-specific low back pain.

### Grading of evidence

We incorporated the GRADE judgments in online supplemental P and Q. The certainty of evidence for the treatment effects of efficacy varied.

## Discussion

To our knowledge, this is the largest NMA to date in the field of low back pain (46 RCTs involving 8765 participants assigned to pharmacological, non-pharmacological or inert treatment). We found that pharmacological and non-pharmacological interventions were more efficacious than inert treatment for reducing pain intensity and disability due to acute and subacute mechanical NS-LBP. Overall, the certainty of evidence ranged from very low to moderate, with high certainty of evidence for manual therapy compared with usual care and education.

For reducing pain intensity, the most efficacious interventions at immediate-term follow-up (close to 1 week) were heat wrap, manual therapy, exercise, NSAIDS and opioids, whereas at short-term follow-up (closest to 1 month), the most efficacious treatment was manual therapy. For reducing disability, similar findings are found in the subgroup analysis showing that heat wrap, manual therapy, exercise and education for non-pharmacological group and muscle relaxants and NSAIDs for pharmacological group are effective at immediate-term follow-up. Manual therapy confirmed the effects also for decreasing disability at short-term follow-up (closest to 1 month). Limited evidence was found for steroids when compared with NSAIDs (one study) to reduce disability.

The present analysis highlights a potentially minor role for medicines in the management of NS-LBP: initial treatment should be non-pharmacological as confirmed by the SUCRA. However, only a minority of pharmacological interventions are included in the networks. In particular, steroids and opioids are under-represented (only three studies) and their desirable effects should be weighed against side effects. In fact, mild or moderate adverse events were most often recorded for the opioids, the NSAIDs and the steroids arms. This observation is shared by recent systematic reviews that found that at least 50% of people with NS-LBP taking opioids withdrew from the study owing to adverse events or lack of efficacy,^43 44^ with trends noted for higher harm rates and higher percentages of severe harm.^45^

Given that paracetamol offer limited or no benefit, its clinical value might be questionable. This finding is not reflected in all current guidelines, however.^9 46^ A recent Cochrane systematic review^47^ found that paracetamol does not result in better outcomes compared to placebo in people with acute low back pain reporting evidence from a large multicentre RCT included in our NMA (1652 randomised people with NS-LBP) that showed no benefit of any dose of paracetamol (until 4000 mg) compared with placebo in people with moderate intensity acute low back pain.^48^

Also, our data support the National Institute for Health and Care Excellence (NICE) 2016 guideline which recommend the use of NSAIDs for acute low back pain and weak opioids when NSAIDs are ineffective or poorly tolerated.^49^ In fact, we found significant reduction of pain and disability at 1 week for NSAIDs. The evidence associated with NSAIDs goes beyond the trials included in this analysis because they have a well‐established role in pain management.^50–52^

Moreover, two recent systematic reviews that found evidence for reducing pain and disability with the use of muscle relaxants recommended caution in interpretation of the findings as the evidence cannot be generalised because only two muscle relaxants were studied.^43 53^ Our analysis included a heterogeneous group of muscle relaxants (carisoprodol, thiocolchicoside, tizanidine) administered at different doses and for a short time.

Although the authors of previous published systematic reviews on spinal manipulation,^54–57^ exercise^58^ and heat wrap^59 60^ did not conduct NMA, their results overlap with ours: exercise (eg, motor control exercise, McKenzie exercise), heat wrap and manual therapy (eg, spinal manipulation, mobilisation, trigger points or any other technique) were found to reduce pain intensity and disability in adults with acute and subacute phases of NS-LBP. Such treatments should be tailored to the patient’s needs and preferences. In fact, in our analysis, there was large variability in delivering the interventions for each node. A gap exists between the current scientific literature on NS-LBP and the global actions undertaken to contrast musculoskeletal disorders.^61 62^ To date, the largest discrepancies between RCTs and global care initiatives are the new directions in classification systems, which have changed from time contingent (acute, subacute, chronic) to risk contingent (class 0–class V).^61^ In the new frameworks, treatment is targeted to the whole spine and prescribed according to the patient’s risk class regardless of the time since NS-LBP onset.^62^ A direct consequence in meta-analysing the results of RCTs, in which enrolment is mainly time-contingent based, is that patients in different risk classes may be assigned to the same group, potentially confounding the effects of the intervention. This limitation might explain the inconsistency of the results from the RCTs included in our NMA, as well as in future secondary analyses for a long time to come.

We noted other limitations in our analysis. We excluded head-to-head comparisons of the same intervention since we did not aim to inspect different characteristics of delivery (eg, intensity, dose, techniques). This was an example of our narrow inclusion criteria, set at the protocol stage, in order to obtain a homogenous sample, preventing intransitivity.^63^ Nevertheless, the studies were published over a 40-year period, during which the characteristics of interventions undoubtedly changed and thus created heterogeneity. We incorporated the certainty of evidence in the main results to highlight the most robust findings for further use in clinical judgement. We inspected potentially important clinical and demographical modifiers of treatment response at the individual patient level (eg, stage of low back pain, presence of leg pain or sciatica, mean age, percentage of male participants, baseline severity, length of treatment, number of randomised subjects and psychological assessment). We found inconsistency at 1 month for disability that remained unresolved despite exploring different strategies to resolve it. We appraised no important limitation in the transitivity evaluation even if few potential confounders were poorly reported (eg, psychological assessment) and unobserved covariates could possibly affect the global assessment.^26^ Our results should be cautiously interpreted: people with NS-LBP subgroups with different characteristics could play an important role, though such did not emerge from our analyses. We found some large estimates at immediate-term for pain that could inflate the overall effects. Small sample size (around 24% of the included trials had a sample smaller than 30 patients per arm) and study limitation (such as inadequate reporting data) could lead to doubtful pairwise estimates.

We addressed clinically important endpoints for recovery from episodes of low back pain; however, we did not include other endpoints possibly relevant for people with NS-LBP, such as health-related quality of life, social participation or return to work.^64^ Further studies should broaden outcome evaluation. Furthermore, we did not explore the combination of interventions with multidisciplinary approaches often provided in clinical settings.^65^ Taken together, the data from our NMA indicate potential successful treatments, along with ineffective interventions that contribute to waste of time and resources.

## Conclusion

Ultimately, understanding the balance between benefits and harms of non-pharmacological and pharmacological interventions is a key step to better serving people with NS-LBP. After first line of care, NS-LBP should be managed with non-pharmacological treatments which seem to mitigate pain and disability in the first week. Among pharmacological interventions, NSAIDs and muscle relaxants appear to offer the best net balance at immediate-term for pain and disability.

**What is already known**

- Non-specific low back pain (NS-LBP) is a leading cause of pain and disability worldwide.
- Among the therapeutic interventions for NS-LBP, it is not clear which intervention offers the best benefit–harm balance.
- Uncertainty in the management of NS-LBP is reflected in the often discordant guideline recommendations.

**What are the new findings**

- Among non-pharmacological interventions, pain and disability reduction were best achieved by heat wrap, manual therapy and exercise at immediate-term of follow-up.
- Among pharmacological interventions, pain and disability reduction were best achieved by NSAIDs and muscle relaxants at immediate-term of follow-up.
- Paracetamol had no benefit over inert treatments at any follow-up assessment; evidence was largely uncertain.

10.1136/bjsports-2020-103596.supp2 Supplementary data

## Data Availability

Data are available in a public, open access repository: https://osf.io/q24xh
