# Supplementary material for: Effectiveness of treatments for acute and subacute mechanical non-specific low back pain: a systematic review with network meta-analysis
Source: Br J Sports Med. 2021 Apr 13;56(1):41–50. doi: 10.1136/bjsports-2020-103596 (PMC8685632; doi:10.1136/bjsports-2020-103596)
Supplement: Supplementary data [file bjsports-2020-103596supp001.pdf]

# Effectiveness of treatments for acute and subacute mechanical non-specific low back pain: a systematic review with network meta-analysis

## Supplementary materials

**Supplement A. PRISMA NMA Checklist** ... 5
**Supplement B. Difference between protocol and review** ... 8
**Supplement C. References of Included Studies** ... 9
**Supplement D. Interventions and Nodes** ... 12
Box 1**.** Planned description interventions ... 12
Box 2. Nodes ... 13
**Supplement E. Assessment of transitivity** ... 15
Assessment of transitivity by trials ... 16
Table 1. Study and Patient characteristics (n=46) ... 16
Assessment of transitivity by interventions ... 21
Table 2. Stage of LBP ... 21
Table 3. Presence of leg pain or sciatica ... 22
Figure 1. Mean age ... 23
Figure 2. Percentage of male participants ... 23
Figure 3. Baseline severity (pain) ... 24
Figure 4. Length of treatment ... 24
Figure 5. Number of randomized ... 25
Table 4. Pshycological assessment ... 26
Assessment of transitivity by head-to-head comparisons ... 27
Table 5. Stage of LBP ... 27
Table 6. Presence of leg pain or sciatica ... 28
Figure 6. Mean age ... 29
Figure 7. Percentage of male participants ... 29
Figure 8. Baseline severity (pain) ... 30
**Supplement F. Risk of Bias** ... 32
Figure 1. Aggregate Cochrane Risk-of-bias appraisal results ... 32
Table 1. Cochrane Risk-of-bias global judgement ... 33
**Supplement G. Network Plots** ... 35
Figure 1. Network Plot- Pain outcome ... 35
Figure 1a. Network for pain outcome at 1 month of FU ... 35
Figure 1b. Network for pain outcome at 3-6 months of FU ... 35

Figure 1c. Network for pain outcome at 12 months of FU ..... 36
Figure 2. Network Plot- Disability outcome ..... 37
Figure 2a. Network for disability outcome at 1 month of FU ..... 37
Figure 2b. Network for disability outcome at 3-6 months of FU ..... 37
Figure 2c. Network for disability outcome at 12 months of FU ..... 38
**Supplement H. Assessment of pairwise Meta-Analyses** ..... 39
Pairwise meta-analyses –Pain Outcome ..... 39
Table 1. Pairwise meta-analyses at 1 week of FU for pain ..... 39
Table 2. Pairwise meta-analyses at 1 month of FU for pain ..... 39
Table 3. Pairwise meta-analyses at 3-6 months of FU for pain ..... 40
Table 4. Pairwise meta-analyses at 12 months of FU for pain ..... 40
Pairwise meta-analyses – Disability Outcome ..... 41
Table 5. Pairwise meta-analyses at 1 week of FU for disability ..... 41
Table 6. Pairwise meta-analyses at 1 month of FU for disability ..... 41
Table 7. Pairwise meta-analyses at 3-6 months of FU for disability ..... 42
Table 8. Pairwise meta-analyses at 12 months of FU for disability ..... 42
**Supplement I. Forest plot of network meta-analysis (network forest)** ..... 43
Figure 1. Network forest – pain outcome 1 week ..... 43
Figure 2. Network forest – pain outcome 1 month ..... 43
Figure 3. Network forest – pain outcome 12 months ..... 44
Figure 4. Network forest – disability outcome 1 week ..... 44
Figure 5. Network forest – disability outcome 1 month ..... 45
Figure 6. Network forest – disability outcome 12 months ..... 45
**Supplement J. Incoherence estimation and evaluation** ..... 46
Table 1. Estimated Global Inconsistency in Networks ..... 46
Table 2. Estimated Local Inconsistency for each pairwise comparison (side splitting) – pain outcome ..... 46
Table 2a. Nodesplit pain 1 week ..... 46
Table 2b. Nodesplit pain 1 month ..... 47
Table 2c. Nodesplit pain 12 months ..... 48
Table 3. Estimated Local Inconsistency for each pairwise comparison (side splitting) – disability outcome ..... 48
Table 3a. Nodesplit disability 1 week ..... 48
Table 3b. Nodesplit disability 1 month ..... 50
Table 3c. Nodesplit disability 12 months ..... 50
Table 4. Strategy to explore global inconsistency – disability 1 week ..... 51
Table 5. Strategy to explore global inconsistency – disability 1 month ..... 52

Table 6a. Metaregression disability 1 week ........ 53
Table 6b. Metaregression disability 1 month ........ 53
Figure 1. Bubble plot disability 1 week ........ 54
Figure 2. Bubble plot disability 1 month ........ 55
**Supplementary K. Subgroup analysis results** ........ 56
1. Subgroup meta-analysis (pharmacological and non-pharmacological) ........ 56
Disability 1 week – non pharmacological treatments ........ 56
Figure 1a. Network plot of non-pharmacological treaments ........ 56
Figure 2a. Network forest of non-pharmacological treaments ........ 56
Table 1a. Netleague of non-pharmacological treaments ........ 57
Table 2a. SUCRA of non-pharmacological treaments ........ 57
Disability 1 week – pharmacological treatments ........ 58
Figure 1b. Network plot of pharmacological treaments ........ 58
Figure 2b. Network forest of pharmacological treaments ........ 58
Table 1b. Netleague of pharmacological treaments ........ 59
Table 2b. SUCRA of pharmacological treaments ........ 59
Disability 1 month – non pharmacological treatments ........ 60
Figure 3a. Network plot of non-pharmacological treaments ........ 60
Figure 4a. Network forest of non-pharmacological treaments ........ 60
Disability 1 month – pharmacological treatments ........ 62
Figure 3b. Network plot of pharmacological treaments ........ 62
Figure 4b. Network forest of pharmacological treaments ........ 62
**Supplementary L. Network meta-analysis results- Interval plot** ........ 63
Figure 1. Interval Plot -Network Meta-Analyses – Pain outcome ........ 63
Figure 1a. Interval plot all treatments against inert treatment for pain outcome at 1 month of FU ........ 63
Figure 1b. Interval plot all treatments against inert treatment for pain outcome at 12 months of FU ........ 63
Figure 2. Interval Plot -Network Meta-Analyses – Disability Outcome ........ 64
Figure 2a. Interval plot all treatments against inert treatment for disability outcome at 12 months of FU ........ 64
**Supplement M. All treatments against all treatments** ........ 65
Table 1. League table - pain ........ 65
Table 1a. League table pain 1 month ........ 65
Table 1b. League table pain 12 months ........ 65
Table 2. Pain SUCRA ........ 66
Figure 1. Cumulative ranking curve of pain 1 week ........ 67
Figure 2. Cumulative ranking curve of pain 1 month ........ 67

Figure 3. Cumulative ranking curve of pain 12 months ..... 68
Table 3a. League table disability 12 months ..... 69
Table 4. Disability SUCRA ..... 70
Figure 4. Cumulative ranking curve of disability 12 months ..... 71
**Supplement N. Funnel Plot** ..... 72
Figure 1. Funnel plot-pain ..... 72
Figure 1a. Pain Outcome 1 week ..... 72
Figure 1b. Pain Outcome 1 month ..... 73
Figure 2. Funnel plot- disability ..... 74
Figure 2a. Disability Outcome 1 week ..... 74
Figure 2b. Disability Outcome 1 month ..... 75
**Supplement O. Contribution matrix for the network on interventions** ..... 76
Figure 1. Contribution matrix for the network on interventions - Pain ..... 76
Figure 1a. Contribution matrix for the network on interventions Pain Outcome 1 week ..... 77
Figure 1b. Contribution matrix for the network on interventions Pain Outcome 1 month ..... 78
Figure 1c. Contribution matrix for the network on interventions Pain Outcome 12 months ..... 79
Figure 2. Contribution matrix for the network on interventions - Disability ..... 80
Figure 2a. Contribution matrix for the network on interventions Disability Outcome 12 months ..... 80
**Supplement P. GRADE for Pain Outcome** ..... 81
1) Pain at 1 week ..... 82
2) Pain at 1 month ..... 87
3) Pain at 12 months ..... 92
**Supplement Q. GRADE for Disability Outcome** ..... 95
1) Disability at 12 months ..... 96
**Supplement R. Data check** ..... 99
**Supplement S. References** ..... 100

# Supplement A. PRISMA NMA Checklist

| Section/Topic | Item # | Checklist Item | Reported on Page # |
|---|---|---|---|
| **TITLE** | | | |
| Title | 1 | Identify the report as a systematic review *incorporating a network meta-analysis (or related form of meta-analysis).* | *1* |
| **ABSTRACT** | | | |
| Structured summary | 2 | Provide a structured summary including, as applicable:<br>**Background:** main objectives<br>**Methods:** data sources; study eligibility criteria, participants, and interventions; study appraisal; and *synthesis methods, such as network meta-analysis.*<br>**Results:** number of studies and participants identified; summary estimates with corresponding confidence/credible intervals; *treatment rankings may also be discussed. Authors may choose to summarize pairwise comparisons against a chosen treatment included in their analyses for brevity.*<br>**Discussion/Conclusions:** limitations; conclusions and implications of findings.<br>**Other:** primary source of funding; systematic review registration number with registry name. | 2 |
| **INTRODUCTION** | | | |
| Rationale | 3 | Describe the rationale for the review in the context of what is already known*, including mention of why a network meta-analysis has been conducted.* | ***4*** |
| Objectives | 4 | Provide an explicit statement of questions being addressed, with reference to participants, interventions, comparisons, outcomes, and study design (PICOS). | 4 |
| **METHODS** | | | |
| Protocol and registration | 5 | Indicate whether a review protocol exists and if and where it can be accessed (e.g., Web address); and, if available, provide registration information, including registration number. | 4 |
| Eligibility criteria | 6 | Specify study characteristics (e.g., PICOS, length of follow-up) and report characteristics (e.g., years considered, language, publication status) used as criteria for eligibility, giving rationale. *Clearly describe eligible treatments included in the treatment network, and note whether any have been clustered or merged into the same node (with justification).* | ***5*** |
| Information sources | 7 | Describe all information sources (e.g., databases with dates of coverage, contact with study authors to identify additional studies) in the search and date last searched. | 5 |
| Search | 8 | Present full electronic search strategy for at least one database, including any limits used, such that it could be repeated. | 5 |
| Study selection | 9 | State the process for selecting studies (i.e., screening, eligibility, included in systematic review, and, if applicable, included in the meta-analysis). | 5 |
| Data collection process | 10 | Describe method of data extraction from reports (e.g., piloted forms, independently, in duplicate) and any processes for obtaining and confirming data from investigators. | 5-6 |



Gianola S, *et al. Br J Sports Med* 2021; 56:41–50. doi: 10.1136/bjsports-2020-103596

| | | | |
|---|---|---|---|
| Data items | 11 | List and define all variables for which data were sought (e.g., PICOS, funding sources) and any assumptions and simplifications made. | 5-6 |
| **Geometry of the network** | **S1** | Describe methods used to explore the geometry of the treatment network under study and potential biases related to it. This should include how the evidence base has been graphically summarized for presentation, and what characteristics were compiled and used to describe the evidence base to readers. | ***7*** |
| Risk of bias within individual studies | 12 | Describe methods used for assessing risk of bias of individual studies (including specification of whether this was done at the study or outcome level), and how this information is to be used in any data synthesis. | 6 |
| Summary measures | 13 | State the principal summary measures (e.g., risk ratio, difference in means). *Also describe the use of additional summary measures assessed, such as treatment rankings and surface under the cumulative ranking curve (SUCRA) values, as well as modified approaches used to present summary findings from meta-analyses.* | 7 |
| Planned methods of analysis | 14 | Describe the methods of handling data and combining results of studies for each network meta-analysis. This should include, but not be limited to:<br>• *Handling of multi-arm trials;*<br>• *Selection of variance structure;*<br>• *Selection of prior distributions in Bayesian analyses; and*<br>• *Assessment of model fit.* | 7 |
| **Assessment of Inconsistency** | **S2** | Describe the statistical methods used to evaluate the agreement of direct and indirect evidence in the treatment network(s) studied. Describe efforts taken to address its presence when found. | 7 |
| Risk of bias across studies | 15 | Specify any assessment of risk of bias that may affect the cumulative evidence (e.g., publication bias, selective reporting within studies). | **7** |
| Additional analyses | 16 | Describe methods of additional analyses if done, indicating which were pre-specified. This may include, but not be limited to, the following:<br>• Sensitivity or subgroup analyses;<br>• Meta-regression analyses;<br>• *Alternative formulations of the treatment network; and*<br>• *Use of alternative prior distributions for Bayesian analyses (if applicable).* | ***7*** |
| **RESULTS†** | | | |
| Study selection | 17 | Give numbers of studies screened, assessed for eligibility, and included in the review, with reasons for exclusions at each stage, ideally with a flow diagram. | 8 |
| **Presentation of network structure** | **S3** | Provide a network graph of the included studies to enable visualization of the geometry of the treatment network. | ***10-11*** |
| **Summary of network geometry** | **S4** | Provide a brief overview of characteristics of the treatment network. This may include commentary on the abundance of trials and randomized patients for the different interventions and pairwise comparisons in the network, gaps of evidence in the treatment network, and potential biases reflected by the network structure. | ***10-11*** |

6

| | | | |
|---|---|---|---|
| Study characteristics | 18 | For each study, present characteristics for which data were extracted (e.g., study size, PICOS, follow-up period) and provide the citations. | 8 |
| Risk of bias within studies | 19 | Present data on risk of bias of each study and, if available, any outcome level assessment. | 10 |
| Results of individual studies | 20 | For all outcomes considered (benefits or harms), present, for each study: 1) simple summary data for each intervention group, and 2) effect estimates and confidence intervals. *Modified approaches may be needed to deal with information from larger networks.* | ***10-11*** |
| Synthesis of results | 21 | Present results of each meta-analysis done, including confidence/credible intervals. *In larger networks, authors may focus on comparisons versus a particular comparator (e.g. placebo or standard care), with full findings presented in an appendix. League tables and forest plots may be considered to summarize pairwise comparisons.* If additional summary measures were explored (such as treatment rankings), these should also be presented. | ***10-11-14*** |
| **Exploration for inconsistency** | **S5** | Describe results from investigations of inconsistency. This may include such information as measures of model fit to compare consistency and inconsistency models, *P* values from statistical tests, or summary of inconsistency estimates from different parts of the treatment network. | ***10-11*** |
| Risk of bias across studies | 22 | Present results of any assessment of risk of bias across studies for the evidence base being studied. | 10-11 |
| Results of additional analyses | 23 | Give results of additional analyses, if done (e.g., sensitivity or subgroup analyses, meta-regression analyses*, alternative network geometries studied, alternative choice of prior distributions for Bayesian analyses,* and so forth). | ***10-11*** |
| **DISCUSSION** | | | |
| Summary of evidence | 24 | Summarize the main findings, including the strength of evidence for each main outcome; consider their relevance to key groups (e.g., healthcare providers, users, and policy-makers). | 16-17 |
| Limitations | 25 | Discuss limitations at study and outcome level (e.g., risk of bias), and at review level (e.g., incomplete retrieval of identified research, reporting bias). *Comment on the validity of the assumptions, such as transitivity and consistency. Comment on any concerns regarding network geometry (e.g., avoidance of certain comparisons).* | 18 |
| Conclusions | 26 | Provide a general interpretation of the results in the context of other evidence, and implications for future research. | 18 |
| **FUNDING** | | | |
| Funding | 27 | Describe sources of funding for the systematic review and other support (e.g., supply of data); role of funders for the systematic review. This should also include information regarding whether funding has been received from manufacturers of treatments in the network and/or whether some of the authors are content experts with professional conflicts of interest that could affect use of treatments in the network. | ***19*** |

PICOS = population, intervention, comparators, outcomes, study design.

* Text in italics indicates wording specific to reporting of network meta-analyses that has been added to guidance from the PRISMA statement.

† Authors may wish to plan for use of appendices to present all relevant information in full detail for items in this section.

## Supplement B. Difference between protocol and review

We extracted some important intervention details as suggested by the TIDieR checklist [1] in order to create consistent nodes, however, the poor reporting of included trials prevent the full reporting of their descriptions. We summarized some items in Table 1 of **Supplement E** (Assessment of transitivity) and full details are reported in the online repository OSF at the following link https://osf.io/q24xh.

We transparently edit the nodes according to the statement declaration in the published protocol [2]. For instance, we build a new subgroup category “heat wrap” separated from “physical therapy” category. We also noted that “physical therapy” is represented only by TENS improving the homogeneity of treatment’s node. Then, we merged “Inert treatment” (e.g., placebo drug, sham therapy) and “No treatment” since only one study (Malmivaara 1995) reported no intervention in this control group described as: “the continuation of ordinary activities as tolerated.”

# Supplement C. References of Included Studies


1 Amlie, E., Weber, H. & Holme, I. Treatment of acute low-back pain with piroxicam: results of a double-blind placebo-controlled trial. Spine 12, 473-476 (1987).

2 Bergquist-Ullman, M. & Larsson, U. Acute low back pain in industry. A controlled prospective study with special reference to therapy and confounding factors. Acta orthopaedica scandinavica 48, 1-117 (1977).

3 Berry, H. & Hutchinson, D.R. A multicentre placebo-controlled study in general practice to evaluate the efficacy and safety of tizanidine in acute low-back pain. The Journal of international medical research 16, 75-82 (1988).

4 Bertalanffy, A., Kober, A., Bertalanffy, P., et al. Transcutaneous electrical nerve stimulation reduces acute low back pain during emergency transport. Academic emergency medicine : official journal of the Society for Academic Emergency Medicine 12, 607-611 (2005).

5 Casale R. Sintomatic treatment with a muscle relaxant drug. The Clinical journal of pain.1988 (4):81-88.

6 Cherkin, D.C., Deyo, R.A., Street, J.H., Hunt, M. & Barlow, W. Pitfalls of patient education. Limited success of a program for back pain in primary care. Spine 21, 345-355 (1996).

7 Cherkin, D.C., Deyo, R.A., Battie, M., Street, J. & Barlow, W. A comparison of physical therapy, chiropractic manipulation, and provision of an educational booklet for the treatment of patients with low back pain. The New England journal of medicine 339, 1021-1029 (1998).

8 Dapas, F., Hartman, S.F., Martinez, L., et al. Baclofen for the treatment of acute low-back syndrome. A double-blind comparison with placebo. Spine 10, 345-349 (1985).

9 Dreiser, R.L., Marty, M., Ionescu, E., Gold, M. & Liu, J.H. Relief of acute low back pain with diclofenac-K 12.5 mg tablets: a flexible dose, ibuprofen 200 mg and placebo-controlled clinical trial. International journal of clinical pharmacology and therapeutics 41, 375-385 (2003).

10 Eken, C., Serinken, M., Elicabuk, H., Uyanik, E. & Erdal, M. Intravenous paracetamol versus dexketoprofen versus morphine in acute mechanical low back pain in the emergency department: a randomised double-blind controlled trial. Emergency medicine journal : EMJ 31, 177-181 (2014).

11 Eskin, B., Shih, R.D., Fiesseler, F.W., et al. Prednisone for emergency department low back pain: a randomized controlled trial. The Journal of emergency medicine 47, 65-70 (2014).

12 Faas, A., van Eijk, J.T., Chavannes, A.W. & Gubbels, J.W. A randomized trial of exercise therapy in patients with acute low back pain. Efficacy on sickness absence. Spine 20, 941-947 (1995).

13 Goldie, I. A clinical trial with indomethacin (indomee(R)) in low back pain and sciatica. Acta orthopaedica Scandinavica 39, 117-128 (1968).

14 Haimovic, I.C. & Beresford, H.R. Dexamethasone is not superior to placebo for treating lumbosacral radicular pain. Neurology 36, 1593-1594 (1986).

15 Hasegawa, T.M., Baptista, A.S., de Souza, M.C., Yoshizumi, A.M. & Natour, J. Acupuncture for acute non-specific low back pain: a randomised, controlled, double-blind, placebo trial. Acupuncture in medicine: journal of the British Medical Acupuncture Society 32, 109-115 (2014).

16 Hindle, T.H., 3rd. Comparison of carisoprodol, butabarbital, and placebo in treatment of the low back syndrome. California medicine 117, 7-11 (1972).

17 Jellema, P., van der Windt, D.A., van der Horst, H.E., Twisk, J.W., Stalman, W.A. & Bouter, L.M. Should treatment of (sub)acute low back pain be aimed at psychosocial prognostic factors? Cluster randomised clinical trial in general practice. BMJ (clinical research ed.) 331, 84 (2005).

18 Ketenci, A., Ozcan, E. & Karamursel, S. Assessment of efficacy and psychomotor performances of thiocolchicoside and tizanidine in patients with acute low back pain. International journal of clinical practice 59, 764-770 (2005).

19 Kettenmann, B., Wille, C., Lurie-Luke, E., Walter, D. & Kobal, G. Impact of continuous low level heatwrap therapy in acute low back pain patients: subjective and objective measurements. The Clinical journal of pain 23, 663-668 (2007).

20 Lindstrom, I., Ohlund, C. & Nachemson, A. Physical performance, pain, pain behavior and subjective disability in patients with subacute low back pain. Scandinavian journal of rehabilitation medicine 27, 153-160 (1995).

21 Malmivaara, A., Hakkinen, U., Aro, T., et al. The treatment of acute low back pain--bed rest, exercises, or ordinary activity? The New England journal of medicine 332, 351-355 (1995).

22 Mayer, J.M., Ralph, L., Look, M., et al. Treating acute low back pain with continuous low-level heat wrap therapy and/or exercise: a randomized controlled trial. The spine journal : official journal of the North American Spine Society 5, 395-403 (2005).

23 Miki, K., Ikemoto, T., Hayashi, K., et al. Randomized open-labbel non-inferiority trial of acetaminophen or loxoprofen for patients with acute low back pain. Journal of orthopaedic science : official journal of the Japanese Orthopaedic Association 23, 483-487 (2018).

24 Nadler, S.F., Steiner, D.J., Erasala, G.N., et al. Continuous low-level heat wrap therapy provides more efficacy than Ibuprofen and acetaminophen for acute low back pain. Spine 27, 1012-1017 (2002).

25 Nadler, S.F., Steiner, D.J., Petty, S.R., Erasala, G.N., Hengehold, D.A. & Weingand, K.W. Overnight use of continuous low-level heatwrap therapy for relief of low back pain. Archives of physical medicine and rehabilitation 84, 335-342 (2003).

26 Nadler, S.F., Steiner, D.J., Erasala, G.N., Hengehold, D.A., Abeln, S.B. & Weingand, K.W. Continuous low-level heatwrap therapy for treating acute nonspecific low back pain. Archives of physical medicine and rehabilitation 84, 329-334 (2003).

27 Postacchini, F., Facchini, M. & Palieri, P. Efficacy of various forms of conservative treatment in low back pain. A comparative study. Neuro-orthopedics 6, 28-35 (1988).

28 Ralph, L., Look, M., Wheeler, W. & Sacks, H. Double-blind, placebo-controlled trial of carisoprodol 250-mg tablets in the treatment of acute lower-back spasm. Current medical research and opinion 24, 551-558 (2008).

29 Sae-Jung, S. & Jirarattanaphochai, K. Outcomes of lumbar facet syndrome treated with oral diclofenac or methylprednisolone facet injection: a randomized trial. International orthopaedics 40, 1091-1098 (2016).

30 Santilli, V., Beghi, E. & Finucci, S. Chiropractic manipulation in the treatment of acute back pain and sciatica with disc protrusion: a randomized double-blind clinical trial of active and simulated spinal manipulations. The spine journal : official journal of the North American Spine Society 6, 131-137 (2006).

31 Schenk, R.J., Jozefczyk, C. & Kopf, A. A randomized trial comparing interventions in patients with lumbar posterior derangement. Journal of manual & manipulative therapy 11, 95-102 (2003).

32 Schneider, M., Haas, M., Glick, R., Stevans, J. & Landsittel, D. Comparison of spinal manipulation methods and usual medical care for acute and subacute low back pain: a randomized clinical trial. Spine 40, 209-217 (2015).

33 Seferlis, T., Nemeth, G., Carlsson, A.M. & Gillstrom, P. Conservative treatment in patients sick-listed for acute low-back pain: a prospective randomised study with 12 months' follow-up. European spine journal : official publication of the European Spine Society, the European Spinal Deformity Society, and the European Section of the Cervical Spine Research Society 7, 461-470 (1998).

34 Serfer, G.T., Wheeler, W.J. & Sacks, H.J. Randomized, double-blind trial of carisoprodol 250 mg compared with placebo and carisoprodol 350 mg for the treatment of low back spasm. Current Medical Research and Opinion 26, 91-99 (2010).

35 Shin, J.S., Ha, I.H., Lee, J., et al. Effects of motion style acupuncture treatment in acute low back pain patients with severe disability: a multicenter, randomized, controlled, comparative effectiveness trial. Pain 154, 1030-1037 (2013).

36 Storheim, K., Brox, J.I., Holm, I., Koller, A.K. & Bo, K. Intensive group training versus cognitive intervention in sub-acute low back pain: short-term results of a single-blind randomized controlled trial. Journal of rehabilitation medicine 35, 132-140 (2003).

37 Suni, J., Rinne, M., Natri, A., Statistisian, M.P., Parkkari, J. & Alaranta, H. Control of the lumbar neutral zone decreases low back pain and improves self-evaluated work ability: a 12-month randomized controlled study. Spine 31, E611-620 (2006).

38 Szpalski, M. & Hayez, J.P. Objective functional assessment of the efficacy of tenoxicam in the treatment of acute low back pain. A double-blind placebo-controlled study. British journal of rheumatology 33, 74-78 (1994).

39 Takamoto, K., Bito, I., Urakawa, S., et al. Effects of compression at myofascial trigger points in patients with acute low back pain: A randomized controlled trial. European journal of pain (London, England) 19, 1186-1196 (2015).

40 Traeger, A.C., Lee, H., Hübscher, M., et al. Effect of Intensive Patient Education vs Placebo Patient Education on Outcomes in Patients with Acute Low Back Pain: A Randomized Clinical Trial. JAMA Neurology 76, 161-169 (2019).

41 Tuzun, F., Unalan, H., Oner, N., et al. Multicenter, randomized, double-blinded, placebo-controlled trial of thiocolchicoside in acute low back pain. Joint, bone, spine : revue du rhumatisme 70, 356-361 (2003).

42 Veenema, K.R., Leahey, N. & Schneider, S. Ketorolac versus meperidine: ED treatment of severe muskuloskeletal low back pain. American Journal of Emergency Medicine 18, 404-407 (2000).

43 Videman, T., Heikkila, J. & Partanen, T. Double-blind parallel study of meptazinol versus diflunisal in the treatment of lumbago. Current medical research and opinion 9, 246-252 (1984).

44 von Heymann, W.J., Schloemer, P., Timm, J. & Muehlbauer, B. Spinal high-velocity low amplitude manipulation in acute nonspecific low back pain: a double-blinded randomized controlled trial in comparison with diclofenac and placebo. Spine 38, 540-548 (2013).

45 Williams, C.M., Maher, C.G., Latimer, J., et al. Efficacy of paracetamol for acute low-back pain: a double-blind, randomised controlled trial. Lancet (London, England) 384, 1586-1596 (2014).

46 Younes, M., Nowakowski, K., Didier-Laurent, B., Gombert, M. & Cottin, F. Effect of spinal manipulative treatment on cardiovascular autonomic control in patients with acute low back pain. Chiropractic & manual therapies 25, 33 (2017).

# Supplement D. Interventions and Nodes

## Box 1. Planned description interventions

| Class | Example of individual treatments |
|---|---|
| **Pharmacological** | |
| Antidepressant drugs | Any kind of SSRI/SNRI or tryciclic drug |
| Muscle relaxants drugs | Any kind of skeletal muscle relaxant drug (e.g. flupirtin, orphenadrine, dantrolene, carisoprodol, tizanidine, incobotulinumtoxinA, cyclobenzaprine, metaxalone, baclofen, methocarbamol, chlorzoxazone) |
| Non-steroidal anti-inflammatory drugs (NSAIDs) | Any kind of NSAIDs drug, including COX-2 inhibitors (e.g. ibuprofen, naproxen, sulindac, ketoprofen, tolmetin, etodolac, fenoprofen, diclofenac, flurbiprofen, piroxicam, ketorolac, indomethacin, meloxicam, nabumetone, oxaprozin mefenamic acid, diflunisal) |
| Opiod drugs | Any kind of strong or weak opiod analgesics (e.g. morphine, hydromorphone, oxycodone, fentanyl, methadone, buprenorphine, diamorphine, tapentadol, codeine, hydrocodone, tramadol, pentazocine, tilidine) |
| Paracetamol | |
| Steroids | Any kind of steroid drug (e.g dexamethasone, methylprednisolone, prednisone) |
| **Non-pharmacological treatments** | |
| Acupuncture and dry needling | |
| Biopsychosocial rehabilitation | Any kind of cognitive behavioral treatment, multidisciplinary biopsychological rehabilitation and back school |
| Education | Any kind of advice to stay active, booklet, reassurance, ergonomics, workplace intervention, pain education (neurobiology and neurophysiology of pain) |
| Exercise | Any kind of exercise (aerobic or resistance training) single supervised or home exercise, including stretching and McKenzie therapy |
| Manual therapy | Any kind of mobilization or spinal manipulation (high velocity thrust techniques at or near to the end of the range of motion or low-grade velocity movements within the range of motion), myofascial therapy/trigger point, soft tissue massage |
| Physical Therapy | Any physical therapy (low-laser therapy, diathermy, transcutaneous electrical nerve stimulation, ultrasound therapy, heat wrap) |
| Taping | Kinesiotaping |
| Usual care | Any kind of treatment suggested by general medicine (minimal intervention: advice to stay active or to take drugs as needed) |
| Inert treatment | Any kind of sham or placebo therapy |
| No treatment | No treatment, waiting list control |



Gianola S, *et al. Br J Sports Med* 2021; 56:41–50. doi: 10.1136/bjsports-2020-103596

# Box 2. Nodes

| Treatments | Nodes | Evidence and assumptions |
|---|---|---|
| Muscle relaxant drugs (Baclofen, Carisoprodol, Dantrolene, Tizanidine Thiocolchicoside) | Muscle relaxant | Separate assessment for muscle relaxants and for Benzodiazepines[3].<br>A metanalysis shown similar effects across muscle relaxant drugs versus placebo, $I^2$=55%[4]. |
| Non-Steroidal Anti-Inflammatory Drugs (NSAIDs), including COX-2 inhibitors (diclofenac, diflunisal, ibuprofen, indomethacin, loxoprofen, piroxicam, tenoxicam) | NSAIDs | Separate assessment for all NSAIDs[3].<br>No clear difference in short-term pain reduction when comparing selective COX-2 inhibitors to non-selective NSAIDs[5]. |
| Opioid analgesics (meptazinol) | Opioids | Separate assessment for opiods[3].<br>Inclusion criteria of SR: morphine, diamorphine, fentanyl, alfentanil, remifentanil, methadone, oxycodone, pethidine, tapentadol, tramadol, codeine, dihydrocodeine, meptazinol)[6].<br>Inclusion criteria of SR: various opioid analgesics[7]. |
| Paracetamol | Paracetamol | Separate assessment for paracetamol [3 8]. |
| Steroids drugs (dexamethasone, methylprednisolone, prednisone) | Steroids | Separate assessment for steroids [3].<br>Systematic reviews found no evidence to suggest that a series of epidural injections was any more effective than a single injection (see Appendix 1 Table 3). Individual RCTs found no evidence of improvement in steroid benefits with increasing dose (see Appendix 1 Table 4) [9].<br>Individual RCTs found no consistent evidence of superior efficacy of one steroid over the others (see Appendix 1 Table 4) [9].<br>A meta-analysis included all type of steroids. [10]. |
| Acupuncture | Acupuncture | |
| Cognitive behavioural treatment/multidisciplinary biopsychological rehabilitation (MBR) with or without exercise | Cognitive behavioral therapy | Inclusion criteria of Cochrane review, MBR program: the intervention included a physical component (e.g., pharmacological, physical therapy, exercise) in combination with either a psychological, social, or occupational component (or any combination of these)[11]. |
| Back school | Back school * | |
| Booklet, Information, ergonomics, any kind of advice, workplace intervention, pain education | Education | Findings suggest positive effects for education even if differ in terms of its contents such as health education, self-management, video education, and postural education[12]. |

| | | |
|---|---|---|
| | | Many different types of patient education are widely used [13]. |
| McKenzie<br>Any kind of exercise (aerobic or resistance training)<br>Stretching | Exercise | No superior type of physical exercise for people with chronic non-specific neck pain [14].<br>Various exercise training approaches are effective [15]. |
| Spinal manipulation<br>Manual therapy (mobilization)<br>Trigger point/myofascial therapy/massage | Manual therapy | Inclusion crtieria of SR: Studies investigating manual therapy using HVLA or non-HVLA techniques such as: joint mobilization, soft tissue focused techniques, myofascial release, longitudinal sliding, soft tissue mobilizations, deep-pressure massage, muscle energy, massage, hold relaxation technique, ischemic compression, and functional/fascial technique. therapy technique(s)[16].<br>Different forms of manual therapy did not lead to different outcomes in older persons with chronic LBP [17]. |
| Heat wrap | Heat wrap** | |
| TENS | Physical therapy | |
| Usual care or minimal treatment (general prescription such as drugs as needed, advice stay active) | Usual care | Usual care is a term used to describe the full spectrum of patient care practices in which clinicians have the opportunity (which is not necessarily seized) to individualize care [18].<br>Treatment reported: education and reassurance, exercise, bed rest, return to work[19]. |
| Sham therapy<br>Placebo therapy<br>No treatment | Inert treatment | |

* This node was assessed only in the qualitative synthesis because of insufficient data (e.g., not reported outcome data)
**According to the protocol [2] since we obtained a sufficient number of studies sharing the same description of the intervention, we created a new node (heat wrap) separated from the physical therapy node.

## Supplement E. Assessment of transitivity

Before conducting the statistical analysis, we assessed whether the trials included in the NMA were on average similar in terms of characteristics that might modify the treatment effect (so that the transitivity assumption is plausible). Indirect comparisons, in contrast to direct comparisons, are not protected by randomisation and may be confounded by differences between the trials. In our analysis we deemed the following parameters as possible confounders [20] which were displayed as cumulative frequencies, boxplots or bar charts when appropriate: stage of NS-LBP, presence of leg pain or sciatica, mean age, percentage of male participants, baseline severity, length of treatment, number of randomized, psychological assessment. The plausibility of the transitivity assumption was evaluated by comparing the distribution of these potential effect modifiers across trials, interventions and heah-to-head comparisons

# Assessment of transitivity by trials

## Table 1. Study and Patient characteristics (n=46)

| ID | Author | Year | Setting | Stage of LBP | Presence of leg pain or sciatica | Length of treatment | Outcomes | Week of FU | Sample size | Treatments | Nodes | Age mean | Age variance (SD) | % of male |
|---|---|---|---|---|---|---|---|---|---|---|---|---|---|---|
| **1** | Amlie* | 1987 | Multi-center | Acute LBP (less than 6 weeks) | Not stated | 1 week | Pain; disability | 3 days;<br>7 days | 282 | 1.Piroxicam<br>2. Placebo | NSAIDs<br>Inert treatment | 37,3<br>38,5 | NA | 58,6<br>59,2 |
| **2** | Bergquist-ullman* | 1977 | Single center | Mixed LBP (less than 12 weeks) | Yes | 2 weeks<br>Max 10 trt | Pain; disability | 10 days;<br>3 weeks;<br>6 weeks | 145 | 1.Back school<br>2. Placebo | Back school<br>Inert treatment | NA | NA | 91,4<br>86,7 |
| **3** | Berry | 1988 | Single center | Acute LBP (less than 6 weeks) | Yes | 1 week | Pain | 1 week | 112 | 1.Tizanidine<br>2. Placebo | Muscle relaxant<br>Inert treatment | 44<br>38 | 13<br>13 | 51<br>50,9 |
| **4** | Bertalanffy | 2005 | Single center | Acute LBP (less than 6 weeks) | No | 1 day | Pain | 30 minutes | 63 | 1. TENS<br>2. Sham TENS | Physical therapy<br>Inert treatment | 47<br>49 | 7<br>14 | 53,3<br>51,5 |
| **5** | Casale* | 1988 | Single center | Acute LBP (less than 6 weeks) | Not stated | 4 days | Pain | Day 4 | 20 | 1.Dantrolene sodium<br>2. Placebo | Muscle relaxant<br>Inert treatment | 46,7<br><br>47,1 | 2,3<br>2,2 | 70<br>80 |
| **6** | Cherkin* | 1996 | Single center | Mixed LBP (less than 12 weeks) | Yes | 1 session | Pain; disability | 1 week | 299 | 1. Nurse education<br>2. Booklet<br>3. Usual care | Education<br>Education<br>Usual care | 40,8<br>44,1<br>43,0 | NA | 57<br>49<br>51 |
| **7** | Cherkin** | 1998 | Multi-center | Mixed LBP (less than 12 weeks) | No | 1 month | Pain; disability | 4 weeks;<br>12 weeks;<br>12 months | 321 | 1. McKenzie<br>2. Manipulation<br>3. Booklet | Exercise<br>Manual therapy<br>Education | 41,8<br>39,7<br>40,1 | 11,5<br>9,4<br>11,2 | 53<br>47<br>58 |
| **8** | Dapas* | 1985 | Multi-center | Acute LBP (less than 6 weeks) | Not stated | 14 days | Pain; disability | Day 4;<br>Day 10 | 123 | 1. Baclofen<br>2. Placebo | Muscle relaxant<br>Inert treatment | 42,7<br>41,8 | NA | 52<br>44 |
| **9** | Dreiser | 2003 | Multi-center | Acute LBP (less than 6 weeks) | No | 1 week | Pain; disability | Day 3; day 8 | 372 | 1. Diclofenac-K<br>2. Ibuprofen<br>3. Placebo | NSAIDs<br>NSAIDs<br>Inert treatment | 40,9<br>40,6<br>41 | 10,9<br>11,6 | 48,4<br>52,5<br>47,2 |

| | | | | | | | | | | | | | | |
|---|---|---|---|---|---|---|---|---|---|---|---|---|---|---|
| | | | | | | | | | | | | | 11,3 | |
| **10** | Eken* | 2014 | Silgle center | Acute LBP (less than 6 weeks) | No | 1 day | Pain | 30 minutes | 137 | 1. Paracetamol<br>2. Dexketoprofen<br>3. Morphine | Paracetamol<br>NSAIDs<br>Opioid | 31,5* | 9,5* | 60,6* |
| **11** | Eskin* | 2014 | Single center | Acute LBP (less than 6 weeks) | Not stated | 5 days | Pain | Day 5-7 | 79 | 1. Prednisone<br>2. Placebo | Steroids<br>Inert treatment | 39<br>41 | 8<br>9 | 67<br>73 |
| **12** | Faas* | 1995 | Multi-center | Acute LBP (less than 6 weeks) | Yes | 5 weeks | Pain | 1 week;<br>1 month;<br>12 month | 363 | 1. Exercise<br>1. Usual care<br>2. Sham ultrasound | Exercise<br>Usual care<br>Inert treatment | 35<br>34<br>37 | NA | 62<br>71<br>66 |
| **13** | Goldie* | 1968 | Single center | Acute LBP (less than 6 weeks) | Yes | 14 days | Pain | 1 week;<br>2 weeks | 50 | 1. Indomethacin<br>2. Placebo | NSAIDs<br>Placebo | NA | NA | 52<br>52 |
| **14** | Haimovic* | 1986 | Single center | Acute LBP (less than 6 weeks) | Yes | 7 days | Pain | 1 week;<br>12 months | 33 | 1. Dexamethasone<br>2. Placebo | Steroids<br>Inert treatment | NA | NA | NA |
| **15** | Hasegawa | 2014 | Single center | Acute LBP (less than 6 weeks) | No | 1 week | Pain; disability | 7 days;<br>28 days | 80 | 1. Acupuncture<br>2. Sham acupuncture | Acupuncutre<br>Inert treament | 47<br>43,9 | 9,8<br>10,9 | 37,5<br>35 |
| **16** | Hindle* | 1972 | Single center | Acute LBP (less than 6 weeks) | Not stated | 4 days | Pain; disability | 2 days;<br>4 days | 32 | 1. Carisoprodol<br>2. Placebo | Muscle relaxant<br>Inert treatment | 37<br>43,5 | NA<br>NA | 56<br>62 |
| **17** | Jellema | 2005 | Multi-center | Mixed LBP (less than 12 weeks) | Not stated | 5 days | Pain; disability | 6, 26, 52 weeks | 314 | 1.Behavioral therapy<br>2. Usual care | Cognitive behavioral therapy<br>Usual care | 43,4<br>42 | 11,1<br>12 | 52,4<br>52,6 |
| **18** | Ketenci | 2005 | Single center | Acute LBP (less than 6 weeks) | Not stated | 1 week | Pain | Day 5-7 | 97 | 1.Thiocolchicoside<br>2. Tizanidine<br>3.Placebo | Muscle relaxant<br>Muscle relaxant<br>Inert treatment | 37<br>37<br>40 | NA<br>NA<br>NA | 57,9<br>37,5<br>48,1 |
| **19** | Kettenmann* | 2007 | Single center | Mixed LBP (less than 12 weeks) | Not stated | 4 days | Pain | Day 4 | 30 | 1. Heat wrap<br>2. Usual care | Heatwrap<br>Usual care | 56,2<br>57,9 | 14,9<br>11,7 | 46,7<br>25 |
| **20** | Lindstrom | 1995 | Single center | Subacute LBP (6-12 weeks) | Not stated | Until recovery | Pain; disability | 12 months | 103 | 1. Cognitive behavioral therapy<br>2. Usual care | Cognitive behavioral therapy<br>Usual care | 39,4<br>42,4 | 10,7<br>10,9 | 76,5<br>61,5 |

| | | | | | | | | | | | | | | |
|---|---|---|---|---|---|---|---|---|---|---|---|---|---|---|
| **21** | Malmivaara | 1995 | Multi-center | Acute LBP (less than 6 weeks) | Yes | Not reported | Pain; disability | 3 weeks; 12 weeks | 119 | 1. Exercise<br>2. No treatment | Exercise<br>Inert treatment | 41,1<br>39,1 | NA<br>NA | 29<br>30 |
| **22** | Mayer | 2005 | Multi-center | Mixed (acute and subacute) | No | 5 days | Pain; disability | 1 week | 76 | 1. Heat wrap<br>2. Exercise<br>3. Booklet | Heat wrap<br>Exercise<br>Education | 29,3<br>32,6<br>31,3 | 9,9<br>10,3<br>10,9 | 32<br>40<br>7,7 |
| **23** | Miki | 2018 | Single center | Acute LBP (less than 6 weeks) | No | 4 weeks | Pain; disability | 2 weeks, 1 month | 127 | 1. Acetaminophen<br>2. Loxoprofen | Paracetamol<br>NSAIDs | 66,7<br>63,5 | 2,3<br>19,4 | 32,8<br>34,9 |
| **24** | Nadler** | 2002 | Multi-center | Mixed (acute and subacute) | No | 2 days o 1 day?? | Pain; disability | 4 days | 371 | 1. Heat wrap<br>2. Acetaminophen<br>3. Ibuprofen<br>4. Unheated wrap<br>5. Oral placebo | Heat wrap<br>Paracetamol<br>NSAIDs<br>Inert treatment<br>Inert treatment | 35,8<br>34,9<br>36,6<br>36,8<br>38,0 | 10,5<br>11,3<br>10,4<br>9,3<br>9,1 | 41,6<br>43,4<br>40,6<br>42,1<br>40 |
| **25** | Nadler** | 2003b | Multi-center | Mixed (acute and subacute) | No | 3 days | Pain; disability | Days 2-4 | 76 | 1.Heat wrap<br>2. Oral placebo<br>3. Ibuprofen<br>4. Unheated wrap | Heat wrap<br>Inert treatment<br>NSAIDs | 42,2<br>41,5<br>42,5<br>34,0 | 9,4<br>9,8<br>2,7<br>8,4 | 36,4<br>38,2<br>25<br>20 |
| **26** | Nadler** | 2003 a | Multi-center | Mixed (acute and subacute | No | 3 days | Pain; disability | Day 5 | 219 | 1. Heat wrap<br>2. Oral placebo<br>3. Ibuprofen<br>4. Unheated wrap | Heat wrap<br>Inert treatment<br>NSAIDs<br>Inert treatment | 35,6<br>36,7<br>36,3<br>34,9 | 11,6<br>10,8<br>11,6<br>11,3 | 45,7 |
| **27 a** | Postacchini * | 1988 | Multi-center | Acute LBP (less than 6 weeks) | No | 4 weeks<br>10-14 days<br>1 or 2 weeks | Pain; disability | 3 weeks; 6 months | 46 | 1. Manipulation<br>2. Diclofenac<br>3. Placebo gel | Manual therapy<br>NSAIDs<br>Inert treatmnt | 36,3 | NA | 55 |
| **27 b** | Postacchini * | 1988 | Multi-center | Acute LBP (less than 6 weeks) | No | 4 weeks<br>10-14 days<br>1 week<br>1 or 2 weeks | Pain; disability | 3 weeks; 6 months | 66 | 1. Manipulation<br>2. Diclofenac<br>3. Back school<br>4. Placebo gel | Manual therapy<br>NSAIDs<br>Back school<br>Inert treatment | 40,3 | NA | 51,2 |
| **27 c** | Postacchini * | 1988 | Multi-center | Acute LBP (less than 6 weeks) | Yes | 4 weeks<br>10-14 days<br>1 or 2 weeks | Pain; disability | 3 weeks; 6 months | 53 | 1. Manipulation<br>2. Diclofenac<br>3. Placebo gel | Manual therapy<br>NSAIDs<br>Inert treatment | 37,7 | NA | 45,8 |

| | | | | | | | | | | | | | | |
|---|---|---|---|---|---|---|---|---|---|---|---|---|---|---|
| **28** | Ralph* | 2008 | Multi-center | Acute LBP (less than 6 weeks) | No | 7 days | Pain; disability | 1 week | 562 | 1. Carisoprodol<br>2. Placebo | Muscle relaxant<br>Inert treatment | 39,3<br>41,5 | 11,82<br>11,7 | 51,3<br>45 |
| **29** | Sae-Jung | 2016 | Single center | Mixed (acute and subacute) | No | 2 weeks | Pain; disability | 1 month; 3 months | 65 | 1. Diclofenac<br>2. Methylprednisolone | NSAIDs<br>Steroids | 49<br>44 | 8,7<br>9,3 | 55<br>53,1 |
| **30** | Santilli | 2006 | Multi-center | Acute LBP (less than 6 weeks) | Yes | Until recovery (max 4 weeks) | Pain | 15 days; 1, 3, 6 months | 102 | 1. Active manipulation<br>2. Simulated manipulation | Manual therapy<br>Inert treatment | NA | NA | 69,8<br>55,1 |
| **31** | Schrenk | 2003 | Single center | Mixed (acute and subacute) | Yes | Not reported | Pain; disability | 3 visits | 25 | 1. Exercise (McKenzie)<br>2. Mobilization | Exercise<br>Manual therapy | 40,1<br>44,8 | 17,1<br>12,7 | 46,7<br>80 |
| **32** | Schneider | 2015 | Single center | Mixed (acute and subacute) | No | 4 weeks | Pain; disability | 4 weeks; 3 months; 6 months | 112 | 1. Manual manipulation<br>2. Mechanical assisted manipulation<br>3. Usual care | Manual therapy<br>Manual therapy<br>Usual care | 41,4<br>40,4<br>41,3 | 15,3<br>15,9<br>11,6 | 32,4<br>40<br>40 |
| **33** | Seferlis | 1998 | Single center | Acute LBP (less than 6 weeks) | Yes | 8 weeks | Pain; disability | 1 months; 3 months; 12 months | 180 | 1. Exercise<br>2. General pratictionnaire program-usual care | Exercise<br>Usual care | 39 | 19-64 range | 52,7 |
| **34** | Serfer* | 2009 | Multi-center | Acute LBP (less than 6 weeks) | No | 1 week | Pain; disability | 1 week | 828 | 1.Carisoprodol 250 mg<br>2. Carisoprodol 350 mg<br>3. Placebo | Muscle relaxant<br>Muscle relaxant<br>Inert treatment | 40,9<br>40,5<br>40,7 | 11,7<br>12,4<br>13,1 | 47,7<br>44,3<br>39,4 |
| **35** | Shin | 2013 | Multi-center | Acute LBP (less than 6 weeks) | Yes | 1 day | Pain; disability | 2 weeks; 4 weeks; 24 weeks | 58 | 1. Acupuncture<br>2. Diclofenac | Acupuncture<br>NSAIDs | 37,9<br>38,7 | 7,4<br>8,6 | 66<br>52 |
| **36** | Storheim | 2003 | Single center | Subacute LBP (6-12 weeks) | No | 15 weeks<br>1 week | Pain; disability | 18 weeks; 48 weeks | 93 | 1. Exercise<br>2. Cognitive intervention<br>3. Usual care | Exercise<br>Cognitive behavioral therapy<br>Usual care | 42,3<br>41,3<br>38,9 | 9,2<br>9,4<br>11,9 | 46,7<br>52,9<br>44,8 |
| **37** | Suni* | 2006 | Multi-center | Mixed (acute and subacute) | Not stated | 12 monhts | Pain; disability | 6 months; 12 monhts | 106 | 1. Exercise with cognitive goals<br>2. Control group | Cognitive behavioral therapy<br>Usual care | 47,6<br>46,9 | 5,8<br>5,3 | 100<br>100 |
| **38** | Szpalski | 1994 | Single center | Acute LBP (less than 6 weeks) | Yes | 1-2 weeks | Pain | 8 days; 15 days | 73 | 1. Tenoxicam<br>2. Placebo | NSAIDs<br>Inert treatment | 37,5<br>38,9 | 9,2<br>10,4 | 62,2<br>66,7 |

| | | | | | | | | | | | | | | |
|---|---|---|---|---|---|---|---|---|---|---|---|---|---|---|
| **39** | Takamoto | 2015 | Multi-center | Acute LBP (less than 6 weeks) | No | 2 weeks | Pain; disability | 1 week; 1 month | 63 | 1. Compression at TP<br>2. Sham compression<br>3. Effleurage massage | Manual therapy<br>Inert treatment<br>Manual therapy | 38<br>38,1<br>35,6 | 3<br>3,8<br>3 | 45,4<br>47,1<br>37,5 |
| **40** | Traeger | 2019 | Multi-center | Acute LBP (less than 6 weeks) | Yes | 2 sessions | Pain; disability | 1 week, 3, 6, 12 months | 202 | 1. Education<br>2. Sham education | Education<br>Inert treatment | 46,5<br>43,8 | 14,7<br>14,1 | 47,5<br>50,5 |
| **41** | Tuzun | 2003 | Multi-center | Acute LBP (less than 6 weeks) | Not stated | Until recovery, max 5 days | Pain | 5 days | 149 | 1.Thiocolchicoside<br>2. Placebo | Muscle relaxant<br>Inert treatment | 40,7<br>41 | 10,3<br>11 | 50<br>42 |
| **42** | Veenema | 2000 | Single center | Acute LBP (less than 6 weeks) | Not stated | 1 day | Pain | 60 minutes | 155 | 1. Meperidine<br>2. Ketorolac | Opioid<br>NSAIDs | 35,5<br>36,0 | 12,8<br>12,1 | 63,0<br>60,0 |
| **43** | Videman* | 1984 | Single center | Acute LBP (less than 6 weeks) | No | Until recovery, max 3 weeks | Pain; disability | 1 week;<br>3 weeks | 70 | 1. Meptazinol<br>2. Diflunisal | NSAIDs<br>Opioid | 38,0<br>35,0 | 14,0<br>11,0 | 60,0<br>57,1 |
| **44** | von Heymann** | 2013 | Multi-center | Acute LBP (less than 6 weeks) | Not stated | Not reported | Pain; disability | 9 days | 100 | 1. Manipulation<br>2. Diclofenac<br>3. Placebo-sham | Manual therapy<br>NSAIDs<br>Inert treatment | 34,1*<br>37,5*<br>39,3* (median values) | 9,5<br>10,9<br>10,2 | 63,9<br>10,9<br>10,2 |
| **45** | Williams | 2014 | Multi-center | Acute LBP (less than 6 weeks) | Yes | Until recovery, max 4 weeks | Pain; disability | 1week; 1 month; 3 months; | 1652 | 1. Paracetamol<br>2. Paracetamol as needed<br>3. Placebo | Paracetamol<br>Paracetamol<br>Inert treatment | 44,1<br>45,5<br>45,4 | 14,8<br>16,7<br>15,9 | 52,0<br>53,0<br>55,0 |
| **46** | Younes* | 2017 | Single center | Mixed (acute and subacute) | Not stated | 1 week | Pain | 1 week | 22 | 1. Manipulation<br>2. Sham manipulation | Manual therapy<br>Inert treatment | 31,0<br>28,0 | 9,0<br>7,0 | 100,0<br>100,0<br>100,0 |

*studies were not included in quantitative analysis due to different reasons such as median and IQR, missing outcome data.
**not all treatment arms are reported in quantitative analysis (e.g., multi-arm trial reported 2 out 3 treatment arms with available outcome data).

# Assessment of transitivity by interventions

## Table 2. Stage of LBP

| | FREQUENCIES (%) | | |
|---|---|---|---|
| **TREATMENT** | Acute | Subacute | Mixed |
| **A** | 76,5 | 0,0 | 23,5 |
| **B** | 100,0 | 0,0 | 0,0 |
| **C** | 50,0 | 0,0 | 50,0 |
| **D** | 0,0 | 50,0 | 50,0 |
| **E** | 20,0 | 0,0 | 80,0 |
| **F** | 42,9 | 14,3 | 42,9 |
| **G** | 0,0 | 0,0 | 100,0 |
| **H** | 58,3 | 0,0 | 41,7 |
| **I** | 100,0 | 0,0 | 0,0 |
| **J** | 77,8 | 0,0 | 22,2 |
| **K** | 100,0 | 0,0 | 0,0 |
| **L** | 80,0 | 0,0 | 20,0 |
| **M** | 100,0 | 0,0 | 0,0 |
| **N** | 66,7 | 0,0 | 33,3 |
| **O** | 22,2 | 22,2 | 55,6 |

**Legend**: A=Inert treatment; B=Acupuncture; C=Back school; D=Cognitive behavioral therapy; E=Education; F=Exercise; G=Heat wrap; H=Manual therapy; I=Muscle relaxant; J=NSAIDs; K=Opioids; L=Paracetamol; M=Physical therapy; N=Steroids; O=Usual care

# Table 3. Presence of leg pain or sciatica

| | FREQUENCIES (%) | | |
|---|---|---|---|
| **TREATMENT** | Yes | No | Not stated |
| **A** | 32,4 | 41,2 | 26,5 |
| **B** | 50,0 | 50,0 | 0,0 |
| **C** | 50,0 | 50,0 | 0,0 |
| **D** | 0,0 | 25,0 | 75,0 |
| **E** | 60,0 | 40,0 | 0,0 |
| **F** | 57,1 | 42,9 | 0,0 |
| **G** | 0,0 | 80,0 | 20,0 |
| **H** | 25,0 | 58,3 | 16,7 |
| **I** | 10,0 | 30,0 | 60,0 |
| **J** | 22,2 | 61,1 | 16,7 |
| **K** | 0,0 | 66,7 | 33,3 |
| **L** | 40,0 | 60,0 | 0,0 |
| **M** | 0,0 | 100,0 | 0,0 |
| **N** | 33,3 | 33,3 | 33,3 |
| **O** | 33,3 | 22,2 | 44,4 |

**Legend**: A=Inert treatment; B=Acupuncture; C=Back school; D=Cognitive behavioral therapy; E=Education; F=Exercise; G=Heat wrap; H=Manual therapy; I=Muscle relaxant; J=NSAIDs; K=Opioids; L=Paracetamol; M=Physical therapy; N=Steroids; O=Usual care

*Presence of leg pain or sciatica was reported in 15 studies out of 46 (31%) of which 6 were not included in quantitative analysis (qualitative analysis).
*Leg pain or sciatica is present in 32% (median, IQR 5-45%) of studies whereas 17% of studies did not report information (median, 0-33%).

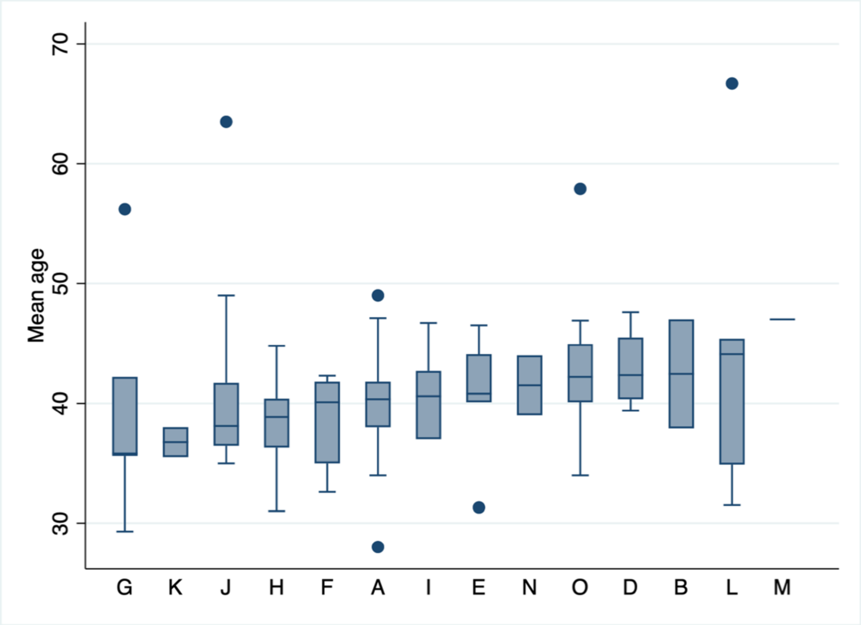


Median age ranged from 35 to 48 years old with overlapping of 25-75 percentiles across interventions as already known by the Global Burden of Disease. [21]

Figure 1. Mean age

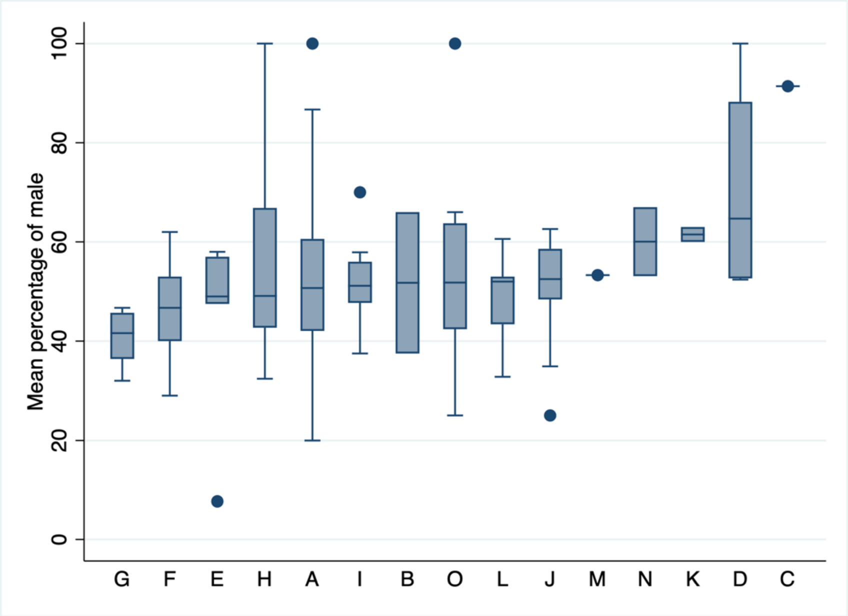


Median percentage of male ranged from 42% to 90% with overlapping of 25-75 percentiles across interventions.*

Figure 2. Percentage of male participants

*Five studies did not report geder; outliers referts to 2 studies with a 100% male; however, these trials did not report outcome data and were not included in quantitative analysis (qualitative analysis). Excluding them, male and female can be equally distributed across interventions.

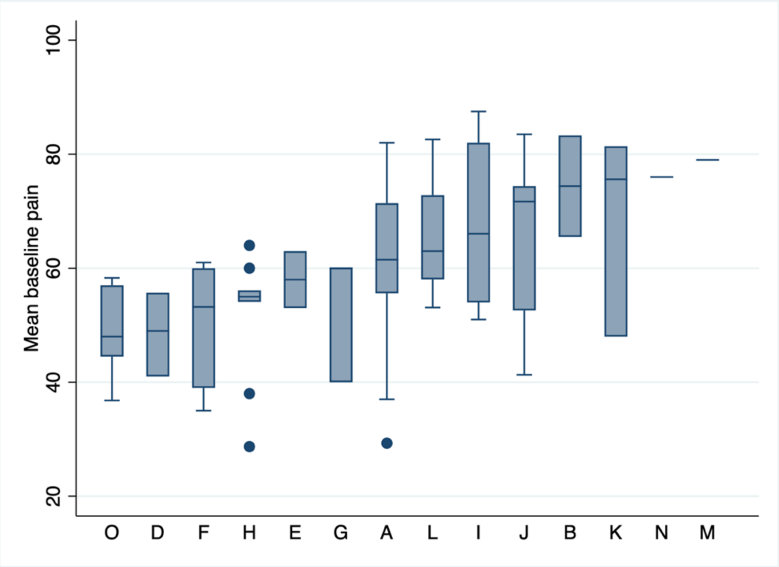


Median baseline pain ranged from 37 to 78 with overlapping of 25-75 percentiles across interventions.

# Figure 3. Baseline severity (pain)

*A: 1 trial out of 34 had an outlier mean baseline value of 29.3, however this trail did not report outcome data and was not included in quantitative analysis (qualitative analysis);
**H: 1 trial out of 12 had an outlier mean baseline value of 28.7, however this trail did not report outcome data and was not included in quantitative analysis (qualitative analysis)
Severity of pain based on adapted scale 0-100

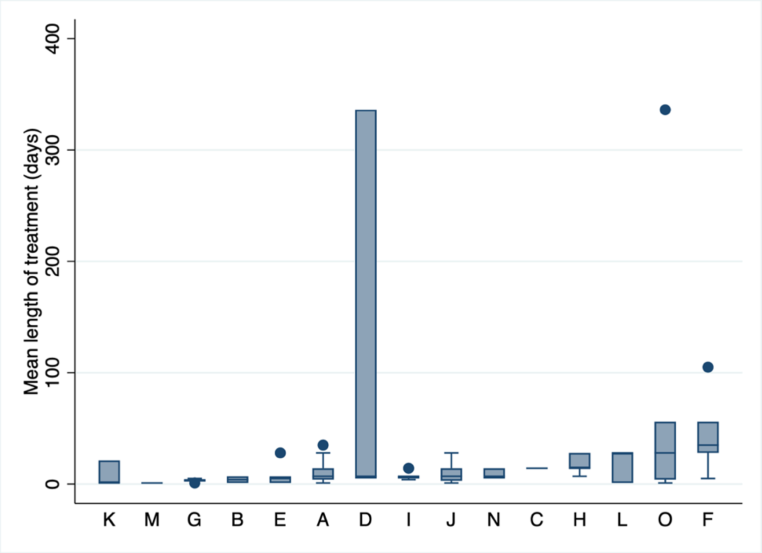


Median length of treatment ranged from 1 to 40 days with overlapping of 25-75 percentiles across interventions.

# Figure 4. Length of treatment

*D: 1 trial out of 4 had an outlier mean length of treatment of 336 days, however this trail did not report outcome data and was not included in quantitative analysis (qualitative analysis); **O: 1 trial had out of 9 an outlier median lenght of treatment of 336 days, however this trail did not report outcome data and was not included in quantitative analysis (qualitative analysis)

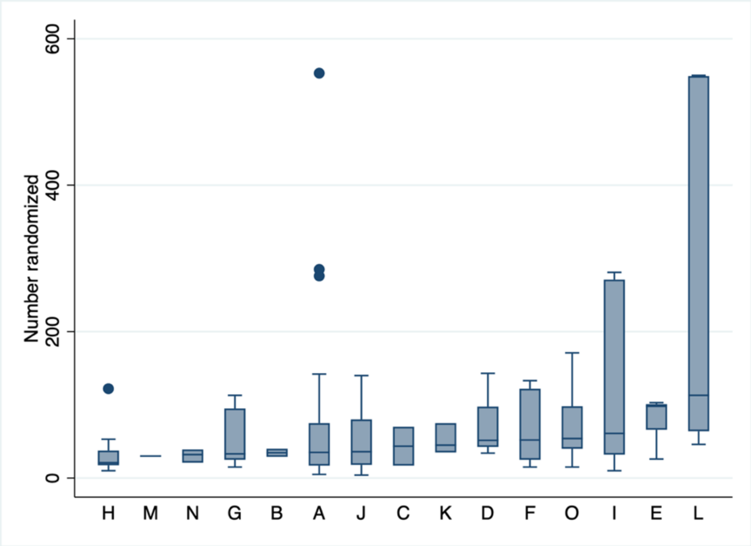


Median number of randomized ranged from 30 to 90 with overlapping of 25-75 percentiles across interventions.

# Figure 5. Number of randomized

*L: 1 trial out of 4 had an outlier number of randomized of 550, which represents less than 5% of the overall sample. However, we judged this reason insufficient to affect transitivity across interventions.
**A: 1 trial out of 34 had an outlier number of randomized of 545, which represents less than 5% of the overall sample. However, we judged this reason insufficient to affect transitivity across interventions.

**Legend**: A=Inert treatment; B=Acupuncture; C=Back school; D=Cognitive behavioral therapy; E=Education; F=Exercise; G=Heat wrap; H=Manual therapy; I=Muscle relaxant; J=NSAIDs; K=Opioids; L=Paracetamol; M=Physical therapy; N=Steroids; O=Usual care

# Table 4. Pshycological assessment

Overall, 10 RCTs (22%) reported a psychological assessment as baseline characteristics of samples. We found heteroegeneity and poor reporting in outcome measurements with missing data; thus, we did not explore the heterogeneity across all included studies. We reported the phsychological assessment in a table format.

**PSYCHOLOGICAL ASSESSMENT**

| ID | Author | Category of Intervention | Scores at baseline | Mean (SD) |
|---|---|---|---|---|
| **4** | Bertalanffy 2005 | Physical therapy | Anxiety score[a] | 82,0 (8,0) |
| **4** | Bertalanffy 2005 | Inert treatment | Anxiety score[a] | 85,0 (6,0) |
| **6** | Cherkin 1996 | Education | Worry about pain[b] | 6,0 |
| **6** | Cherkin 1996 | Education | Worry about pain[b] | 6,0 |
| **6** | Cherkin 1996 | Usual care | Worry about pain[b] | 5,7 |
| **12** | Faas 1995 | Usual care | NHP (emotion)[c] | 7,4 |
| **12** | Faas 1995 | Inert treatment | NHP (emotion)[c] | 7,2 |
| **12** | Faas 1995 | Exercise | NHP (emotion)[c] | 7,7 |
| **16** | Hindle 1972 | Muscle relaxant | Anxiety and tension[d] | 2,6 |
| **16** | Hindle 1972 | Inert treatment | Anxiety and tension[d] | 2,2 |
| **17** | Jellema 2005 | Cognitive behavioral therapy | FABQpa[e]<br>CSQ[f] | 14,3 (5,6)<br>10,3 (6,6) |
| **17** | Jellema 2005 | Usual care | FABQpa[e]<br>CSQ[f] | 15,3 (5,2)<br>11,2 (6,9) |
| **23** | Miki 2018 | Paracetamol | PCS[g] | 24,5 (1,5) |
| **23** | Miki 2018 | NSAIDs | PCS[g] | 30,7 (1,7) |
| **32** | Schneider 2015 | Manual therapy | FABQ[h] | 32,7 (15,3) |
| **32** | Schneider 2015 | Manual therapy | FABQ[h] | 33,0 (18,6) |
| **32** | Schneider 2015 | Usual care | FABQ[h] | 33,0 (17,8) |
| **36** | Storheim 2003 | Exercise | FABQpa[e]<br>FABQw[i] | 13,3 (5,2)<br>25,9 (9,7) |
| **36** | Storheim 2003 | Cognitive behavioral therapy | FABQpa[e]<br>FABQw[i] | 14,1 (4,4)<br>26,7 (9,1) |
| **36** | Storheim 2003 | Usual care | FABQpa[e]<br>FABQw[i] | 14,6 (3,8)<br>29,1 (8,2) |
| **40** | Traeger 2019 | Education | PCS[g]<br>DASS[j] | 18,3 (12)<br>4,1 (3,7) |
| **40** | Traeger 2019 | Inert treatment | PCS[g]<br>DASS[j] | 19,9 (11,2)<br>5,1 (5) |
| **45** | Williams 2014 | Paracetamol | Feelings of depression[k] | 3,2 (2,9) |
| **45** | Williams 2014 | Paracetamol | Feelings of depression[k] | 3,1 (2,9) |
| **45** | Williams 2014 | Inert treatment | Feelings of depression[k] | 3,1 (2,9) |

[a] Visual analogue scale from 0 (no anxiety) to 100 (highest anxiety)
[b] Numeric rating scale from 0 (no worry) to 10 (extremely worried)
[c] NHP: Nottingham Health Profile – emotional reactions domains from 0 (good subjective health status) to 100 (poor subjective health status)
[d] Four step severity rating scale from 1 (none) to 4 (severe)
[e] FABQpa: Fear-avoidance belief questionnaire - four item physical activity subscale from 0 to 24, with higher score indicating more strongly held fear avoidance beliefs
[f] CSQ: Coping strategies questionnaire - six item subscale from 0 to 36, with higher scores indicating greater use of coping strategies
[g] PCS: Pain catastrophizing scale from 0 to 52, with higher scores indicating higher levels of catastrophizing
[h] FABQ: Fear-avoidance belief questionnaire from 0 to 96, with higher score indicating more strongly held fear avoidance beliefs
[i] FABQw: Fear-avoidance belief questionnaire - seven item physical activity subscale from 0 to 42, with higher score indicating more strongly held fear avoidance beliefs
[j] DASS: Depression severity scale of Depression, Anxiety and Stress Scale with range from 0 (no depressive symptoms) to 42 (high depressive symptoms)
[k] Feelings of depression from 0 (not at all) to 10 (extremely).

# Assessment of transitivity by head-to-head comparisons

## Table 5. Stage of LBP

| | FREQUENCIES (%) | | |
|---|---|---|---|
| **COMPARISONS** | Acute | Subacute* | Mixed (acute and subacute) |
| **AB** | 100,0 | 0,0 | 0,0 |
| **AC** | 50,0 | 0,0 | 50,0 |
| **AE** | 100,0 | 0,0 | 0,0 |
| **AF** | 100,0 | 0,0 | 0,0 |
| **AG** | 0,0 | 0,0 | 100,0 |
| **AH** | 85,7 | 0,0 | 14,3 |
| **AI** | 100,0 | 0,0 | 0,0 |
| **AJ** | 72,7 | 0,0 | 27,3 |
| **AL** | 50,0 | 0,0 | 50,0 |
| **AM** | 100,0 | 0,0 | 0,0 |
| **AN** | 100,0 | 0,0 | 0,0 |
| **AO** | 100,0 | 0,0 | 0,0 |
| **BJ** | 100,0 | 0,0 | 0,0 |
| **CH** | 100,0 | 0,0 | 0,0 |
| **CJ** | 100,0 | 0,0 | 0,0 |
| **DF** | 0,0 | **100,0** | 0,0 |
| **DO** | 0,0 | **50,0** | 50,0 |
| **EF** | 0,0 | 0,0 | 100,0 |
| **EG** | 0,0 | 0,0 | 100,0 |
| **EH** | 0,0 | 0,0 | 100,0 |
| **EO** | 0,0 | 0,0 | 100,0 |
| **FG** | 0,0 | 0,0 | 100,0 |
| **FH** | 0,0 | 0,0 | 100,0 |
| **FO** | 66,7 | **33,3** | 0,0 |
| **GJ** | 0,0 | 0,0 | 100,0 |
| **GL** | 0,0 | 0,0 | 100,0 |
| **GO** | 0,0 | 0,0 | 100,0 |
| **HJ** | 100,0 | 0,0 | 0,0 |
| **HO** | 0,0 | 0,0 | 100,0 |
| **JK** | 100,0 | 0,0 | 0,0 |
| **JL** | 66,7 | 0,0 | 33,3 |
| **JN** | 0,0 | 0,0 | 100,0 |
| **KK** | 100,0 | 0,0 | 0,0 |

**Legend**: A=Inert treatment; B=Acupuncture; C=Back school; D=Cognitive behavioral therapy; E=Education; F=Exercise; G=Heat wrap; H=Manual therapy; I=Muscle relaxant; J=NSAIDs; K=Opioids; L=Paracetamol; M=Physical therapy; N=Steroids; O=Usual care

*only 3 comparisons investigated subacute population:
DO: 50% was due to 2 studies (Lindstrom 1995 and Storheim 2003)
DF: 100% was due to 1 study (Storheim 2003)
FO: 33% was due to 1 study (Storheim 2003)
Generally, covariates were equally distributed acrosss comparisons except for a very little percentage of comparisons (0.09%) represented by subacute population.
Moreover, these comparisons are present only in medium and long-terms of follow-ups:

- For both pain and disability at medium term no NMA was performed due to a disconnected network;
- For pain at long term, subacute population is present in 1 out of 4 head-to head comparisons;
- For disaibility at long term, subacute population is present in 3 out 5 head-to head comparisons.

Moreover, there is no consensus on the time-contingent traditional classification (acute, subacute, chronic) because this classificiation does not adequately reflect the prognostically highly important process of chronification [22].
For all these reasons, stage of pain can not be considered a potential effect modifier.

# Table 6. Presence of leg pain or sciatica

| | FREQUENCIES (%) | | |
|---|---|---|---|
| **COMPARISONS** | Yes* | No | Not stated |
| **AB** | 0,0 | 100,0 | 0,0 |
| **AC** | 50,0 | 50,0 | 0,0 |
| **AE** | **100,0** | 0,0 | 0,0 |
| **AF** | **100,0** | 0,0 | 0,0 |
| **AG** | 0,0 | 100,0 | 0,0 |
| **AH** | 28,6 | 42,9 | 28,6 |
| **AI** | 12,5 | 25,0 | 62,5 |
| **AJ** | 27,3 | 54,6 | 18,2 |
| **AL** | 50,0 | 50,0 | 0,0 |
| **AM** | 0,0 | 100,0 | 0,0 |
| **AN** | 50,0 | 0,0 | 50,0 |
| **AO** | **100,0** | 0,0 | 0,0 |
| **BJ** | **100,0** | 0,0 | 0,0 |
| **CH** | 0,0 | 100,0 | 0,0 |
| **CJ** | 0,0 | 100,0 | 0,0 |
| **DF** | 0,0 | 100,0 | 0,0 |
| **DO** | 0,0 | 25,0 | 75,0 |
| **EF** | 0,0 | 100,0 | 0,0 |
| **EG** | 0,0 | 100,0 | 0,0 |
| **EH** | 0,0 | 100,0 | 0,0 |
| **EO** | **100,0** | 0,0 | 0,0 |
| **FG** | 0,0 | 100,0 | 0,0 |
| **FH** | 50,0 | 50,0 | 0,0 |
| **FO** | **66,7** | 33,3 | 0,0 |
| **GJ** | 0,0 | 100,0 | 0,0 |
| **GL** | 0,0 | 100,0 | 0,0 |
| **GO** | 0,0 | 0,0 | 100,0 |
| **HJ** | 25,0 | 50,0 | 25,0 |
| **HO** | 0,0 | 100,0 | 0,0 |
| **JK** | 0,0 | 66,7 | 33,3 |
| **JL** | 0,0 | 100,0 | 0,0 |
| **JN** | 0,0 | 100,0 | 0,0 |
| **KK** | 0,0 | 100,0 | 0,0 |

**Legend**: A=Inert treatment; B=Acupuncture; C=Back school; D=Cognitive behavioral therapy; E=Education; F=Exercise; G=Heat wrap; H=Manual therapy; I=Muscle relaxant; J=NSAIDs; K=Opioids; L=Paracetamol; M=Physical therapy; N=Steroids; O=Usual care

Presence of leg pain or sciatica was reported in 15 studies out of 46 (31%) of which 6 were not included in quantitative analysis.

*AE: 1 study
*AF: 2 studies, of which 1 was not included in quantitative analysis (qualitative analysis).
*EO: 1 study not included in quantitative analysis (qualitative analysis).
*BJ: 1 study
*AO: 1 study not included in quantitative analysis (qualitative analysis).
*FO: 2 studies of which 1 was not included in quantitative analysis (qualitative analysis).
Overall, a very little percentage of leg pain or sciatica (0.09%) impact on global assessment.

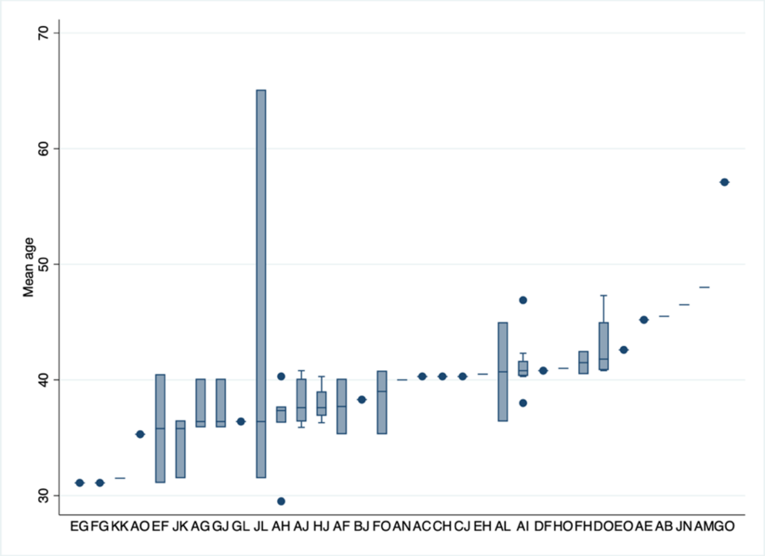


Median of mean age ranged from 32 to 57 years old as already known by the Global Burden of Disease [21]

## Figure 6. Mean age

*JL: 1 out of 3 trials has a mean age of 65.1

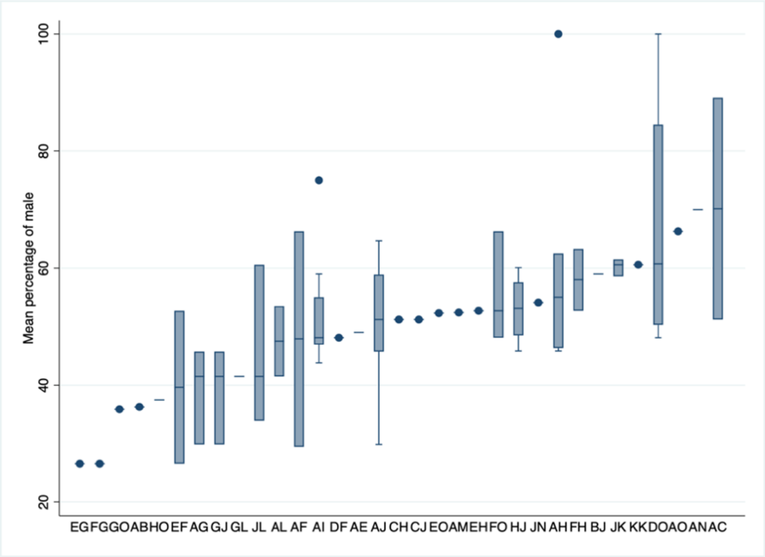


Median percentage of male ranged from 27 to 70 percent with overlapping of 25-75 percentiles across comparisons.

## Figure 7. Percentage of male participants

*Five studies did not report gender

**AH and DO: outliers refer to 2 studies with a 100% male; however, these trials did not report outcome data and were not included in quantitative analysis (qualitative analysis). Excluding them, male and female can be equally distributed across interventions.

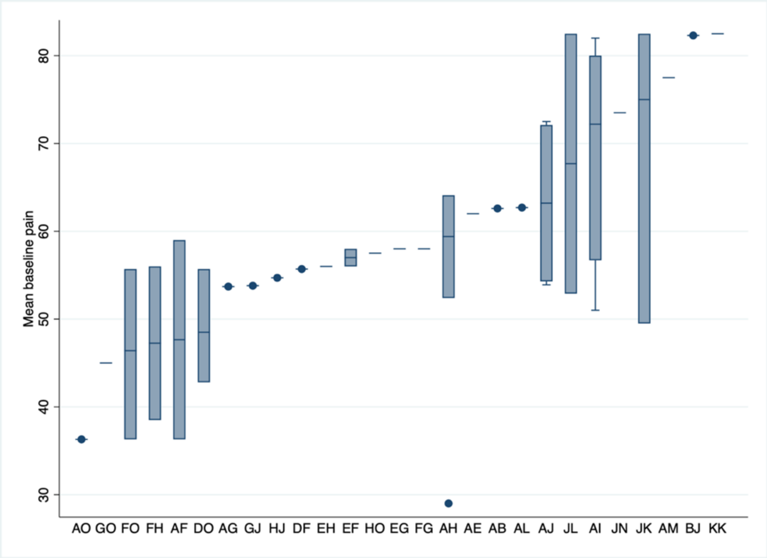


Median baseline pain ranged from 37 to 82 with overlapping of 25-75 percentiles across comparisons.

## Figure 8. Baseline severity (pain)

*AH: 1 trial had an outlier mean baseline value of 29, however this trail did not report outcome data and was not included in quantitative analysis (qualitative analysis)

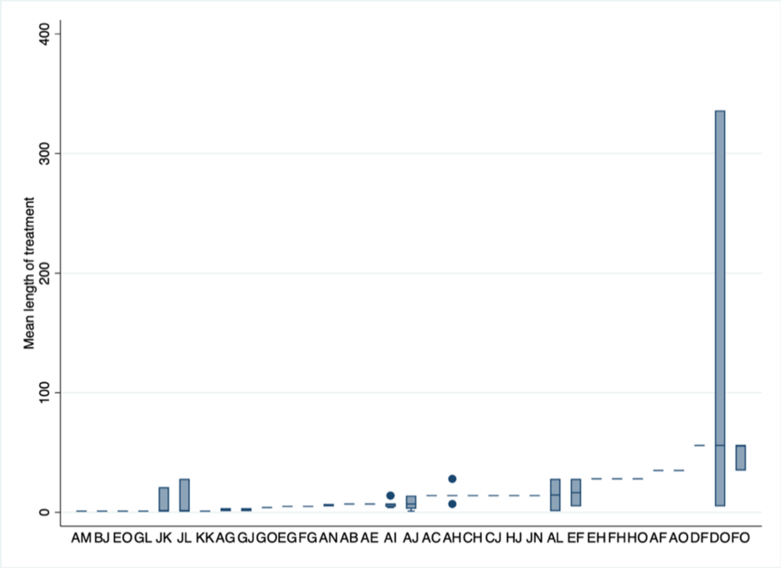


Median length of treatment ranged from 1 to 40 days with overlapping of 25-75 percentiles across comparisons.

## Figure 9. Length of treatment

*DO: 1 trial had an outlier mean lenght of treatment of 336 days, however this trail did not report outcome data and was not included in quantitative analysis (qualitative analysis)

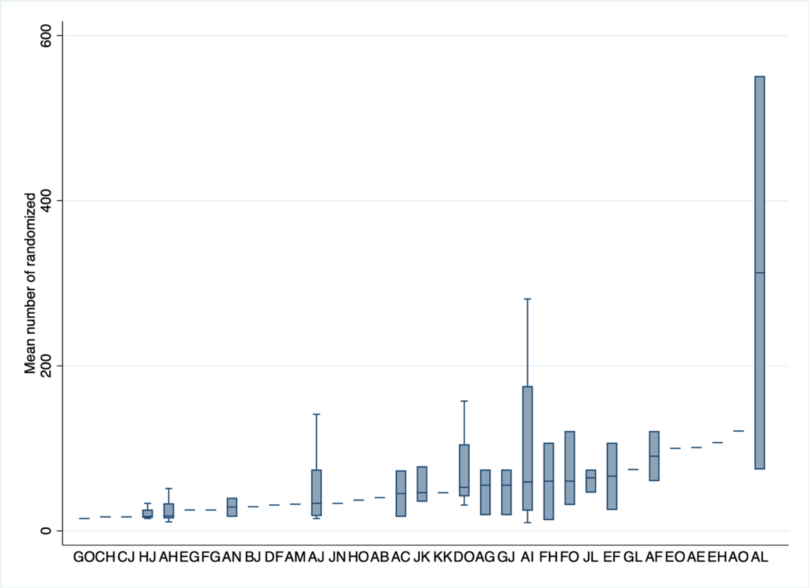


# Figure 10. Number of randomized

*AL: 1 trial had an outlier number of randomized of 550, which represents less than 5% of the overall sample. However, we judged this reason insufficient to prejudice transitivity across interventions.

**Legend**: A=Inert treatment; B=Acupuncture; C=Back school; D=Cognitive behavioral therapy; E=Education; F=Exercise; G=Heat wrap; H=Manual therapy; I=Muscle relaxant; J=NSAIDs; K=Opioids; L=Paracetamol; M=Physical therapy; N=Steroids; O=Usual care

# Supplement F. Risk of Bias

## Figure 1. Aggregate Cochrane Risk-of-bias appraisal results

Risk of bias appraisal.[23]

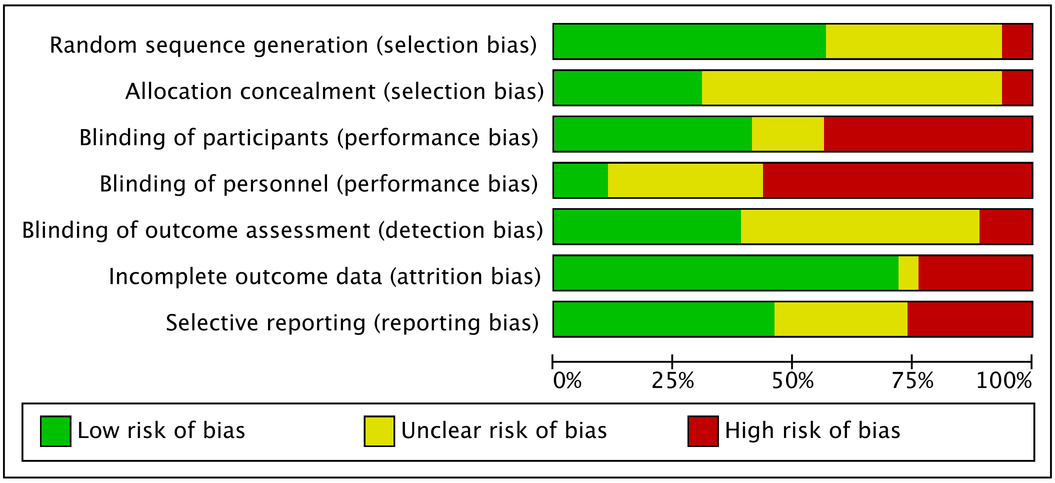

# Table 1. Cochrane Risk-of-bias global judgement

| Author, year | Random sequence generation | Allocation concealment | Blinding of participants | Blinding of personnel/ care providers (performance bias) | Blinding of outcome assessment | Incomplete outcome data | Selective Reporting | FINAL JUDGEMENT |
|---|---|---|---|---|---|---|---|---|
| **Amlie 1987** | unclear | unclear | low | unclear | unclear | low | low | unclear |
| **Bergquist-Ullman 1977** | low | unclear | high | high | unclear | high | low | high |
| **Berry 1988** | unclear | unclear | unclear | unclear | unclear | low | low | unclear |
| **Bertalanffy 2005** | low | low | low | high | low | low | low | low |
| **Casale 1988** | unclear | unclear | low | unclear | unclear | low | high | unclear |
| **Cherkin 1996** | high | unclear | high | high | low | low | unclear | unclear |
| **Cherkin 1998** | unclear | low | high | high | low | low | unclear | low |
| **Dapas 1985** | unclear | unclear | low | unclear | unclear | high | high | high |
| **Dreiser 2003** | low | low | low | unclear | unclear | low | low | unclear |
| **Eken 2014** | low | low | low | low | unclear | low | low | unclear |
| **Eskin 2014** | low | unclear | unclear | low | low | low | low | unclear |
| **Faas 1995** | high | unclear | high | high | high | low | low | high |
| **Goldie 1968** | unclear | unclear | low | low | unclear | low | low | unclear |
| **Haimovic 1986** | low | unclear | low | unclear | unclear | high | unclear | high |
| **Hasagawa 2014** | low | unclear | low | high | low | low | low | unclear |
| **Hindle 1972** | low | high | unclear | unclear | unclear | low | high | high |
| **Jellema 2005** | low | unclear | high | high | unclear | low | unclear | unclear |
| **Ketenci 2005** | unclear | unclear | low | unclear | unclear | low | low | unclear |
| **Kettenmann 2007** | high | high | high | unclear | high | high | unclear | high |
| **Lindstrom 1995** | unclear | unclear | high | unclear | unclear | low | high | unclear |
| **Malmivaara 1995** | low | low | high | high | low | low | low | low |
| **Mayer 2005** | low | unclear | high | high | unclear | low | high | unclear |
| **Miki 2018** | low | unclear | high | high | unclear | high | high | high |
| **Nadler 2002** | unclear | unclear | high | high | unclear | low | unclear | unclear |
| **Nadler 2003b** | unclear | unclear | high | high | unclear | low | unclear | unclear |
| **Nadler 2003a** | unclear | unclear | high | high | unclear | high | unclear | high |
| **Postacchini 1988** | unclear | unclear | unclear | unclear | unclear | unclear | high | unclear |
| **Ralph 2008** | unclear | unclear | unclear | unclear | unclear | low | high | unclear |

| | | | | | | | | |
|---|---|---|---|---|---|---|---|---|
| **Sae-Jung 2016** | low | low | unclear | high | high | low | low | high |
| **Santilli 2006** | low | low | low | high | low | low | unclear | low |
| **Schenk 2003** | low | unclear | high | high | high | low | unclear | high |
| **Schneider 2015** | low | low | high | high | low | low | high | low |
| **Seferlis 1998** | unclear | unclear | high | high | unclear | high | low | high |
| **Serfer 2010** | low | unclear | low | low | high | low | low | high |
| **Shin 2013** | low | low | high | high | low | low | low | low |
| **Storheim 2003** | low | low | high | high | low | high | low | high |
| **Suni 2006** | low | unclear | high | high | low | unclear | unclear | unclear |
| **Szpalski 1994** | unclear | unclear | unclear | unclear | unclear | low | low | unclear |
| **Takamoto 2015** | low | unclear | high | high | low | high | high | high |
| **Traeger 2019** | low | low | low | high | low | low | low | low |
| **Tuzun 2003** | low | low | low | unclear | low | low | low | low |
| **Veenema 2000** | unclear | high | low | high | low | low | unclear | high |
| **Videman 1984** | unclear | unclear | low | unclear | unclear | low | unclear | unclear |
| **Von Heymann 2013** | low | low | low | high | low | high | high | high |
| **Williams 2014** | low | low | low | low | low | low | low | low |
| **Younes 2017** | low | unclear | low | high | low | high | high | high |

# Supplement G. Network Plots

## Figure 1. Network Plot- Pain outcome

**Note:** The size of the nodes is proportional to the number of studies evaluating each intervention, and the thickness of the edges is proportional to the precision (the inverse of the variance) of each direct comparison.

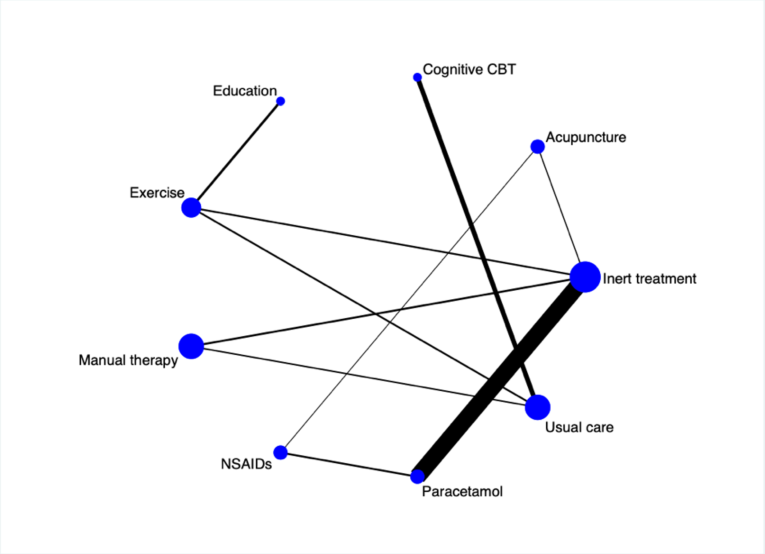


Figure 1a. Network for pain outcome at 1 month of FU

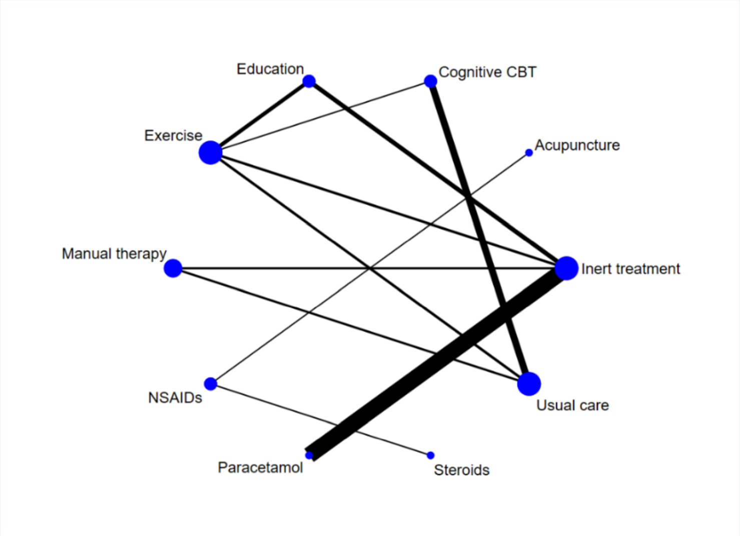


Figure 1b. Network for pain outcome at 3-6 months of FU

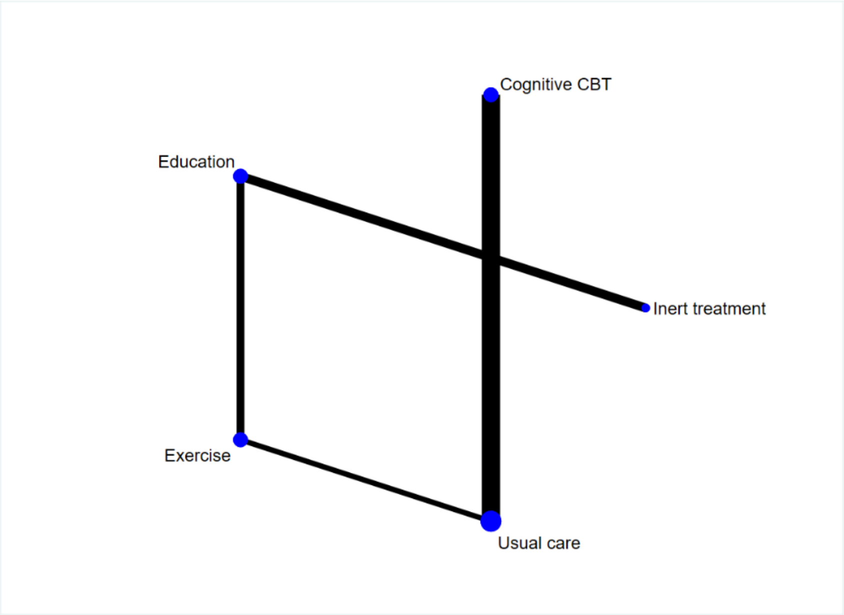


Figure 1c. Network for pain outcome at 12 months of FU

# Figure 2. Network Plot- Disability outcome

**Note:** The size of the nodes is proportional to the number of studies evaluating each intervention, and the thickness of the edges is proportional to the precision (the inverse of the variance) of each direct comparison.

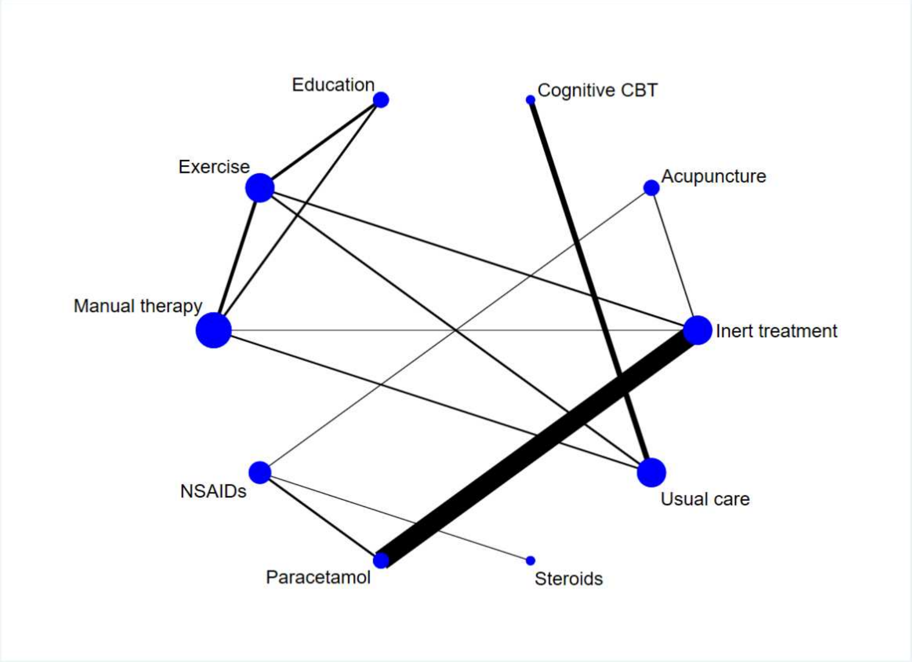


Figure 2a. Network for disability outcome at 1 month of FU

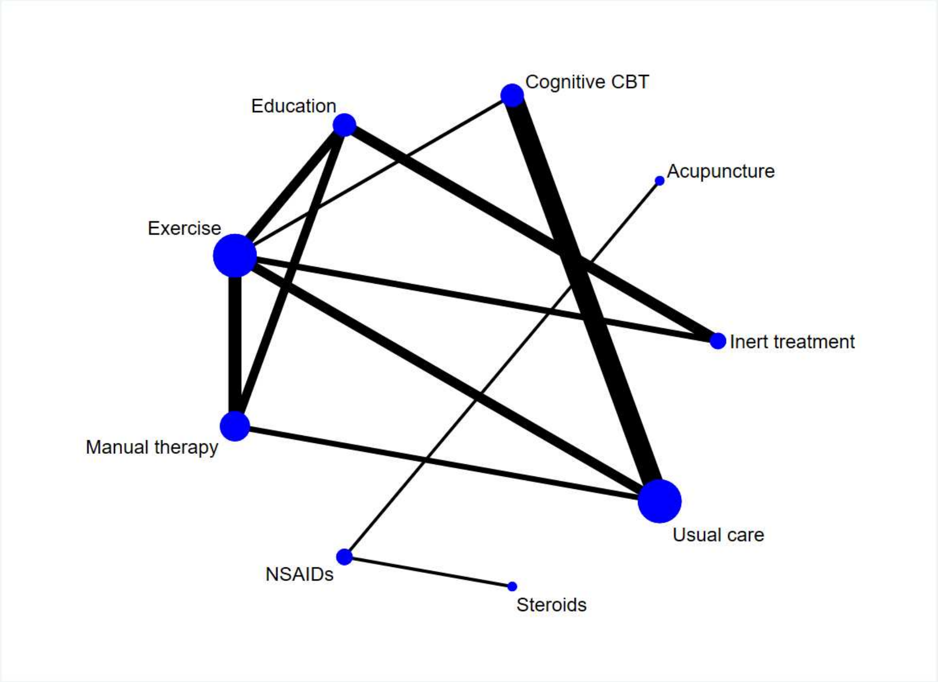


Figure 2b. Network for disability outcome at 3-6 months of FU

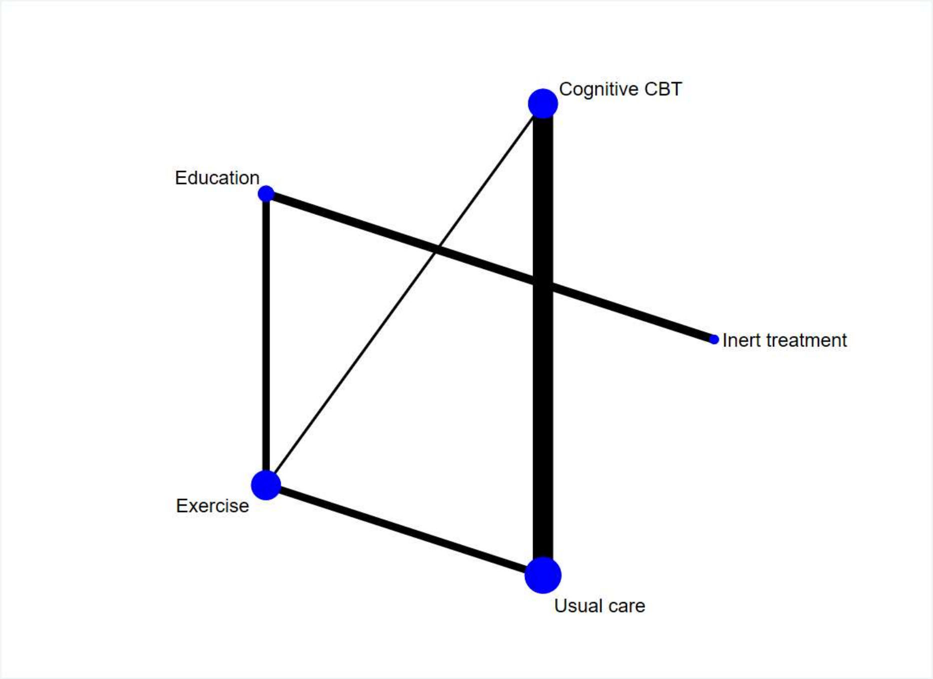


Figure 2c. Network for disability outcome at 12 months of FU

# Supplement H. Assessment of pairwise Meta-Analyses

## Pairwise meta-analyses –Pain Outcome

## Table 1. Pairwise meta-analyses at 1 week of FU for pain

| | Comparison | Number of studies | Effect size | Lower limit 95% | Upper limit 95% | Heterogeneity ($I^2$) | P value |
|---|---|---|---|---|---|---|---|
| 1 | Muscle relaxants vs Inert treatment | 4 | -1.06 | -1.89 | -0.24 | 91.1% | 0.0000 |
| 2 | Physical therapy vs Inert treatment | 1 | -2.85 | -3.57 | -2.14 | Na | Na |
| 3 | NSAIDs vs Inert treatment | 3 | -0.84 | -1.15 | -0.53 | 54.2% | 0.112 |
| 4 | Opioid vs NSAIDs | 2 | -0.43 | -0.71 | -0.14 | 20.3% | 0.263 |
| 5 | Paracetamol vs NSAIDs | 2 | -0.21 | -0.62 | 0.20 | 56.9% | 0.128 |
| 6 | Paracetamol vs Opioid | 1 | 0.18 | -0.24 | 0.59 | Na | Na |
| 7 | Acupuncture vs Inert treatment | 1 | -0.30 | -0.74 | 0.14 | Na | Na |
| 8 | Exercise vs Education | 1 | -0.90 | -1.47 | -0.33 | Na | Na |
| 9 | Heat wrap vs Education | 1 | -1.03 | -1.60 | -0.46 | Na | Na |
| 10 | Heat wrap vs Exercise | 1 | -0.13 | -0.68 | 0.43 | Na | Na |
| 11 | Heat wrap vs Inert treatment | 1 | -4.77 | -5.72 | -3.81 | Na | Na |
| 12 | Manual therapy vs Inert treatment | 2 | -1.20 | -2.59 | 0.19 | 91.1% | 0.000 |
| 13 | Manual therapy vs Exercise | 1 | 1.12 | 0.25 | 1.99 | Na | Na |
| 14 | NSAIDs vs Acupuncture | 1 | -0.58 | -1.11 | -0.06 | Na | Na |
| 15 | Education vs Inert treatment | 1 | 0.04 | -0.23 | 0.32 | Na | Na |
| 16 | NSAIDs vs Manual therapy | 1 | 0.67 | 0.20 | 1.13 | Na | Na |
| 17 | Paracetamol vs Inert treatment | 1 | 0.04 | -0.08 | 0.16 | Na | Na |

## Table 2. Pairwise meta-analyses at 1 month of FU for pain

| | Comparison | Number of studies | Effect size | Lower limit 95% | Upper limit 95% | Heterogeneity ($I^2$) | P value |
|---|---|---|---|---|---|---|---|
| 1 | Exercise vs Education | 1 | -0.84 | -1.14 | -0.53 | Na | Na |
| 2 | Acupuncture vs Inert treatment | 1 | -0.63 | -1.08 | -0.18 | Na | Na |
| 3 | Usual care vs Cognitive CBT | 1 | 0.04 | -0.18 | 0.26 | Na | Na |

| | | | | | | | |
|---|---|---|---|---|---|---|---|
| 4 | Exercise vs Inert treatment | 1 | 0.00 | -0.36 | 0.36 | Na | Na |
| 5 | Paracetamol vs NSAIDs | 1 | -0.08 | -0.43 | 0.27 | Na | Na |
| 6 | Steroids vs NSAIDs | 1 | -1.51 | -2.06 | -0.95 | Na | Na |
| 7 | Manual therapy vs Inert treatment | 2 | -0.86 | -1.45 | -0.27 | 59.7% | 0.115 |
| 8 | Usual care vs Manual therapy | 2 | 0.61 | -0.15 | 1.37 | 72.6% | 0.056 |
| 9 | Usual care vs Exercise | 1 | 0.00 | -0.36 | 0.36 | Na | Na |
| 10 | NSAIDs vs Acupuncture | 1 | -0.55 | -1.07 | -0.02 | Na | Na |
| 11 | Paracetamol vs Inert treatment | 1 | 0.00 | -0.12 | 0.12 | Na | Na |

## Table 3. Pairwise meta-analyses at 3-6 months of FU for pain

| | Comparison | Number of studies | Effect size | Lower limit 95% | Upper limit 95% | Heterogeneity ($I^2$) | P value |
|---|---|---|---|---|---|---|---|
| 1 | Exercise vs Education | 1 | -0.17 | -0.47 | 0.13 | Na | Na |
| 2 | Usual care vs Cognitive CBT | 1 | 0.00 | -0.22 | 0.22 | Na | Na |
| 3 | Manual therapy vs Inert treatment | 1 | -0.80 | -1.20 | -0.40 | Na | Na |
| 4 | Usual care vs Manual therapy | 2 | 0.06 | -0.62 | 0.73 | 66.6% | 0.084 |
| 5 | Usual care vs Exercise | 1 | 0.00 | -0.36 | 0.36 | Na | Na |
| 6 | Exercise vs Cognitive CBT | 1 | -0.47 | -0.97 | 0.03 | Na | Na |
| 7 | Education vs Inert treatment | 1 | -0.08 | -0.36 | 0.19 | Na | Na |
| 8 | Paracetamol vs Inert treatment | 1 | -0.04 | -0.16 | 0.07 | Na | Na |

## Table 4. Pairwise meta-analyses at 12 months of FU for pain

| | Comparison | Number of studies | Effect size | Lower limit 95% | Upper limit 95% | Heterogeneity ($I^2$) | P value |
|---|---|---|---|---|---|---|---|
| 1 | Exercise vs Education | 1 | -0.39 | -0.68 | -0.09 | Na | Na |
| 2 | Usual care vs Cognitive CBT | 2 | 0.09 | -0.40 | 0.58 | 79.3% | 0.028 |
| 3 | Usual care vs Exercise | 1 | 0.00 | -0.36 | 0.36 | Na | Na |
| 4 | Education vs Inert treatment | 1 | -0.30 | -0.58 | -0.03 | Na | Na |

# Pairwise meta-analyses – Disability Outcome

## Table 5. Pairwise meta-analyses at 1 week of FU for disability

| | Comparison | Number of studies | Effect size | Lower limit 95% | Upper limit 95% | Heterogeneity ($I^2$) | P value |
|---|---|---|---|---|---|---|---|
| 1 | NSAIDs-Inert treatment | 2*(3) | -0.432 | -0.664 | -0.199 | 22.3% | 0.000 |
| 2 | Acupuncture- Inert treatment | 1 | -0.385 | -0.828 | 0.057 | Na | 0.088 |
| 3 | Exercise-Education | 1 | -0.291 | -0.842 | 0.260 | Na | 0.300 |
| 4 | Heat Wrap-Education | 1 | -0.414 | -0.967 | 0.140 | Na | 0.143 |
| 5 | Heat Wrap-Exercise | 1 | -0.122 | -0.677 | 0.432 | Na | 0.666 |
| 6 | Paracetamol-NSAIDs | 2 | 0.010 | -0.201 | 0.221 | 0.0% | 0.924 |
| 7 | NSAIDs –Heat Wrap | 1 | -0.512 | -0.780 | -0.244 | Na | 0.000 |
| 8 | Paracetamol–Heat Wrap | 1 | -0.466 | -0.729 | -0.202 | Na | 0.001 |
| 9 | Heat Wrap- Inert treatment | 1 | -0.544 | -0.792 | -0.295 | 0.0% | 0.000 |
| 10 | Muscle Relaxant-Inert treatment | 2*(3) | -0.235 | -0.439 | -0.031 | 70.6% | 0.024 |
| 11 | Manual therapy-Exercise | 1 | 0.772 | -0.063 | 1.606 | Na | 0.070 |
| 12 | NSAIDs – Acupuncture | 1 | -0.732 | -1.265 | -0.199 | Na | 0.007 |
| 13 | Manual therapy-Inert treatment | 2 | -0.660 | -1.099 | -0.221 | 19.6% | 0.003 |
| 14 | Education-Inert treatment | 1 | -0.271 | -0.548 | 0.006 | Na | 0.055 |
| 15 | NSAIDs –Manual Therapy | 1 | 0.793 | 0.327 | 1.260 | Na | 0.001 |
| 16 | Paracetamol-Inert treatment | 1 | -0.092 | -0.210 | 0.026 | Na | 0.126 |

*3 comparisons from 2 studies

## Table 6. Pairwise meta-analyses at 1 month of FU for disability

| | Comparison | Number of studies | Effect size | Lower limit 95% | Upper limit 95% | Heterogeneity ($I^2$) | P value |
|---|---|---|---|---|---|---|---|
| 1 | Usual care – Manual therapy | 1 *(2) | 0.239 | -0.333 | 0.810 | 53.5% | 0.413 |
| 2 | Acupuncture – Inert treatment | 1 | -0.709 | -1.162 | -0.257 | Na | 0.002 |
| 3 | Usual care – Cognitive CBT | 1 | 0.019 | -0.203 | 0.241 | Na | 0.868 |
| 4 | Exercise - Inert treatment | 1 | 0.674 | 0.302 | 1.047 | Na | 0.000 |
| 5 | Paracetamol - NSAIDs | 1 | -0.128 | -0.476 | 0.220 | Na | 0.472 |
| 6 | Steroids - NSAIDs | 1 | -1.215 | -1.747 | -0.682 | Na | 0.000 |

| | | | | | | | |
|---|---|---|---|---|---|---|---|
| 7 | Usual care – Exercise | 1 | 0.000 | -0.358 | 0.358 | Na | 1.000 |
| 8 | NSAIDs Acupuncture | 1 | -0.640 | -1.169 | -0.111 | Na | 0.018 |
| 9 | Manual therapy - Inert treatment | 1 | -0.819 | -1.438 | -0.201 | Na | 0.009 |
| 10 | Paracetamol - Inert treatment | 1 | -0.019 | -0.137 | 0.099 | Na | 0.747 |
| 11 | Exercise - Education | 1 | -0.426 | -0.723 | -0.129 | Na | 0.005 |
| 12 | Manual therapy - Education | 1 | -2.158 | -2.502 | -1.815 | Na | 0.000 |
| 13 | Manual therapy - Exercise | 1 | -1.732 | -2.012 | -1.452 | Na | 0.000 |

*2 comparisons from 1 study

# Table 7. Pairwise meta-analyses at 3-6 months of FU for disability

| | Comparison | Number of studies | Effect size | Lower limit 95% | Upper limit 95% | Heterogeneity ($I^2$) | P value |
|---|---|---|---|---|---|---|---|
| 1 | Usual care – Manual Therapy | 1 *(2) | 0.039 | -0.348 | 0.426 | 0% | 0.844 |
| 2 | Usual care – Cognitive CBT | 2 | 0.212 | -0.333 | 0.757 | 75.4% | 0.446 |
| 3 | Exercise - Inert treatment | 1 | 0.312 | -0.052 | 0.677 | Na | 0.093 |
| 4 | Steroids - NSAIDs | 1 | -0.794 | -1.300 | -0.287 | Na | 0.002 |
| 5 | Usual care - Exercise | 2 | 0.159 | -0.229 | 0.547 | 38.0% | 0.422 |
| 6 | NSAIDs - Acupuncture | 1 | 0.435 | -0.087 | 0.956 | Na | 0.102 |
| 7 | Exercise- Cognitive CBT | 1 | 0.135 | -0.356 | 0.627 | Na | 0.590 |
| 8 | Education - Inert treatment | 1 | -0.096 | -0.372 | 0.180 | Na | 0.496 |
| 9 | Exercise- Education | 1 | -0.052 | -0.347 | 0.243 | Na | 0.731 |
| 10 | Manual therapy - Education | 1 | -0.896 | -1.204 | -0.588 | Na | 0.000 |
| 11 | Manual therapy - Exercise | 1 | -0.844 | -1.099 | -0.590 | Na | 0.000 |

*2 comparisons from 1 study

# Table 8. Pairwise meta-analyses at 12 months of FU for disability

| | Comparison | Number of studies | Effect size | Lower limit 95% | Upper limit 95% | Heterogeneity ($I^2$) | P value |
|---|---|---|---|---|---|---|---|
| 1 | Exercise - Education | 1 | -0.437 | -0.735 | -0.138 | Na | 0.004 |
| 2 | Usual care - Cognitive CBT | 3 | 0.332 | -0.142 | 0.806 | 80.4% | 0.170 |
| 3 | Usual care - Exercise | 2 | 0.185 | -0.249 | 0.619 | 49.5% | 0.403 |
| 4 | Exercise - Cognitive CBT | 1 | 0.086 | -0.405 | 0.577 | Na | 0.732 |
| 5 | Education - Inert treatment | 1 | -0.163 | -0.439 | 0.114 | Na | 0.249 |

# Supplement I. Forest plot of network meta-analysis (network forest)

## Figure 1. Network forest – pain outcome 1 week

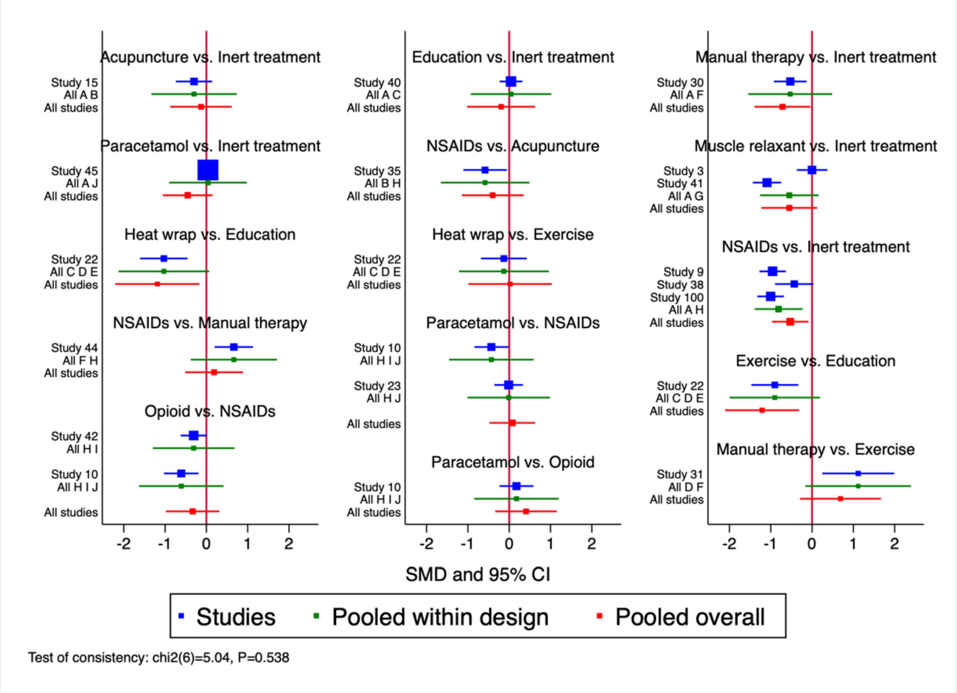


## Figure 2. Network forest – pain outcome 1 month

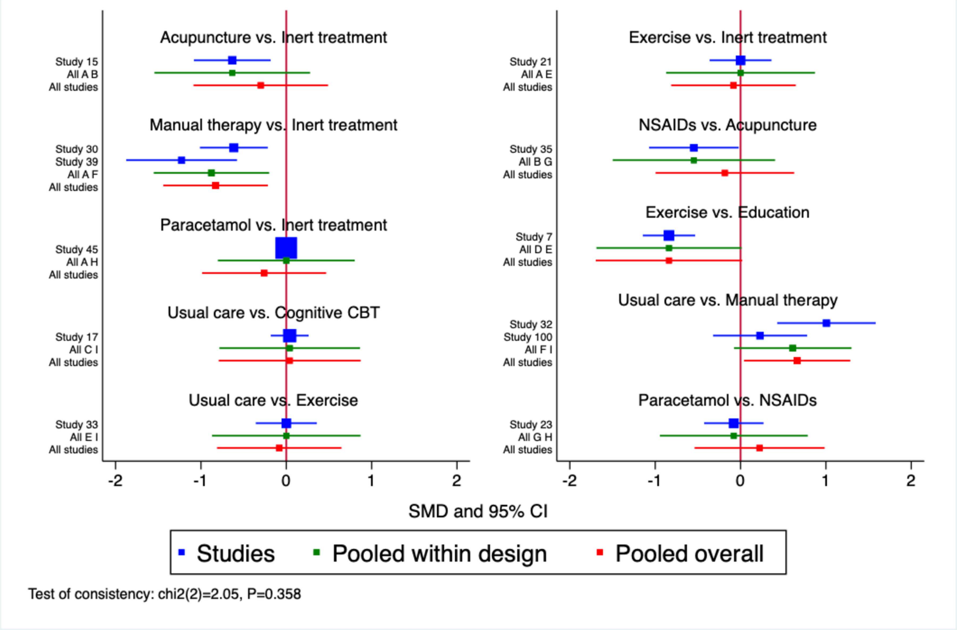

## Figure 3. Network forest – pain outcome 12 months

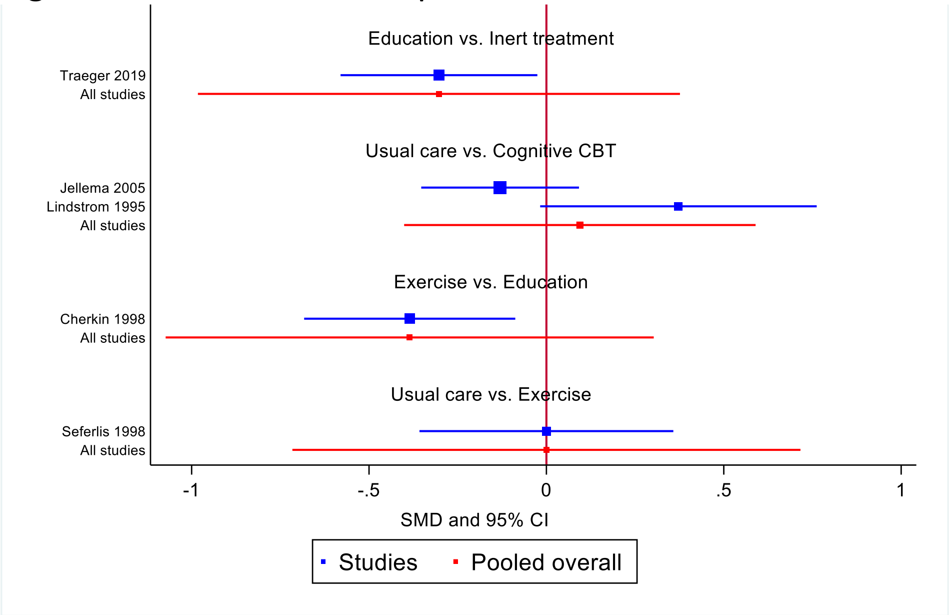


## Figure 4. Network forest – disability outcome 1 week

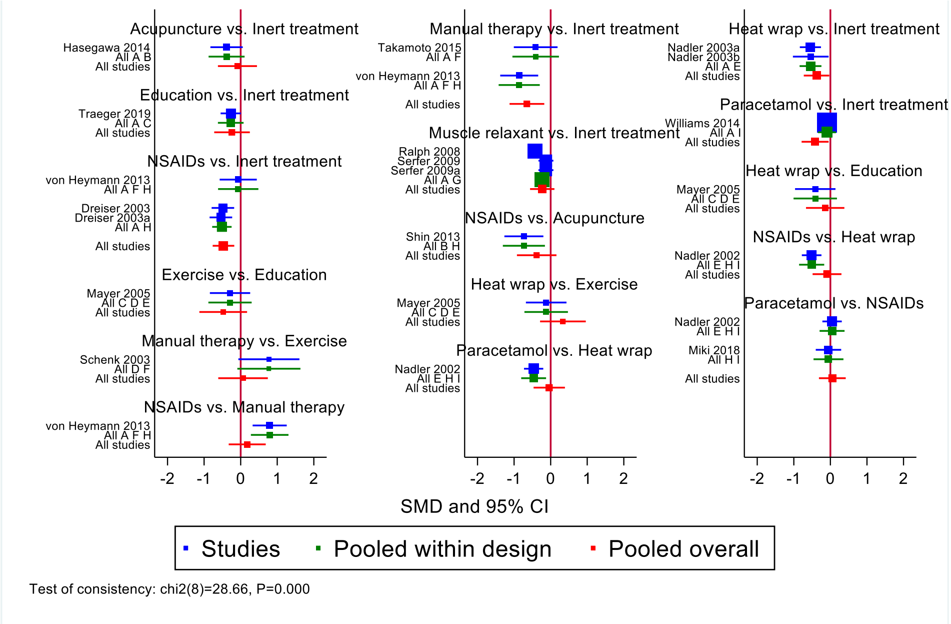

# Figure 5. Network forest – disability outcome 1 month

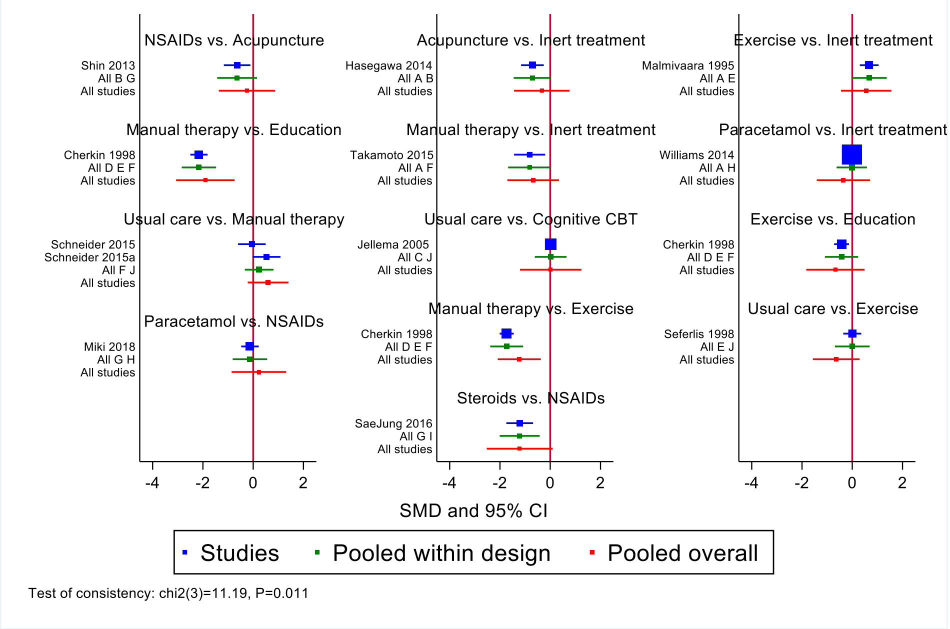


# Figure 6. Network forest – disability outcome 12 months

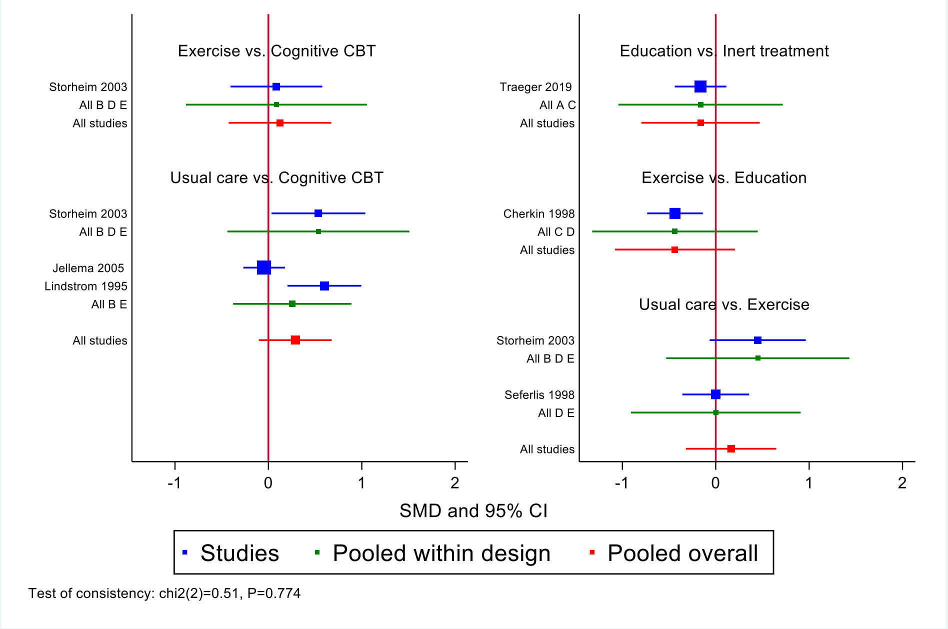

## Supplement J. Incoherence estimation and evaluation

### Table 1. Estimated Global Inconsistency in Networks

| **OUTCOME** | **FOLLOW UP** | **Chi square** | **Prob > chi2** | **tau** |
|---|---|---|---|---|
| PAIN | 1 week | chi2 (7) = 9.48 | Prob > chi2 = 0.5383 | 0.234 |
| | 1 month | chi2 (2) = 2.05 | Prob > chi2 = 0.3583 | 0.169 |
| | 3-6 months | disconnected | | - |
| | 12 months | chi2 (1) = 0.00 | Prob > chi2 = 1** | 0.1 |
| DISABILITY | 1 week | chi2 (8) =28.66 | Prob > chi2 = 0.0004* | - |
| | 1 month | chi2 (3) =11.20 | Prob > chi2 = 0.0107* | - |
| | 3-6 months | disconnected | | - |
| | 12 months | chi2 (2) = 0.51 | Prob > chi2 = 0.7737 | 0.097 |

* Global consistency is tested here using the 'design-by-interaction' test that infers consistency across an entire treatment network, using a chi square test. A p value <0.05 is taken to infer evidence of global inconsistency in the network. [24 25]
**all the evidence about these contrasts comes from the trials which directly compare them

### Table 2. Estimated Local Inconsistency for each pairwise comparison (side splitting) – pain outcome

### Table 2a. Nodesplit pain 1 week

| **Side** | **Direct** | | **Indirect** | | **Difference** | | | **tau** |
|---|---|---|---|---|---|---|---|---|
| | Coef. | Std. Err. | Coef. | Std. Err. | Coef. | Std. Err. | P>z | |
| **Inert treatment - Acupuncture** | -.2987834 | .5246669 | .0931138 | .5981655 | -.3918972 | .7956616 | 0.622 | .4740148 |
| **Inert treatment - Education** | .0432741 | .4689486 | -1079062,00 | .9044266 | 1122337,00 | 1018774,00 | 0.271 | .4473322 |
| **Inert treatment - Manual therapy** | -.5280427 | .5132268 | -.8939374 | .5025075 | .3658947 | .7182726 | 0.610 | .4719181 |
| **Inert treatment - Muscle relaxant** | . | . | . | . | . | . | . | . |

| | | | | | | | | |
|---|---|---|---|---|---|---|---|---|
| **Inert treatment - NSAIDs** | -.8159915 | .2426794 | -.0329156 | .3199731 | -.7830758 | .4018672 | 0.051 | .3754527 |
| **Inert treatment - Paracetamol** | .0384353 | .4065262 | -.8652568 | .3777104 | .9036921 | .5549132 | 0.103 | .4020402 |
| **Acupuncuture - NSAIDs** | -.5837083 | .5448436 | -.1918109 | .5798476 | -.3918974 | .7956619 | 0.622 | .4740148 |
| **Education – Exercise *** | -.9012443 | .5332432 | -2023588,00 | .8680764 | 1122343,00 | 1018776,00 | 0.271 | .4473321 |
| **Education - Heat wrap *** | -1029994,00 | .5348997 | -3274667,00 | 1963983,00 | 2244673,00 | 2037546,00 | 0.271 | .4473318 |
| **Exercise - Heat wrap *** | -.1287492 | .5293618 | 2115939,00 | 1968485,00 | -2244688,00 | 2037552,00 | 0.271 | .4473321 |
| **Exercise - Manual therapy** | 1117072,00 | .6305311 | -.005282 | .8002101 | 1122354,00 | 1018777,00 | 0.271 | .4473321 |
| **Manual therapy - NSAIDs** | .6652757 | .4944677 | -.2694296 | .4841419 | .9347054 | .69202 | 0.177 | .4335961 |
| **NSAIDs - Opiod *** | -.4512816 | .3356582 | .9098231 | 1082583,00 | -1361105,00 | 1133386,00 | 0.230 | .4358473 |

* All the evidence about these contrasts comes from the trials which directly compare them.

# Table 2b. Nodesplit pain 1 month

| **Side** | **Direct** | | **Indirect** | | **Difference** | | | **tau** |
|---|---|---|---|---|---|---|---|---|
| | Coef. | Std. Err. | Coef. | Std. Err. | Coef. | Std. Err. | P>z | |
| **Inert treatment - Acupuncture** | -.6327764 | .3567964 | .6254979 | .5752867 | -1.258.274 | .6769479 | 0.063 | .273273 |
| **Inert treatment - Exercise** | -4.80e-12 | .5233844 | -.2740767 | .7685576 | .2740767 | .9298451 | 0.768 | .4896684 |
| **Inert treatment - Manual therapy** | -.8871542 | .3955099 | -.613068 | .8416375 | -.2740862 | .9298405 | 0.768 | .4896674 |
| **Inert treatment - Paracetamol** | -2.90e-12 | .2798297 | -1.258.269 | .6164035 | 1.258.269 | .6769475 | 0.063 | .273273 |
| **Acupuncture - NSAIDs** | -.5466608 | .3826874 | .7116145 | .5583996 | -1.258.275 | .6769489 | 0.063 | .2732733 |
| **Cognitive CBT - Usual care *** | .0399034 | .4245035 | -.3263798 | 6.354.628 | .3662832 | 6.354.629 | 1.000 | .4090962 |
| **Education - Exercise *** | -.8383118 | .4379943 | -.4467205 | 6.328.197 | -.3915912 | 6.328.198 | 1.000 | .4090963 |
| **Exercise - Usual care** | -2.29e-08 | .5225983 | -.2740773 | .7690965 | .2740772 | .9298486 | 0.768 | .489669 |
| **Manual therapy - Usual care** | .6130723 | .4016588 | .8871557 | .8387265 | -.2740834 | .9298459 | 0.768 | .4896684 |
| **NSAIDs - Usual care** | -.078838 | .3258861 | 1.179.435 | .5933446 | -1.258.273 | .6769487 | 0.063 | .2732733 |

* All the evidence about these contrasts comes from the trials which directly compare them.



Gianola S, *et al. Br J Sports Med* 2021; 56:41–50. doi: 10.1136/bjsports-2020-103596

## Table 2c. Nodesplit pain 12 months

| Side | Direct | | Indirect | | Difference | | | tau |
|---|---|---|---|---|---|---|---|---|
| | Coef. | Std. Err. | Coef. | Std. Err. | Coef. | Std. Err. | P>z | |
| **Inert treatment - Education*** | -.3029187 | .34666 | .3777316 | 158.3944 | -.6806503 | 158.3948 | 0.997 | .3164487 |
| **Cognitive CBT - Usual care*** | .0943039 | .2527336 | -1.379709 | 447.7409 | 1.474013 | 447.7409 | 0.997 | .316448 |
| **Education - Exercise*** | -.385339 | .3509876 | .3660218 | 174.4564 | -.7513608 | 174.4568 | 0.997 | .3164487 |
| **Exercise - Usual care*** | -9.18e-11 | .3653395 | .8080591 | 209.9836 | -.8080591 | 209.9839 | 0.997 | .3164485 |

* All the evidence about these contrasts comes from the trials which directly compare them.

# Table 3. Estimated Local Inconsistency for each pairwise comparison (side splitting) – disability outcome

## Table 3a. Nodesplit disability 1 week

| Side | Direct | | Indirect | | Difference | | | tau |
|---|---|---|---|---|---|---|---|---|
| | Coef. | Std. Err. | Coef. | Std. Err. | Coef. | Std. Err. | P>z | |
| **Inert Treatment-Acupuncture** | -0.3850695 | 0.3512901 | 0.318208 | 0.412454 | -0.7032775 | 0.541778 | 0.194 | 0.269133 |
| **Inert Treatment- Education** | -0.2712998 | 0.3261325 | -0.18365 | 0.424351 | -0.0876449 | 0.535197 | 0.87 | 0.293896 |
| **Inert Treatment-Heat wrap** | -0.5423379 | 0.2294745 | -0.17954 | 0.253958 | -0.3627932 | 0.342356 | 0.289 | 0.259164 |
| **Inert Treatment-Manual therapy** | -0.664142 | 0.2886231 | -0.59046 | 0.501075 | -0.0736865 | 0.581203 | 0.899 | 0.292533 |
| **Inert Treatment-Muscle relaxant** | . | . | . | . | . | . | . | . |
| **Inert Treatment-NSAIDs** | -0.387447 | 0.2022145 | -0.59797 | 0.251741 | 0.2105194 | 0.324018 | 0.516 | 0.293991 |
| **Inert Treatment-Paracetamol** | -0.0922448 | 0.2390906 | -0.67043 | 0.219723 | 0.5781899 | 0.324719 | 0.075 | 0.231374 |
| **Acupuncture- NSAIDs** | -0.731988 | 0.38266 | -0.02871 | 0.383529 | -0.7032779 | 0.541778 | 0.194 | 0.269133 |
| **Education- Exercise** | -0.2919225 | 0.4040913 | -0.93469 | 0.632299 | 0.6427636 | 0.750304 | 0.392 | 0.290215 |
| **Education- Heat wrap** | -0.4121889 | 0.3985883 | 0.083842 | 0.365582 | -0.4960307 | 0.540926 | 0.359 | 0.281415 |
| **Exercise-Heat wrap** | -0.1227089 | 0.3721725 | 1.177.067 | 0.505458 | -1.299.776 | 0.627943 | **0.038** | 0.241674 |
| **Exercise- Manual therapy** | 0.7716 | 0.4925257 | -0.52044 | 0.434413 | 1.292.041 | 0.656732 | **0.049** | 0.24743 |

| | | | | | | | | |
|---|---|---|---|---|---|---|---|---|
| **Heat wrap- NSAIDs** | -0.5127726 | 0.274752 | 0.1945 | 0.237414 | -0.7072724 | 0.36315 | 0.051 | 0.238334 |
| **Heat wrap- Paracetamol** | -0.4646165 | 0.2367674 | 0.3788 | 0.239479 | -0.8434166 | 0.336712 | **0.012** | 0.195007 |
| **Manual therapy- NSAIDs** | 0.7923256 | 0.328629 | -0.40012 | 0.328938 | 1.192.444 | 0.463877 | **0.01** | 0.226649 |
| **NSAIDs-Paracetamol** | -0.0008166 | 0.2354043 | 0.15986 | 0.348297 | -0.1606761 | 0.420353 | 0.702 | 0.293809 |

* All the evidence about these contrasts comes from the trials which directly compare them; inconsistency in bold constrast are >5% of the all comparisons

# Table 3b. Nodesplit disability 1 month

| Side | Direct | | Indirect | | Difference | | | tau |
|---|---|---|---|---|---|---|---|---|
| | Coef. | Std. Err. | Coef. | Std. Err. | Coef. | Std. Err. | P>z | |
| **Inert Treatment -Acupuncture** | -0.7093169 | 0.6236239 | 0.7481728 | 1.055.844 | -145.749 | 122.626 | 0.235 | 0.579317 |
| **Inert Treatment-Exercise** | 0.6744899 | 0.7305522 | 0.3343372 | 0.9563461 | 0.3401527 | 1.203.455 | 0.777 | 0.705391 |
| **Inert Treatment-Manual Therapy** | -0.819488 | 0.772666 | -0.4793281 | 0.92265 | -0.34016 | 1.203.452 | 0.777 | 0.705389 |
| **Inert Treatment- Paracetamol** | -0.0194038 | 0.5824383 | -1.476.859 | 1.079.109 | 1.457.455 | 1.226.259 | 0.235 | 0.579317 |
| **Acupuncture-NSAIDs** | -0.6397983 | 0.6390752 | 0.8176958 | 1.046.569 | -1.457.494 | 1.226.264 | 0.235 | 0.579317 |
| **Cognitive CBT-Usual care *** | 0.0188224 | 0.6228875 | -0.1682687 | 6.329.995 | 0.1870911 | 6.329.998 | 1.000 | 0.612493 |
| **Education-Exercise *** | -0.4262689 | 0.5999444 | -2.366.002 | 1.562.167 | 1.939.733 | 1.667.265 | 0.245 | 0.580495 |
| **Education-Manual therapy *** | -2.158.292 | 0.6063919 | -0.2185552 | 155.468 | -1.939.737 | 1.667.265 | 0.245 | 0.580495 |
| **Exercise- Manual therapy *** | -1.732.024 | 0.5978718 | -0.7621531 | 0.5809457 | -0.9698712 | 0.8336358 | 0.245 | 0.580497 |
| **Exercise- Usual care** | -1.82E-10 | 0.4822981 | -1.423.537 | 0.5431255 | 1.423.537 | 0.7263586 | 0.05 | 0.446406 |
| **Manual Therapy-Usual care** | 0.2390929 | 0.3731235 | 1.662.631 | 0.6231943 | -1.423.538 | 0.7263602 | 0.05 | 0.446407 |
| **NSAIDs- Paracetamol** | -0.127779 | 0.6059484 | 1.329.688 | 1.066.091 | -1.457.467 | 1.226.264 | 0.235 | 0.579317 |
| **NSAIDs- Steroids *** | -1.214.723 | 0.6700337 | 1.142.942 | 630.608 | -2.357.665 | 6.306.084 | 0.997 | 0.612493 |

* All the evidence about these contrasts comes from the trials which directly compare them.

# Table 3c. Nodesplit disability 12 months

| Side | Direct | | Indirect | | Difference | | | tau |
|---|---|---|---|---|---|---|---|---|
| | Coef. | Std. Err. | Coef. | Std. Err. | Coef. | Std. Err. | P>z | |
| **Inert treatment-Education*** | -0.162517 | 0.323069 | 0.382189 | 141.004 | -0.54471 | 1.410.044 | 0.997 | 0.290697 |
| **Cognitive CBT-Exercise** | 0.088617 | 0.446814 | 0.174454 | 0.492926 | -0.08584 | 0.6648704 | 0.897 | 0.369949 |
| **Cognitive CBT-Usual care*** | 0.3264051 | 0.226606 | -0.35701 | 1.060.696 | 0.683413 | 1.086.459 | 0.529 | 0.336763 |
| **Education-Exercise*** | -0.436679 | 0.328125 | 0.151605 | 1.535.627 | -0.58828 | 153.563 | 0.997 | 0.290697 |
| **Exercise-Usual care *** | 0.2022777 | 0.296387 | -0.12221 | 0.932483 | 0.32449 | 0.9785033 | 0.74 | 0.354265 |

* All the evidence about these contrasts comes from the trials which directly compare them.

# Table 4. Strategy to explore global inconsistency – disability 1 week

| | Study removed | Chi square | Prob > chi2 | Resolving inconsistency |
|---|---|---|---|---|
| **All studies** | | chi2 (8) = 28.66 | Prob > chi2 = 0.0004* | |
| **STRATEGY 1: nodesplitting** | | | | |
| **All studies without inconsistent constast (Exercise-Heat wrap)** | Mayer 2005 | chi2 (6) = 21.33 | Prob > chi2 = 0.0016* | Not resolved |
| **All studies without inconsistent constast (Exercise- Manual therapy)** | Shrenk 2003 | chi2 (7) = 22.93 | Prob > chi2 = 0.0018* | Not resolved |
| **All studies without inconsistent constast (Heat wrap-Paracetamol)** | Nadler 2002 | chi2 (6) = 14.38 | Prob > chi2 = 0.0257* | Not resolved |
| **All studies without inconsistent constast (Manual therapy-NSAIDs)** | von Heymann 2013 | chi2 (6) = 19.47 | Prob > chi2 = 0.0034* | Not resolved |
| **All studies without the four previous inconsistent constasts** | All studies above | chi2 (2) = 6.03 | Prob > chi2 = 0.0491* | Not resolved |
| **STRATEGY 2: inspection of covariates** | | | | |
| **Metaregression** | The effects of the investigated co-variates were not statistically significant. See Table 6a | | | Not resolved |
| **STRATEGY 3: inspection of subgroups** | | | | |
| **Subgroup analysis (splitting pharmacological from non-pharmacological intervention)** | Dreiser 2003; Miki 2018; Nadler 2002; Ralph 2008; Serfer 2009; Shin 2013; von Heymann 2013 (arm NSAIDs); Williams 2014 | chi2 (2) = 3.19 | Prob > chi2 = 0.2030 | Resolved |
| **Subgroup analysis (splitting non-pharmacological from pharmacological intervention)** | Hasegawa 2014; Mayer 2005; Nadler 2002 (arm heat wrap); Nadler 2003a; Nadler 2003b; Schenk 2003; Shin 2013; Takamoto 2015; Traeger 2019; von Heymann 2013 (arm manual therapy) | chi2 (1) = 2.14 | Prob > chi2 = 0.1432 | Resolved |

* Global consistency is tested here using the 'design-by-interaction' test that infers consistency across an entire treatment network, using a chi square test. A p value <0.05 is taken to infer evidence of global inconsistency in the network. [24 25]

# Table 5. Strategy to explore global inconsistency – disability 1 month

| | Study removed | Chi square | Prob > chi2 | Resolving inconsistency |
|---|---|---|---|---|
| **All studies** | | chi2 (3) =11.20 | Prob > chi2 = 0.0107* | See network meta forest |
| **STRATEGY 1: nodesplitting** | | | | |
| **All studies without inconsistent constast** | No contrast statistically significant | | | Not resolved |
| **STRATEGY 2: inspection of covariates** | | | | |
| **Metaregression** | The effects of the investigated co-variates were not statistically significant. See Table 6b | | | Not resolved |
| **STRATEGY 3: inspection of subgroups** | | | | |
| **Subgroup analysis (splitting pharmacological from non-pharmacological intervention)** | Miki 2008, Sea-Jung 2016; Shin 2013, Williams 2014 | chi2 (2) = 7.15 | Prob > chi2 = 0.0280* | Not resolved; See network meta forest |
| **Subgroup analysis (splitting non-pharmacological from pharmacological intervention)** | Cherkin 1998, Hasegawa 2014, Jellema 2005, Malmivaara 1995, Schneider 2015, Seferlis 1998, Shin 2013, Takamoto 2015 | chi2 (1) = 19.69 | Prob > chi2 = 0.0000* | Not resolved; See network meta forest |

* Global consistency is tested here using the 'design-by-interaction' test that infers consistency across an entire treatment network, using a chi square test. A p value <0.05 is taken to infer evidence of global inconsistency in the network. [24 25]

# Table 6a. Metaregression disability 1 week

| Variable | Coeff. | St. error | P>[t] | Tau2 | 95% CI | |
|---|---|---|---|---|---|---|
| **Age** | 0.003 | 0.008 | 0.699 | 0.067 | -0.014 | 0.021 |
| **Gender** | 0.005 | 0.007 | 0.477 | 0.067 | -0.010 | 0.021 |
| **Patients with subacute/acute pain** | -0.022 | 0.077 | 0.782 | 0.067 | -0.181 | 0.138 |
| **Baseline value of pain** | -0.008 | 0.007 | 0.244 | 0.098 | -0.023 | 0.006 |
| **Presence of leg pain or sciatica** | -0.039 | 0.143 | 0.783 | 0.069 | -0.337 | 0.257 |
| **Risk of bias** | 0.124 | 0.104 | 0.246 | 0.067 | -0.092 | 0.342 |

# Table 6b. Metaregression disability 1 month

| Variable | Coeff. | St. error | P>[t] | Tau2 | 95% CI | |
|---|---|---|---|---|---|---|
| **Age** | 0.014 | 0.034 | 0.677 | 0.664 | -0.059 | 0.088 |
| **Gender** | -0.043 | 0.022 | 0.071 | 0.504 | -0.090 | 0.004 |
| **Patients with subacute/acute pain** | -0.257 | 0.213 | 0.252 | 0.591 | -0.721 | 0.207 |
| **Baseline value of pain** | -0.017 | 0.026 | 0.533 | 0.651 | -0.073 | 0.039 |
| **Presence of leg pain or sciatica** | -0.113 | 0.235 | 0.638 | 0.660 | -0.624 | 0.398 |
| **Risk of bias** | 0.008 | 0.259 | 0.976 | 0.674 | -0.571 | 0.555 |

# Figure 1. Bubble plot disability 1 week

Age

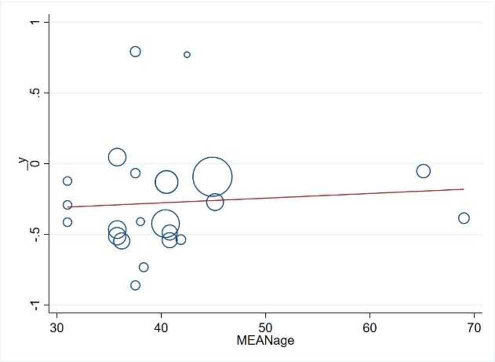


Gender

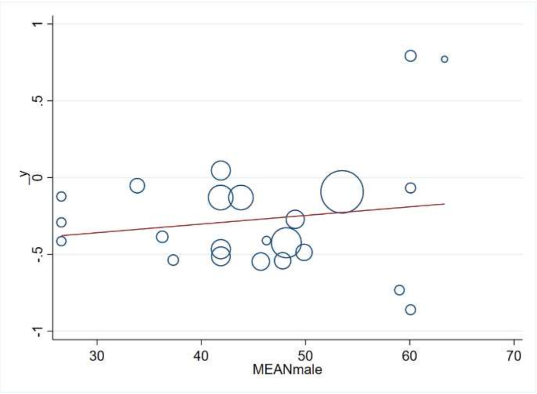


Patients with subacute/acute pain

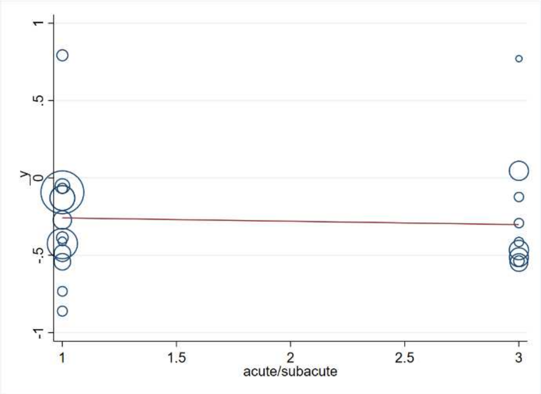


Baseline value of pain

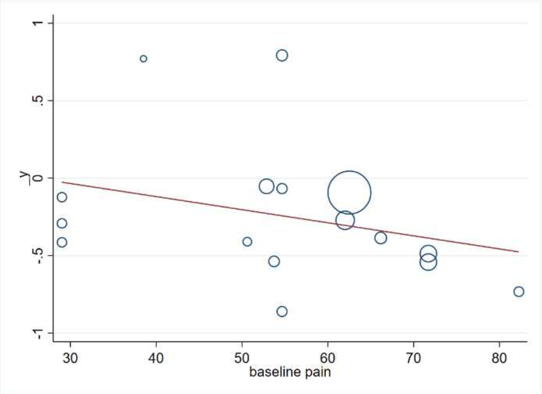


Presence of leg pain or sciatica

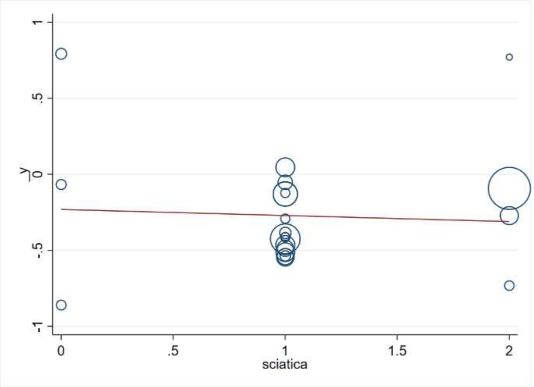


Risk of bias

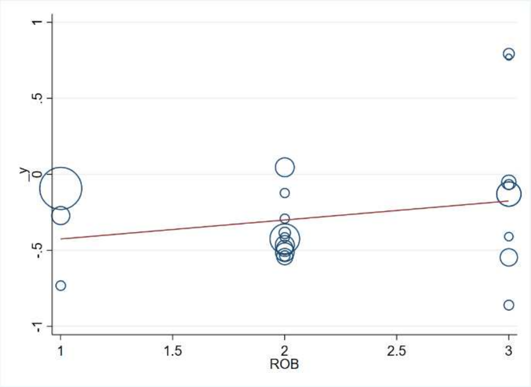

# Figure 2. Bubble plot disability 1 month

Age

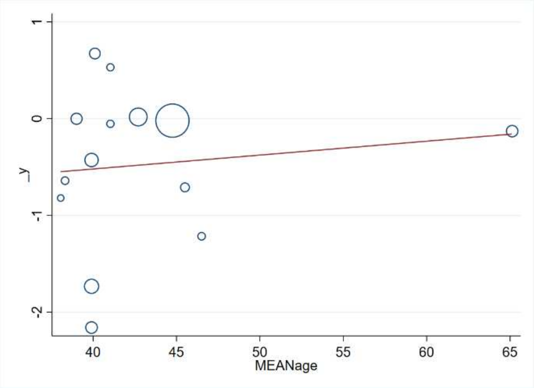


Gender

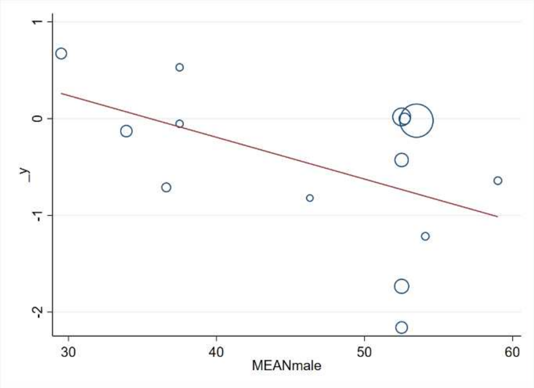


Patients with subacute/acute pain

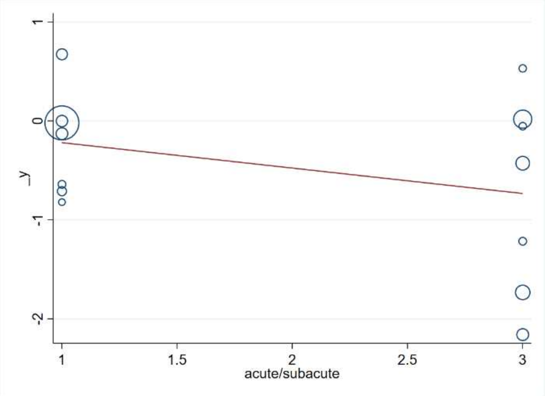


Baseline value of pain

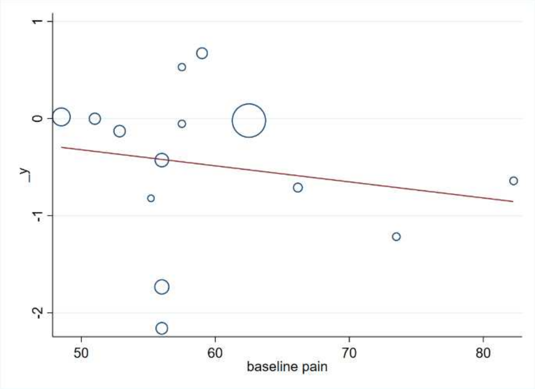


Presence of leg pain or sciatica

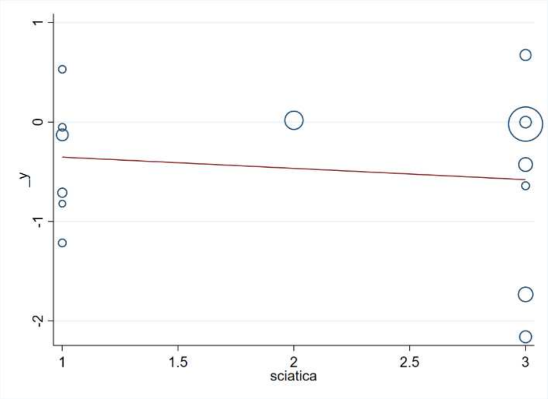


Risk of bias

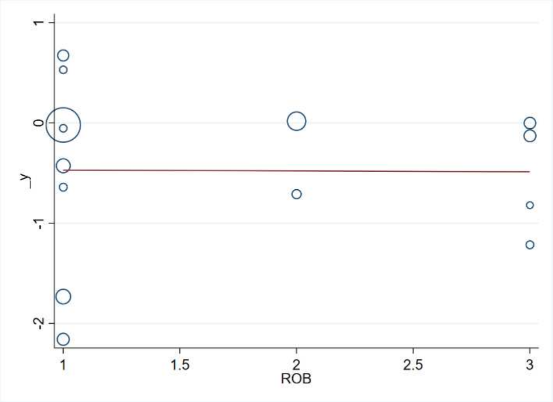

## Supplementary K. Subgroup analysis results

### 1. Subgroup meta-analysis (pharmacological and non-pharmacological)

### Disability 1 week – non pharmacological treatments

### Figure 1a. Network plot of non-pharmacological treaments

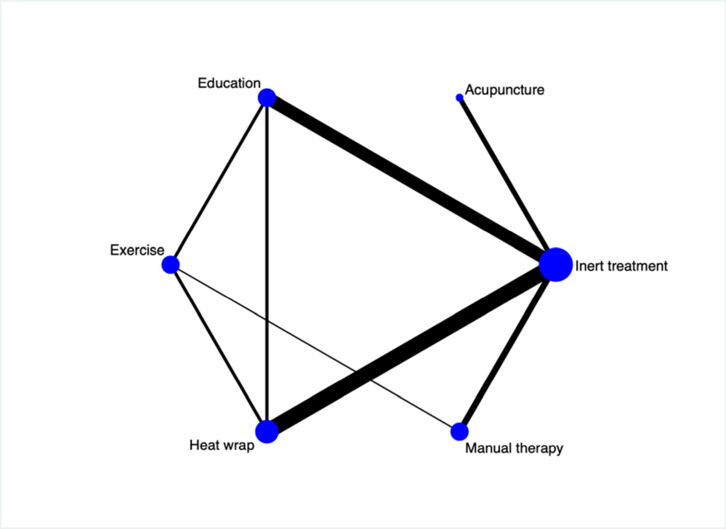


Testing for inconsistency: chi2(2) = 3.19; Prob > chi2 = 0.2030

### Figure 2a. Network forest of non-pharmacological treaments

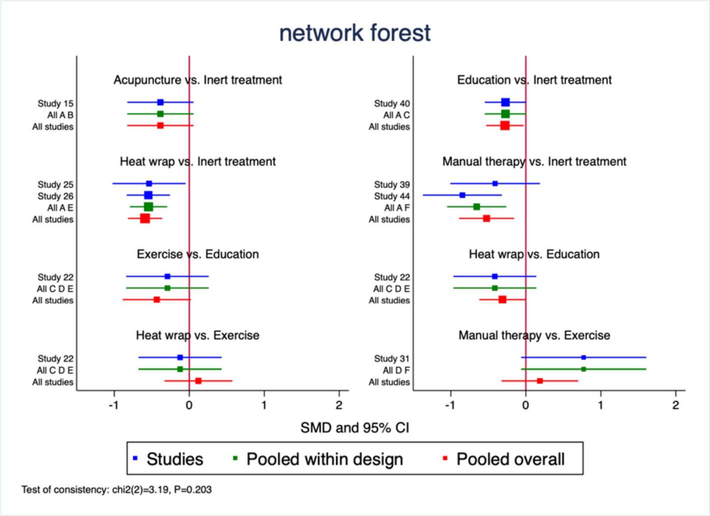

# Table 1a. Netleague of non-pharmacological treaments

| | | | | | |
|---|---|---|---|---|---|
| Inert treatment | -0.39 (-0.83,0.06) | -0.28 (-0.53,-0.03) | -0.71 (-1.16,-0.26) | -0.59 (-0.82,-0.36) | -0.52 (-0.89,-0.16) |
| 0.39 (-0.06,0.83) | Acupuncture | 0.11 (-0.40,0.61) | -0.33 (-0.96,0.30) | -0.20 (-0.70,0.29) | -0.14 (-0.71,0.44) |
| 0.28 (0.03,0.53) | -0.11 (-0.61,0.40) | Education | -0.43 (-0.89,0.02) | -0.31 (-0.62,-0.00) | -0.25 (-0.68,0.19) |
| 0.71 (0.26,1.16) | 0.33 (-0.30,0.96) | 0.43 (-0.02,0.89) | Exercise | 0.12 (-0.33,0.57) | 0.19 (-0.32,0.70) |
| 0.59 (0.36,0.82) | 0.20 (-0.29,0.70) | 0.31 (0.00,0.62) | -0.12 (-0.57,0.33) | Heatwrap | 0.07 (-0.36,0.49) |
| 0.52 (0.16,0.89) | 0.14 (-0.44,0.71) | 0.25 (-0.19,0.68) | -0.19 (-0.70,0.32) | -0.07 (-0.49,0.36) | Manual therapy |

# Table 2a. SUCRA of non-pharmacological treaments

| Treatment | SUCRA | PrBest | MeanRank |
|---|---|---|---|
| **Manual therapy** | 80,3 | 43,6 | 2 |
| **Exercise** | 69,4 | 35,4 | 2,5 |
| **Heatwrap** | 67,9 | 12,6 | 2,6 |
| **Acupuncture** | 48,4 | 8,4 | 3,6 |
| **Education** | 31,2 | 0 | 4,4 |
| **Inert treatment** | 2,9 | 0 | 5,9 |

# Disability 1 week – pharmacological treatments

## Figure 1b. Network plot of pharmacological treaments

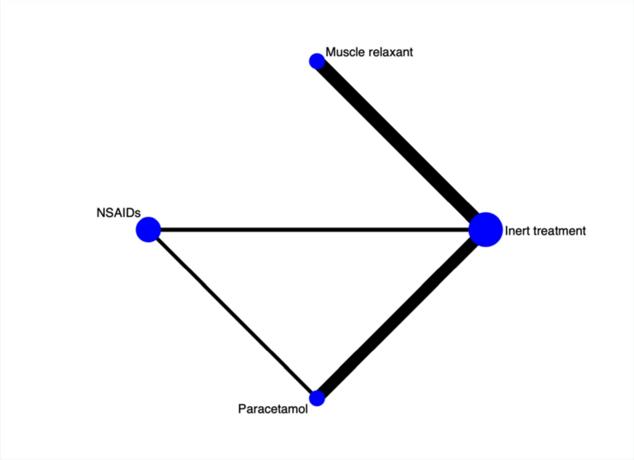


Testing for inconsistency: chi2(1) = 2.14; Prob > chi2 = 0.1432

## Figure 2b. Network forest of pharmacological treaments

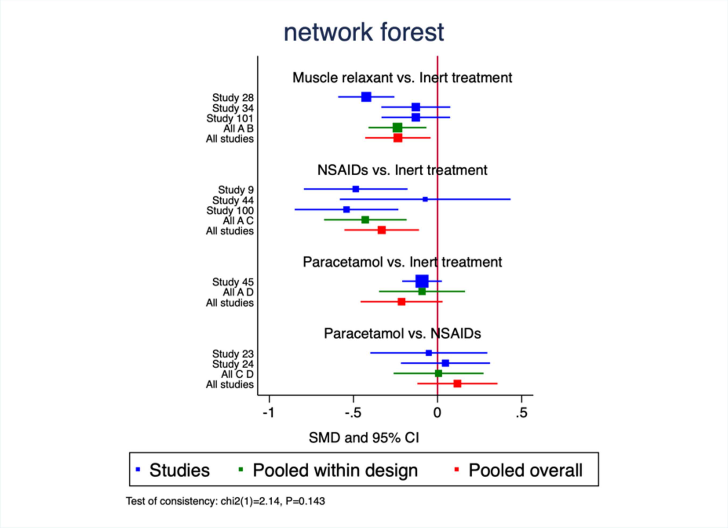

# Table 1b. Netleague of pharmacological treaments

| | | | |
|---|---|---|---|
| Inert treatment | -0.24 (-0.43,-0.04) | -0.33 (-0.55,-0.11) | -0.21 (-0.46,0.03) |
| 0.24 (0.04,0.43) | Muscle relaxant | -0.10 (-0.39,0.20) | 0.02 (-0.29,0.34) |
| 0.33 (0.11,0.55) | 0.10 (-0.20,0.39) | NSAIDs | 0.12 (-0.12,0.36) |
| 0.21 (-0.03,0.46) | -0.02 (-0.34,0.29) | -0.12 (-0.36,0.12) | Paracetamol |

# Table 2b. SUCRA of pharmacological treaments

| Treatment | SUCRA | PrBest | MeanRank |
|---|---|---|---|
| **NSAIDs** | 94,6 | 86 | 1,2 |
| **Muscle relaxant** | 64,1 | 11 | 2,1 |
| **Paracetamol** | 33,3 | 3 | 3 |
| **Inert treatment** | 7,9 | 0 | 3,8 |

# Disability 1 month – non pharmacological treatments

## Figure 3a. Network plot of non-pharmacological treaments

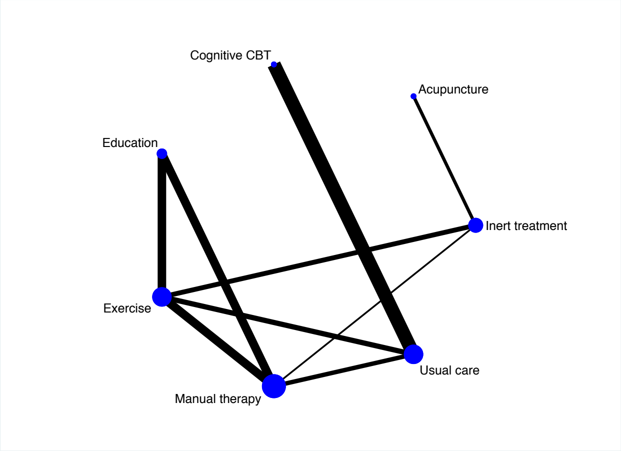


Since we found sources of inconsistency (Prob > chi2 =0.0280) in non-pharmacological network, we presented only pairwise meta-analyses and NMA

## Figure 4a. Network forest of non-pharmacological treaments

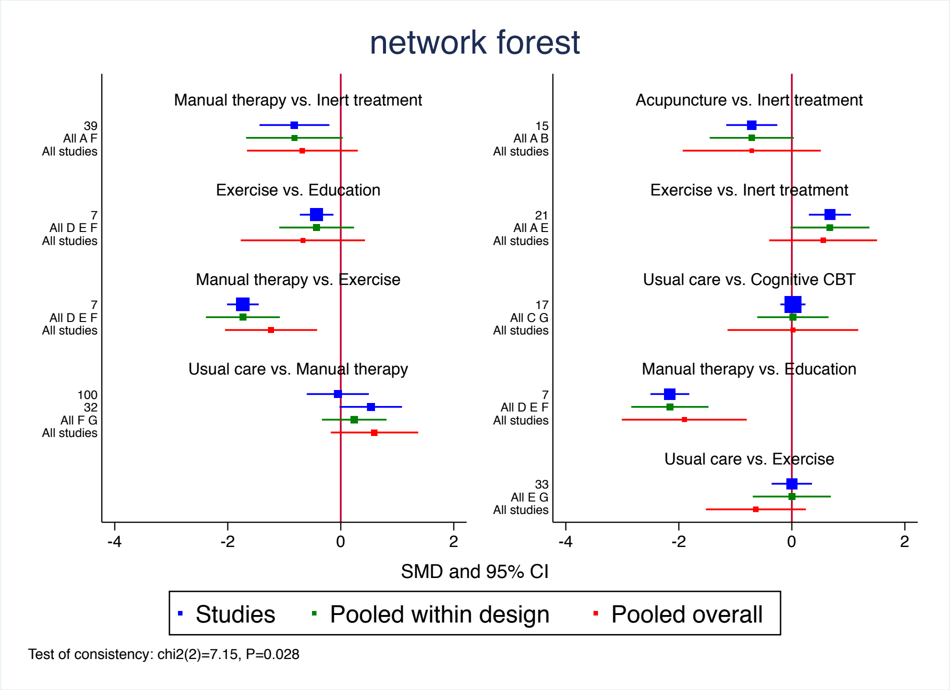

| Comparison | ES | [95% Conf. | Interval] | z | p value | I2 | Tau-squared |
|---|---|---|---|---|---|---|---|
| *Usual care-Manual Therapy* | | | | | | | |
| *2 studies* | -0.052 | -0.601 | 0.497 | | | | |
| | 0.531 | -0.022 | 1.085 | | | | |
| *overall* | 0.239 | -0.333 | 0.81 | z= 0.82 | p = 0.413 | 53.5% | 0.0910 |
| *Acupuncture-Inert treatment* | | | | | | | |
| *1 study* | -0.709 | -1.162 | -0.257 | z= 3.07 | p = 0.002 | | |
| *Usual care-Cognitive CBT* | | | | | | | |
| *1 study* | 0.019 | -0.203 | 0.241 | z= 0.17 | p = 0.868 | | |
| *Exercise-Inert treatment* | | | | | | | |
| *1 study* | 0.674 | 0.302 | 1.047 | z= 3.55 | p = 0.000 | | |
| *Usual care-Exercise* | | | | | | | |
| *1 study* | 0 | -0.358 | 0.358 | z= 3.55 | p = 0.000 | | |
| *Manual Therapy-Inert treatment* | | | | | | | |
| *1 study* | -0.819 | -1.438 | -0.201 | z= 2.60 | p = 0.009 | | |
| *Exeercise-Education* | | | | | | | |
| *1 study* | -0.426 | -0.723 | -0.129 | z= 2.81 | p = 0.005 | | |
| *Manual Therapy - Education* | | | | | | | |
| *1 study* | -2.158 | -2.502 | -1.815 | z= 12.31 | p = 0.000 | | |
| *Manual Therapy-Exercise* | | | | | | | |
| *1 study* | -1.732 | -2.012 | -1.452 | z= 12.10 | p = 0.000 | | |



Gianola S, *et al. Br J Sports Med* 2021; 56:41–50. doi: 10.1136/bjsports-2020-103596

# Disability 1 month – pharmacological treatments

## Figure 3b. Network plot of pharmacological treaments

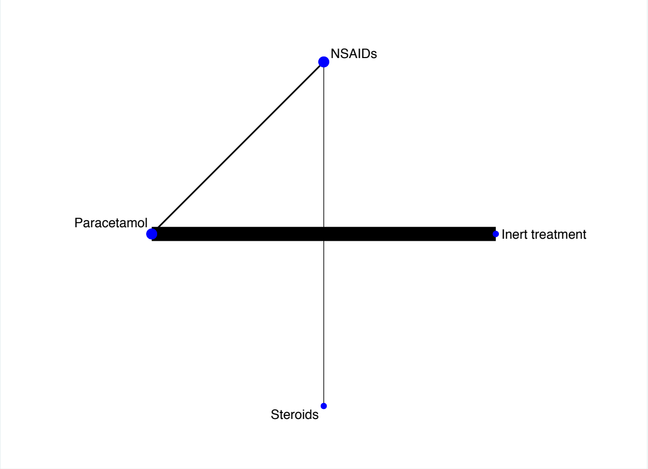


Since we found sources of inconsistency (Prob > chi2 = 0.000) in non-pharmacological network, we presented only pairwise meta-analyses and NMA

## Figure 4b. Network forest of pharmacological treaments

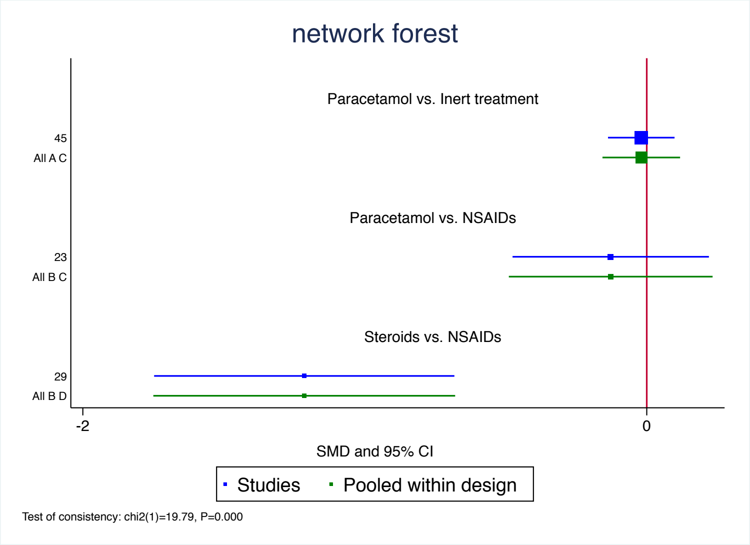


| *Comparisons* | *ES* | *[95% Conf.* | *Interval]* | *z* | *p-value* |
|---|---|---|---|---|---|
| *Paracetamol-NSAIDs* | | | | | |
| *1 study* | -0.128 | -0.476 | 0.22 | z= 0.72 | p = 0.472 |
| *Steroids-NSAIDs* | | | | | |
| *1 study* | -1.215 | -1.747 | -0.682 | z= 4.47 | p = 0.000 |
| *Paracetamol-Inert treatment* | | | | | |
| *1 study* | -0.019 | -0.137 | 0.099 | z= 0.32 | p = 0.747 |

## Supplementary L. Network meta-analysis results- Interval plot

Figure 1. Interval Plot -Network Meta-Analyses – Pain outcome

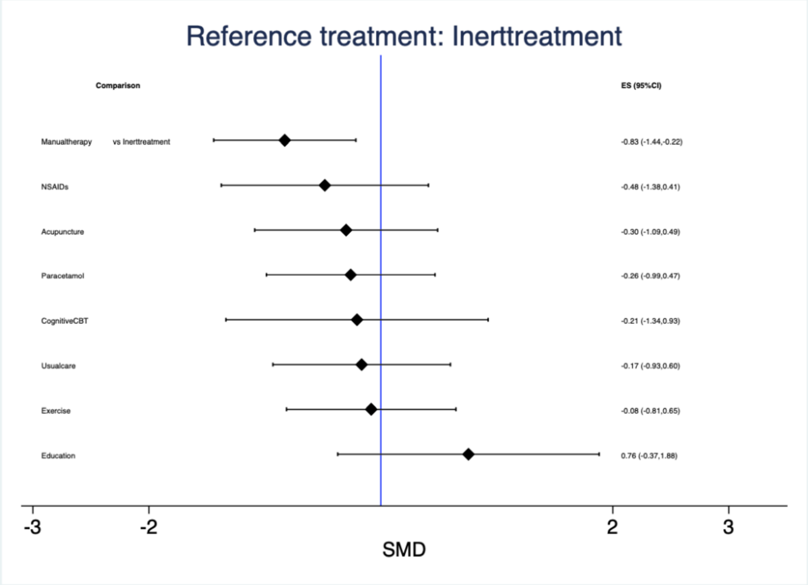


Figure 1a. Interval plot all treatments against inert treatment for pain outcome at 1 month of FU

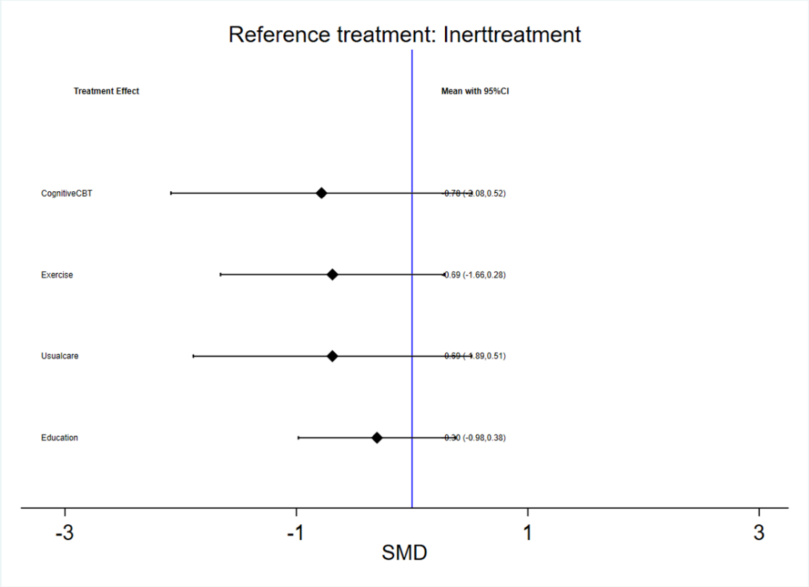


Figure 1b. Interval plot all treatments against inert treatment for pain outcome at 12 months of FU

Figure 2. Interval Plot -Network Meta-Analyses – Disability Outcome

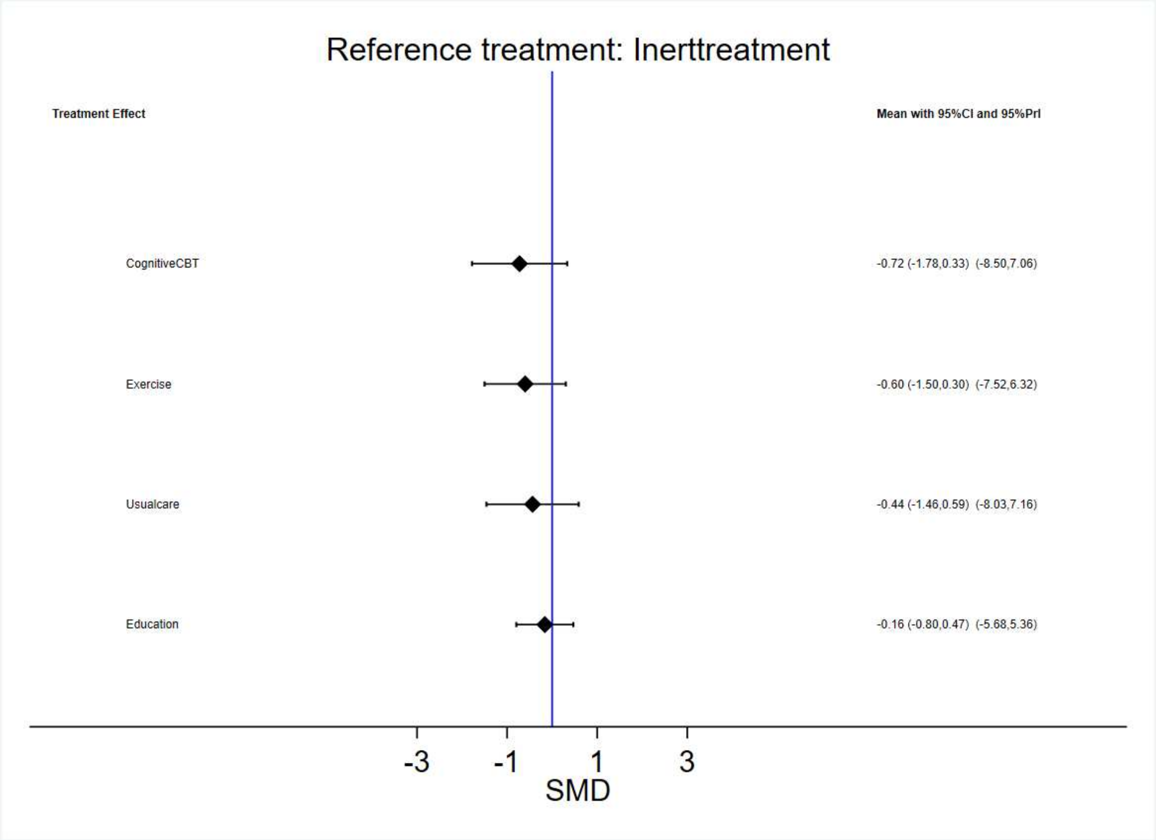


Figure 2a. Interval plot all treatments against inert treatment for disability outcome at 12 months of FU

## Supplement M. All treatments against all treatments

# Table 1. League table - pain

# Table 1a. League table pain 1 month

| | | | | | | | | |
|---|---|---|---|---|---|---|---|---|
| Inert treatment | -0.30 (-1.09,0.49) | -0.21 (-1.34,0.93) | 0.76 (-0.37,1.88) | -0.08 (-0.81,0.65) | -0.83 (-1.44,-0.22) | -0.48 (-1.38,0.41) | -0.26 (-0.99,0.47) | -0.17 (-0.93,0.60) |
| 0.30 (-0.49,1.09) | Acupuncture | 0.09 (-1.28,1.47) | 1.05 (-0.32,2.43) | 0.22 (-0.86,1.29) | -0.53 (-1.53,0.47) | -0.18 (-1.00,0.63) | 0.04 (-0.85,0.93) | 0.13 (-0.96,1.23) |
| 0.21 (-0.93,1.34) | -0.09 (-1.47,1.28) | Cognitive CBT | 0.96 (-0.44,2.36) | 0.12 (-0.98,1.23) | -0.62 (-1.66,0.42) | -0.28 (-1.71,1.16) | -0.05 (-1.40,1.29) | 0.04 (-0.79,0.87) |
| -0.76 (-1.88,0.37) | -1.05 (-2.43,0.32) | -0.96 (-2.36,0.44) | Education | -0.84 (-1.70,0.02) | -1.58 (-2.75,-0.42) | -1.24 (-2.67,0.20) | -1.02 (-2.35,0.32) | -0.92 (-2.05,0.21) |
| 0.08 (-0.65,0.81) | -0.22 (-1.29,0.86) | -0.12 (-1.23,0.98) | 0.84 (-0.02,1.70) | Exercise | -0.75 (-1.53,0.04) | -0.40 (-1.55,0.75) | -0.18 (-1.21,0.85) | -0.08 (-0.81,0.65) |
| 0.83 (0.22,1.44) | 0.53 (-0.47,1.53) | 0.62 (-0.42,1.66) | 1.58 (0.42,2.75) | 0.75 (-0.04,1.53) | Manual therapy | 0.35 (-0.73,1.42) | 0.57 (-0.38,1.51) | 0.66 (0.04,1.29) |
| 0.48 (-0.41,1.38) | 0.18 (-0.63,1.00) | 0.28 (-1.16,1.71) | 1.24 (-0.20,2.67) | 0.40 (-0.75,1.55) | -0.35 (-1.42,0.73) | NSAIDs | 0.22 (-0.54,0.99) | 0.32 (-0.85,1.49) |
| 0.26 (-0.47,0.99) | -0.04 (-0.93,0.85) | 0.05 (-1.29,1.40) | 1.02 (-0.32,2.35) | 0.18 (-0.85,1.21) | -0.57 (-1.51,0.38) | -0.22 (-0.99,0.54) | Paracetamol | 0.09 (-0.96,1.15) |
| 0.17 (-0.60,0.93) | -0.13 (-1.23,0.96) | -0.04 (-0.87,0.79) | 0.92 (-0.21,2.05) | 0.08 (-0.65,0.81) | -0.66 (-1.29,-0.04) | -0.32 (-1.49,0.85) | -0.09 (-1.15,0.96) | Usual care |

# Table 1b. League table pain 12 months

| | | | | |
|---|---|---|---|---|
| Inert treatment | -0.69 (-1.89,0.51) | -0.69 (-1.66,0.28) | -0.30 (-0.98,0.38) | -0.78 (-2.08,0.52) |
| 0.69 (-0.51,1.89) | Usual care | -0.00 (-0.72,0.72) | 0.39 (-0.61,1.38) | -0.09 (-0.59,0.40) |
| 0.69 (-0.28,1.66) | 0.00 (-0.72,0.72) | Exercise | 0.39 (-0.30,1.07) | -0.09 (-0.96,0.78) |
| 0.30 (-0.38,0.98) | -0.39 (-1.38,0.61) | -0.39 (-1.07,0.30) | Education | -0.48 (-1.59,0.63) |
| 0.78 (-0.52,2.08) | 0.09 (-0.40,0.59) | 0.09 (-0.78,0.96) | 0.48 (-0.63,1.59) | Cognitive CBT |

# Table 2. Pain SUCRA

| 1 week of FU (immediate-term) | | | |
|---|---|---|---|
| **Treatment** | **SUCRA** | **PrBest** | **MeanRank** |
| Exercise | 89,2 | 40,8 | 2 |
| Heat wrap | 85,8 | 45,2 | 2,3 |
| Opioid | 68,6 | 9,6 | 3,8 |
| Manual therapy | 60 | 1,4 | 4,6 |
| Muscle relaxant | 50,2 | 2 | 5,5 |
| NSAIDs | 47,9 | 0,2 | 5,7 |
| Paracetamol | 40,7 | 0,6 | 6,3 |
| Education | 25,1 | 0 | 7,7 |
| Acupuncture | 21,8 | 0,2 | 8 |
| Inert treatment | 10,7 | 0 | 9 |
| **1 month of FU (short-term)** | | | |
| **Treatment** | **SUCRA** | **PrBest** | **MeanRank** |
| Manual therapy | 91,1 | 57,2 | 1,7 |
| NSAIDs | 71,4 | 20,8 | 3,3 |
| Acupuncture | 55,7 | 7,4 | 4,5 |
| Paracetamol | 55,3 | 5 | 4,6 |
| Cognitive CBT | 50,8 | 8,6 | 4,9 |
| Usual care | 46,3 | 0,2 | 5,3 |
| Exercise | 40,3 | 0,6 | 5,8 |
| Inert treatment | 34,2 | 0 | 6,3 |
| Education | 4,9 | 0,2 | 8,6 |
| **12 months (long term)** | | | |
| **Treatment** | **SUCRA** | **PrBest** | **MeanRank** |
| Cognitive CBT | 73.7 | 45.0 | 2.1 |
| Exercise | 66.0 | 26.0 | 2.4 |
| Usual care | 61.4 | 16.8 | 2.5 |
| Education | 33.6 | 8.4 | 3.7 |
| Inert treatment | 15.3 | 3.8 | 4.4 |

Figure 1. Cumulative ranking curve of pain 1 week

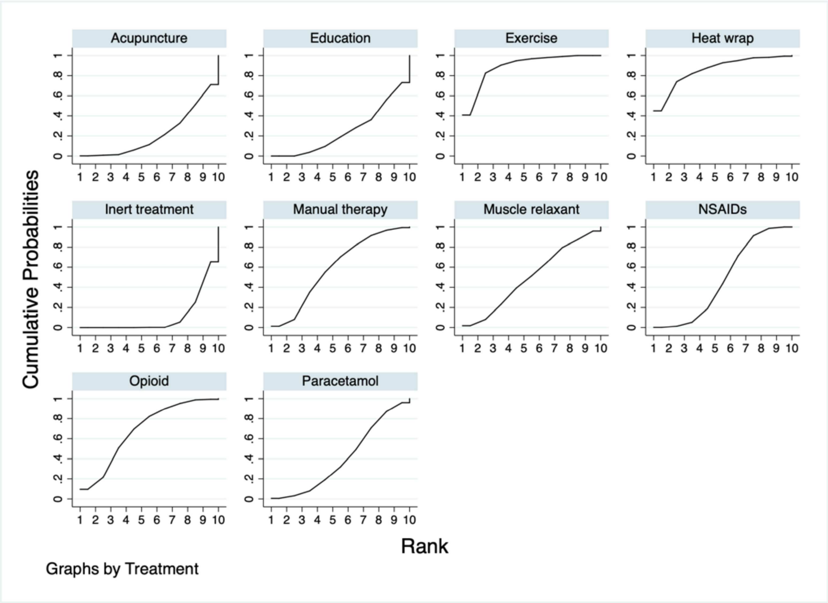


Figure 2. Cumulative ranking curve of pain 1 month

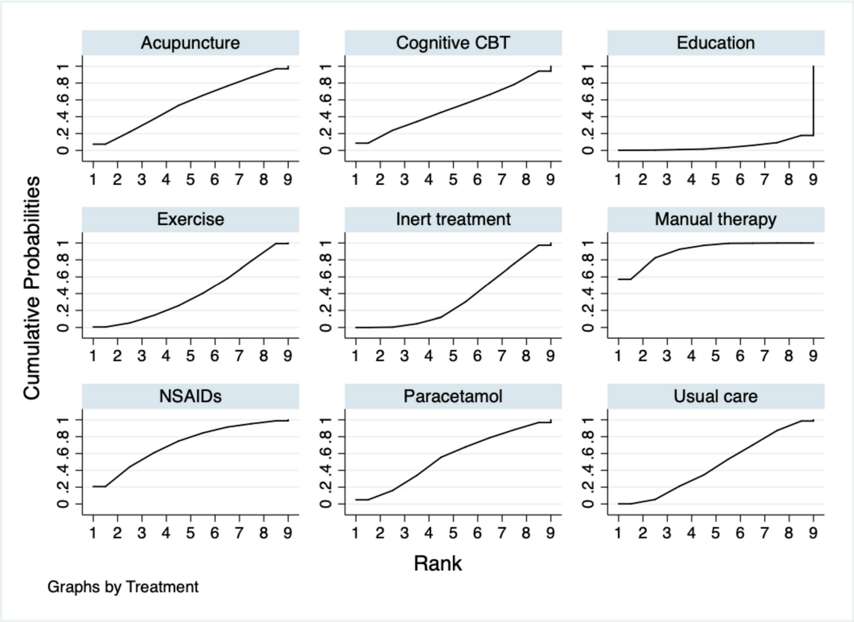

# Figure 3. Cumulative ranking curve of pain 12 months

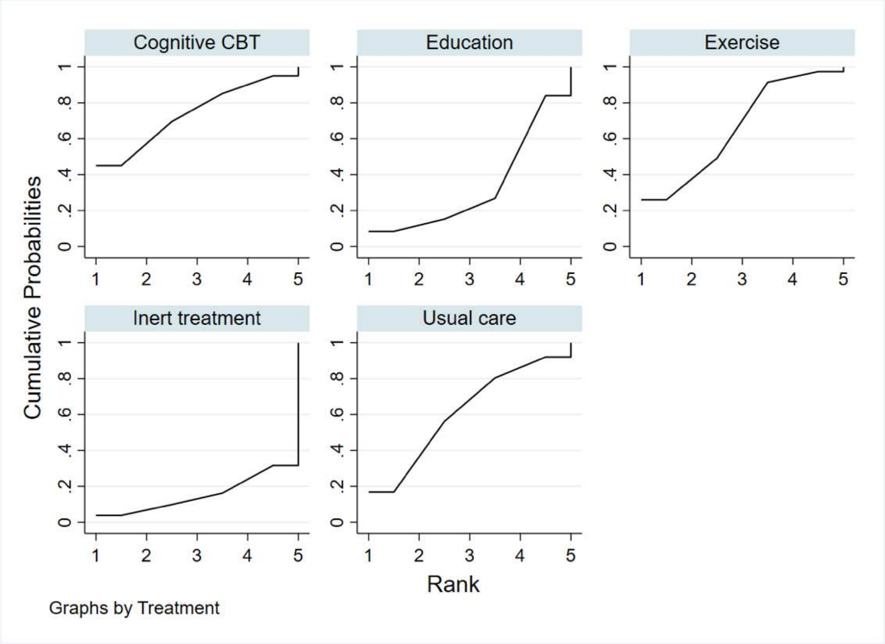




Gianola S, *et al. Br J Sports Med* 2021; 56:41–50. doi: 10.1136/bjsports-2020-103596

# Table 3. League table - disability

## Table 3a. League table disability 12 months

| | | | | |
|---|---|---|---|---|
| Inert treatment | -0.44 (-1.46,0.59) | -0.60 (-1.50,0.30) | -0.16 (-0.80,0.47) | -0.72 (-1.78,0.33) |
| 0.44 (-0.59,1.46) | Usual care | -0.16 (-0.65,0.32) | 0.27 (-0.53,1.08) | -0.29 (-0.68,0.10) |
| 0.60 (-0.30,1.50) | 0.16 (-0.32,0.65) | Exercise | 0.44 (-0.21,1.08) | -0.12 (-0.67,0.42) |
| 0.16 (-0.47,0.80) | -0.27 (-1.08,0.53) | -0.44 (-1.08,0.21) | Education | -0.56 (-1.41,0.28) |
| 0.72 (-0.33,1.78) | 0.29 (-0.10,0.68) | 0.12 (-0.42,0.67) | 0.56 (-0.28,1.41) | Cognitive CBT |

# Table 4. Disability SUCRA

| **12 month of FU (long term)** | | | |
|---|---|---|---|
| **Treatments** | **SUCRA** | **PrBest** | **MeanRank** |
| Cognitive CBT | 68.5 | 41 | 2.3 |
| Exercise | 66.5 | 20.2 | 2.3 |
| Usual care | 61.5 | 28.2 | 2.5 |
| Education | 30.9 | 3.8 | 3.8 |
| Inert treatment | 22.7 | 6.8 | 4.1 |

Figure 4. Cumulative ranking curve of disability 12 months

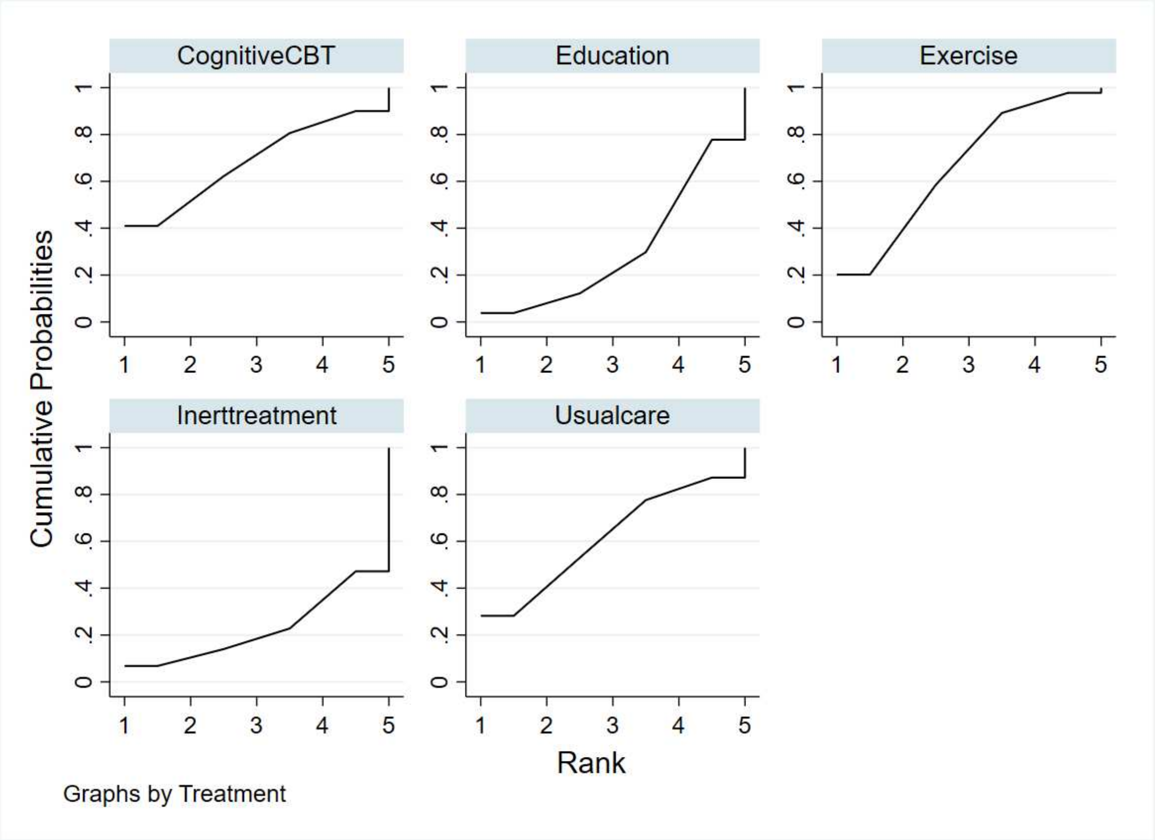

# Supplement N. Funnel Plot

Funnel plot asymmetry was used to assess publication bais containing 10 or more trials reporting the outcome of interest. Thus, this was possibile only for pain and disability outcomes at 1 week and 1 month of follow-up.

## Figure 1. Funnel plot-pain

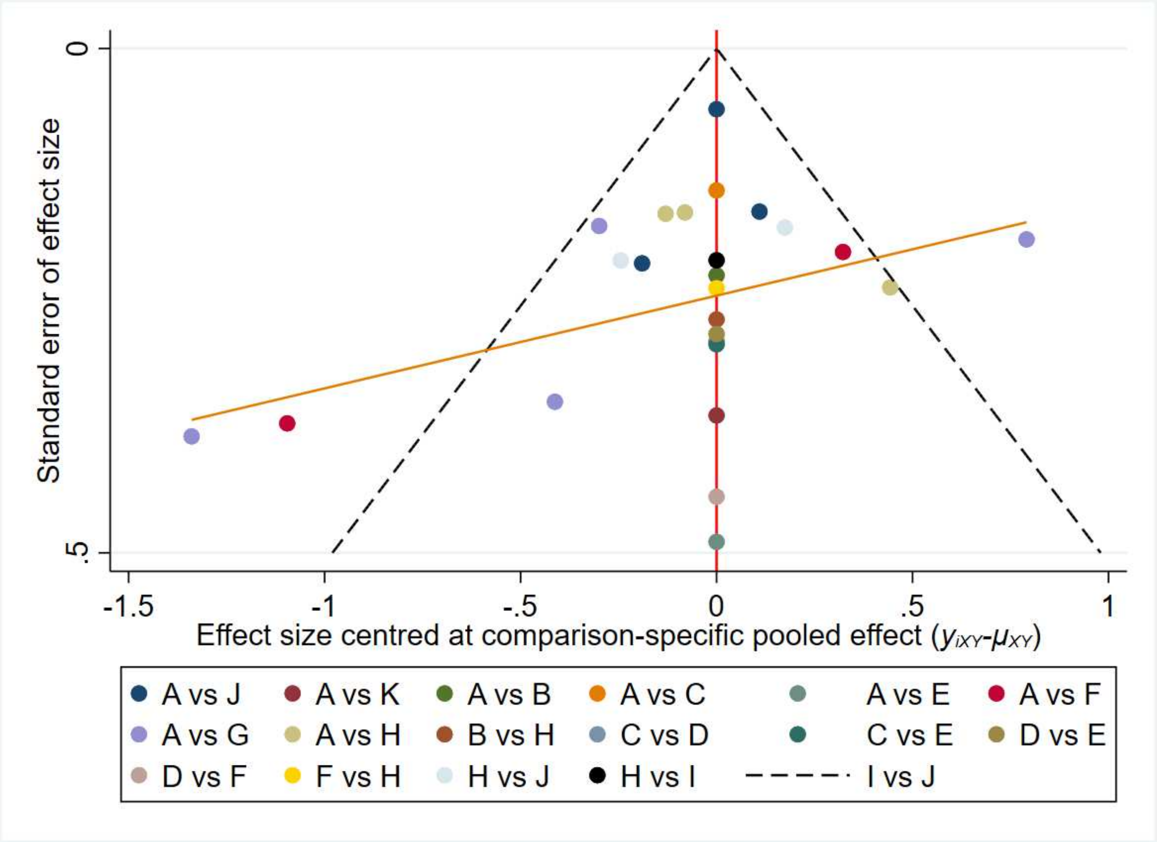


The red line represents the null hypothesis that the study-specific effect sizes do not differ from the respective comparison-specific pooled effect estimates. The orange line is the regression line.

## Figure 1a. Pain Outcome 1 week

legend: Treatments used

| | |
|---|---|
| A (reference): | Inert treatment |
| B: | Acupuncture |
| C: | Education |
| D: | Exercise |
| E: | Heat wrap |
| F: | Manual therapy |
| G: | Muscle relaxant |

H: NSAIDs
I: Opioid
J: Paracetamol
K: Physical therapy

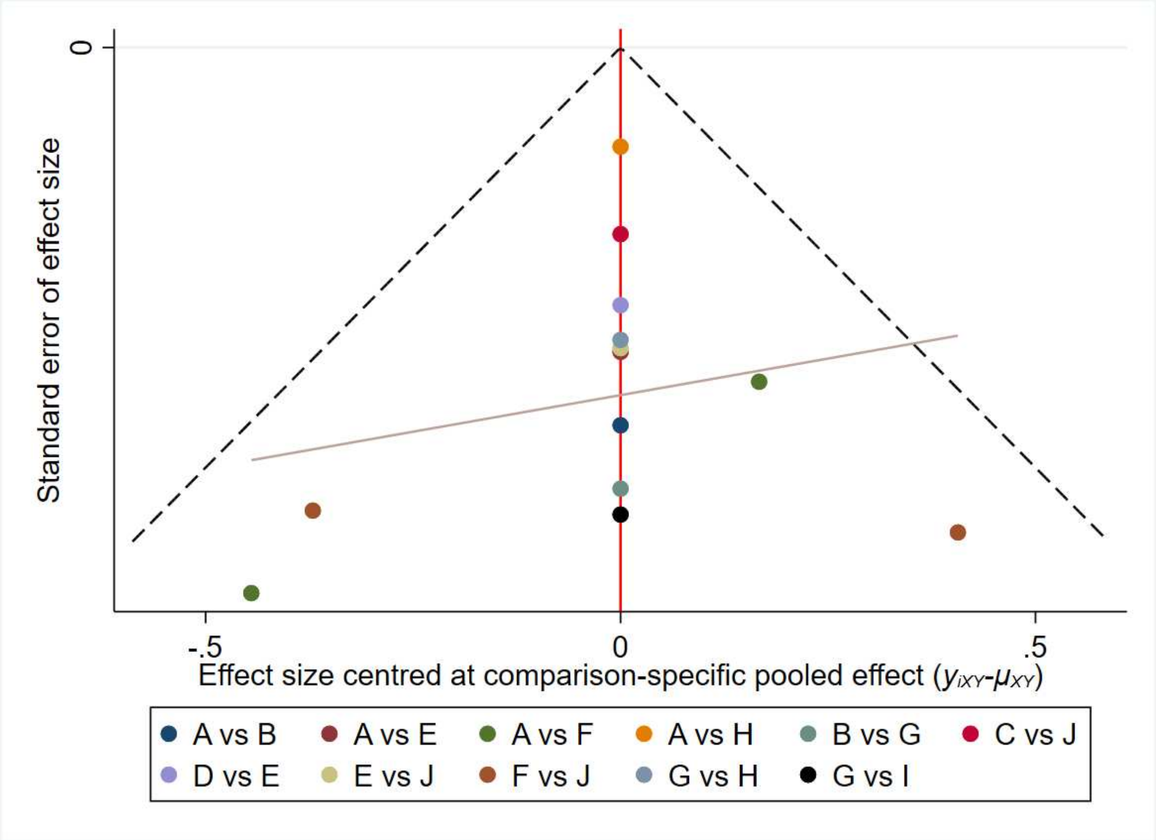


The red line represents the null hypothesis that the study-specific effect sizes do not differ from the respective comparison-specific pooled effect estimates. The gray line is the regression line.

## Figure 1b. Pain Outcome 1 month

Legend: Treatments used
A (reference): Inert treatment
B: Acupuncture
C: Cognitive CBT
D: Education
E: Exercise
F: Manual therapy
G: NSAIDs
H: Paracetamol
I: Steroids
J: Usual care

# Figure 2. Funnel plot- disability

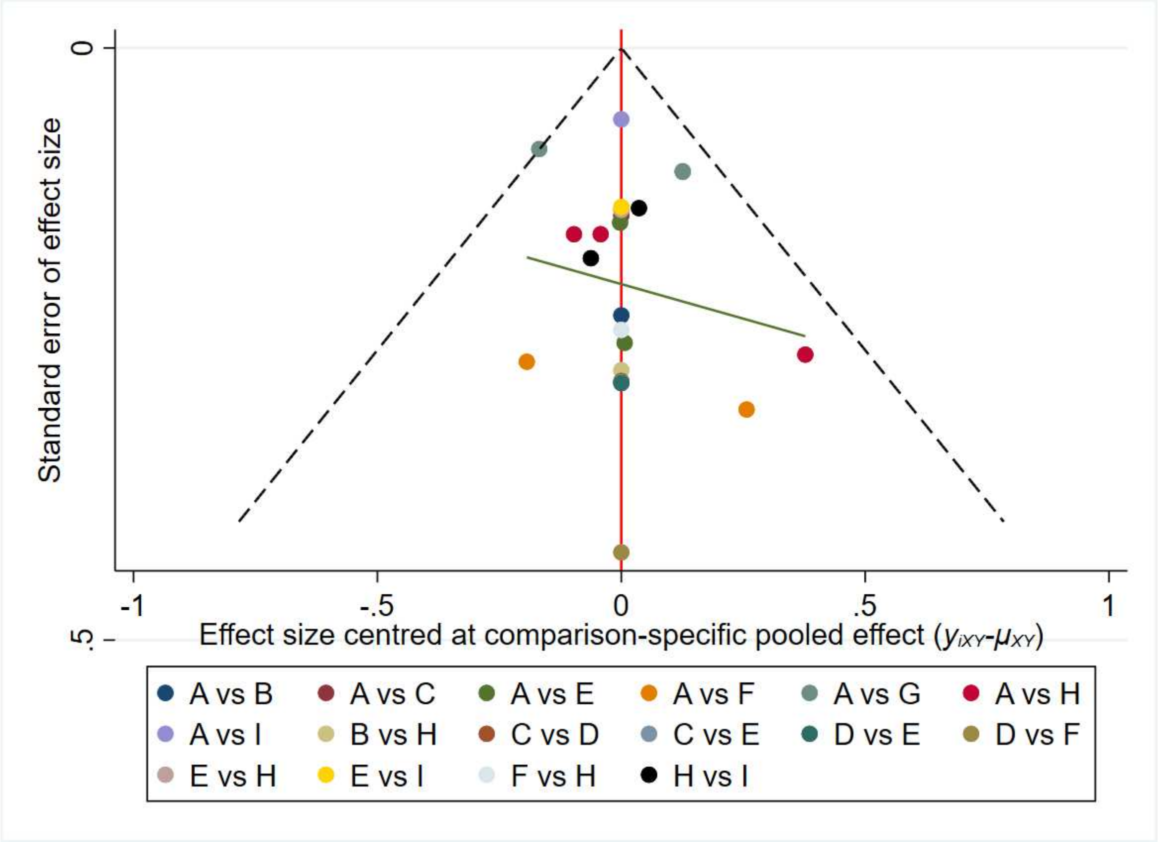


The red line represents the null hypothesis that the study-specific effect sizes do not differ from the respective comparison-specific pooled effect estimates. The green line is the regression line.

# Figure 2a. Disability Outcome 1 week

Legend:

Treatments used

| | |
|---|---|
| A (reference): | Inert treatment |
| B: | Acupuncture |
| C: | Education |
| D: | Exercise |
| E: | Heat wrap |
| F: | Manual therapy |
| G: | Muscle relaxant |
| H: | NSAIDs |
| I: | Paracetamol |

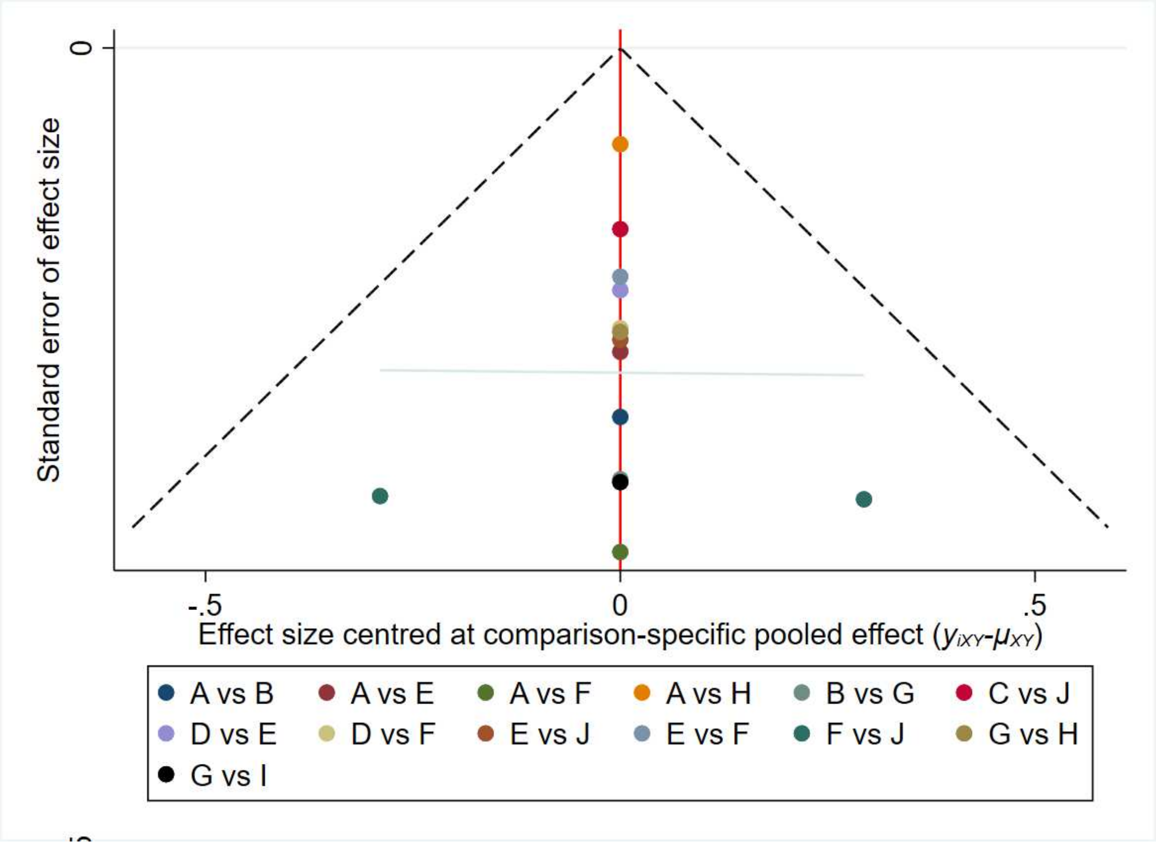


The red line represents the null hypothesis that the study-specific effect sizes do not differ from the respective comparison-specific pooled effect estimates. The gray line is the regression line.

## Figure 2b. Disability Outcome 1 month

Legend: Treatments used

A (reference): Inert treatment
B: Acupuncture
C: Cognitive CBT
D: Education
E: Exercise
F: Manual therapy
G: NSAIDs
H: Paracetamol
I: Steroids
J: Usual care

# Supplement O. Contribution matrix for the network on interventions

## Figure 1. Contribution matrix for the network on interventions - Pain

| Network meta-analysis estimates | Direct comparisons in the network | | | | | | | | | | | | | | | | |
|---|---|---|---|---|---|---|---|---|---|---|---|---|---|---|---|---|---|
| | AvsB | AvsC | AvsE | AvsF | AvsG | AvsH | AvsJ | AvsK | BvsH | CvsD | CvsE | DvsE | DvsF | FvsH | HvsI | HvsJ | IvsJ |
| **Mixed estimates** | | | | | | | | | | | | | | | | | |
| AvsB | 38.0 | 0.7 | 0.1 | 0.4 | | 11.7 | 9.7 | | 22.6 | 0.5 | 0.2 | 0.3 | 0.8 | 1.3 | 3.9 | 5.8 | 3.9 |
| AvsC | 0.4 | 72.4 | 4.4 | 0.5 | | 1.8 | 1.5 | | 0.4 | 4.3 | 4.3 | 0.1 | 4.2 | 3.7 | 0.6 | 0.9 | 0.6 |
| AvsE | 0.4 | 29.5 | 10.0 | 0.6 | | 2.2 | 1.8 | | 0.4 | 8.5 | 21.0 | 13.6 | 5.0 | 4.4 | 0.7 | 1.1 | 0.7 |
| AvsF | 2.7 | 6.0 | 1.1 | 3.8 | | 13.5 | 11.2 | | 2.7 | 4.3 | 1.7 | 2.8 | 7.1 | 27.4 | 4.5 | 6.7 | 4.5 |
| AvsG | | | | | 100.0 | | | | | | | | | | | | |
| AvsH | 5.4 | 1.6 | 0.3 | 1.0 | | 26.5 | 22.0 | | 5.4 | 1.1 | 0.4 | 0.7 | 1.9 | 2.9 | 8.8 | 13.2 | 8.8 |
| AvsJ | 1.0 | 0.3 | 0.1 | 0.2 | | 5.0 | 81.9 | | 1.0 | 0.2 | 0.1 | 0.1 | 0.4 | 0.5 | 2.6 | 3.9 | 2.6 |
| AvsK | | | | | | | | 100.0 | | | | | | | | | |
| BvsH | 27.7 | 0.8 | 0.2 | 0.5 | | 14.3 | 11.9 | | 24.3 | 0.6 | 0.2 | 0.4 | 1.0 | 1.5 | 4.7 | 7.1 | 4.7 |
| CvsD | 0.7 | 11.7 | 3.5 | 1.0 | | 3.6 | 3.0 | | 0.7 | 27.9 | 12.4 | 16.0 | 8.2 | 7.2 | 1.2 | 1.8 | 1.2 |
| CvsE | 0.3 | 13.7 | 10.0 | 0.4 | | 1.6 | 1.3 | | 0.3 | 14.4 | 31.0 | 18.1 | 3.7 | 3.2 | 0.5 | 0.8 | 0.5 |
| DvsE | 0.5 | 0.4 | 6.3 | 0.7 | | 2.5 | 2.1 | | 0.5 | 17.9 | 17.5 | 37.6 | 5.9 | 5.2 | 0.8 | 1.3 | 0.8 |
| DvsF | 1.6 | 15.8 | 2.9 | 2.2 | | 8.1 | 6.7 | | 1.6 | 11.4 | 4.4 | 7.3 | 12.2 | 16.4 | 2.7 | 4.0 | 2.7 |
| FvsH | 1.3 | 7.4 | 1.4 | 4.7 | | 6.6 | 5.5 | | 1.3 | 5.4 | 2.1 | 3.4 | 8.8 | 44.3 | 2.2 | 3.3 | 2.2 |
| HvsI | 1.8 | 0.5 | 0.1 | 0.3 | | 8.9 | 11.7 | | 1.8 | 0.4 | 0.1 | 0.2 | 0.6 | 1.0 | 48.8 | 6.0 | 17.7 |
| HvsJ | 4.3 | 1.3 | 0.2 | 0.8 | | 21.4 | 28.1 | | 4.3 | 0.9 | 0.4 | 0.6 | 1.5 | 2.3 | 9.7 | 14.5 | 9.7 |
| IvsJ | 2.7 | 0.8 | 0.1 | 0.5 | | 13.5 | 17.7 | | 2.7 | 0.6 | 0.2 | 0.4 | 1.0 | 1.5 | 26.9 | 9.2 | 22.1 |
| **Indirect estimates** | | | | | | | | | | | | | | | | | |
| AvsD | 0.7 | 26.8 | 4.9 | 1.0 | | 3.6 | 3.0 | | 0.7 | 19.4 | 7.5 | 12.4 | 8.4 | 7.4 | 1.2 | 1.8 | 1.2 |
| AvsI | 2.9 | 0.9 | 0.2 | 0.5 | | 14.5 | 24.5 | | 2.9 | 0.6 | 0.2 | 0.4 | 1.0 | 1.6 | 25.2 | 6.2 | 18.3 |
| BvsC | 23.1 | 32.7 | 2.0 | | | 6.3 | 5.2 | | 14.0 | 2.3 | 2.1 | 0.1 | 2.4 | 2.4 | 2.1 | 3.1 | 2.1 |
| BvsD | 17.4 | 18.8 | 3.4 | 0.5 | | 2.9 | 2.4 | | 11.2 | 13.6 | 5.2 | 8.7 | 6.4 | 5.9 | 1.0 | 1.5 | 1.0 |
| BvsE | 17.5 | 18.5 | 6.3 | 0.2 | | 4.1 | 3.4 | | 10.9 | 5.2 | 13.3 | 8.8 | 3.6 | 3.4 | 1.4 | 2.0 | 1.4 |
| BvsF | 19.4 | 5.1 | 0.9 | 3.2 | | 5.6 | 4.6 | | 15.5 | 3.7 | 1.4 | 2.3 | 6.0 | 25.8 | 1.9 | 2.8 | 1.9 |
| BvsG | 23.7 | 0.4 | 0.1 | 0.3 | 37.8 | 7.3 | 6.0 | | 14.1 | 0.3 | 0.1 | 0.2 | 0.5 | 0.8 | 2.4 | 3.6 | 2.4 |
| BvsI | 21.2 | 0.3 | 0.1 | 0.2 | | 5.3 | 15.3 | | 16.7 | 0.2 | 0.1 | 0.1 | 0.4 | 0.6 | 24.3 | 1.7 | 13.5 |
| BvsJ | 27.0 | 0.4 | 0.1 | 0.2 | | 6.0 | 33.6 | | 16.9 | 0.3 | 0.1 | 0.2 | 0.4 | 0.6 | 4.1 | 6.2 | 4.1 |
| BvsK | 23.7 | 0.4 | 0.1 | 0.3 | | 7.3 | 6.0 | 37.8 | 14.1 | 0.3 | 0.1 | 0.2 | 0.5 | 0.8 | 2.4 | 3.6 | 2.4 |
| CvsF | 2.1 | 22.9 | 0.8 | 2.9 | | 10.3 | 8.5 | | 2.1 | 5.2 | 3.0 | 2.2 | 7.4 | 20.9 | 3.4 | 5.1 | 3.4 |
| CvsG | 0.2 | 40.0 | 2.5 | 0.3 | 44.7 | 1.0 | 0.8 | | 0.2 | 2.4 | 2.4 | 0.1 | 2.3 | 2.0 | 0.3 | 0.5 | 0.3 |
| CvsH | 3.2 | 31.3 | 1.8 | 0.4 | | 16.1 | 13.4 | | 3.2 | 2.6 | 2.2 | 0.4 | 3.0 | 3.5 | 5.4 | 8.0 | 5.4 |
| CvsI | 1.9 | 26.2 | 1.5 | 0.2 | | 9.3 | 16.4 | | 1.9 | 2.0 | 1.8 | 0.2 | 2.2 | 2.4 | 17.6 | 3.9 | 12.4 |
| CvsJ | 0.3 | 39.6 | 2.4 | 0.2 | | 1.5 | 40.3 | | 0.3 | 2.5 | 2.4 | | 2.5 | 2.3 | 1.7 | 2.5 | 1.7 |
| CvsK | 0.2 | 40.0 | 2.5 | 0.3 | | 1.0 | 0.8 | 44.7 | 0.2 | 2.4 | 2.4 | 0.1 | 2.3 | 2.0 | 0.3 | 0.5 | 0.3 |
| DvsG | 0.5 | 19.1 | 3.5 | 0.7 | 28.6 | 2.6 | 2.2 | | 0.5 | 13.8 | 5.3 | 8.8 | 6.0 | 5.3 | 0.9 | 1.3 | 0.9 |
| DvsH | 2.2 | 18.5 | 3.4 | 0.2 | | 10.9 | 9.1 | | 2.2 | 13.4 | 5.2 | 8.5 | 7.0 | 6.8 | 3.6 | 5.4 | 3.6 |
| DvsI | 1.2 | 15.8 | 2.9 | 0.3 | | 5.9 | 11.9 | | 1.2 | 11.4 | 4.4 | 7.3 | 5.7 | 5.4 | 14.8 | 2.4 | 9.5 |
| DvsJ | 0.2 | 20.2 | 3.7 | 0.7 | | 1.0 | 25.8 | | 0.2 | 14.6 | 5.6 | 9.3 | 6.5 | 5.8 | 1.8 | 2.7 | 1.8 |
| DvsK | 0.5 | 19.1 | 3.5 | 0.7 | | 2.6 | 2.2 | 28.6 | 0.5 | 13.8 | 5.3 | 8.8 | 6.0 | 5.3 | 0.9 | 1.3 | 0.9 |
| EvsF | 1.7 | 14.0 | 5.4 | 2.3 | | 8.4 | 7.0 | | 1.7 | 2.2 | 11.8 | 10.4 | 8.3 | 17.1 | 2.8 | 4.2 | 2.8 |
| EvsG | 0.3 | 20.4 | 6.9 | 0.4 | 30.8 | 1.5 | 1.3 | | 0.3 | 5.9 | 14.5 | 9.4 | 3.5 | 3.1 | 0.5 | 0.8 | 0.5 |
| EvsH | 2.4 | 18.0 | 6.2 | 0.1 | | 11.9 | 9.9 | | 2.4 | 4.9 | 13.1 | 9.0 | 4.1 | 4.3 | 3.9 | 5.9 | 3.9 |
| EvsI | 1.4 | 15.5 | 5.3 | | | 6.9 | 12.7 | | 1.4 | 4.3 | 11.3 | 7.6 | 3.3 | 3.3 | 14.4 | 2.8 | 9.8 |
| EvsJ | | 21.0 | 7.1 | 0.4 | | 0.2 | 28.2 | | 0.1 | 6.0 | 15.0 | 9.8 | 3.7 | 3.4 | 1.5 | 2.2 | 1.5 |
| EvsK | 0.3 | 20.4 | 6.9 | 0.4 | | 1.5 | 1.3 | 30.8 | 0.3 | 5.9 | 14.5 | 9.4 | 3.5 | 3.1 | 0.5 | 0.8 | 0.5 |
| FvsG | 2.0 | 4.3 | 0.8 | 2.7 | 27.7 | 9.8 | 8.1 | | 2.0 | 3.1 | 1.2 | 2.0 | 5.1 | 19.8 | 3.2 | 4.9 | 3.2 |
| FvsI | 0.1 | 5.1 | 0.9 | 3.2 | | 0.7 | 10.1 | | 0.1 | 3.7 | 1.4 | 2.3 | 6.0 | 27.9 | 25.8 | 1.2 | 11.3 |
| FvsJ | 2.0 | 5.2 | 0.9 | 3.2 | | 10.0 | 21.3 | | 2.0 | 3.7 | 1.4 | 2.4 | 6.1 | 24.4 | 5.0 | 7.4 | 5.0 |
| FvsK | 2.0 | 4.3 | 0.8 | 2.7 | | 9.8 | 8.1 | 27.7 | 2.0 | 3.1 | 1.2 | 2.0 | 5.1 | 19.8 | 3.2 | 4.9 | 3.2 |
| GvsH | 3.4 | 1.0 | 0.2 | 0.6 | 36.2 | 16.9 | 14.1 | | 3.4 | 0.7 | 0.3 | 0.5 | 1.2 | 1.8 | 5.6 | 8.4 | 5.6 |
| GvsI | 2.0 | 0.6 | 0.1 | 0.4 | 30.3 | 10.1 | 17.1 | | 2.0 | 0.4 | 0.2 | 0.3 | 0.7 | 1.1 | 17.5 | 4.3 | 12.8 |
| GvsJ | 0.5 | 0.2 | | 0.1 | 46.9 | 2.7 | 43.4 | | 0.5 | 0.1 | | 0.1 | 0.2 | 0.3 | 1.4 | 2.1 | 1.4 |
| GvsK | | | | | 50.0 | | | 50.0 | | | | | | | | | |
| HvsK | 3.4 | 1.0 | 0.2 | 0.6 | | 16.9 | 14.1 | 36.2 | 3.4 | 0.7 | 0.3 | 0.5 | 1.2 | 1.8 | 5.6 | 8.4 | 5.6 |
| IvsK | 2.0 | 0.6 | 0.1 | 0.4 | | 10.1 | 17.1 | 30.3 | 2.0 | 0.4 | 0.2 | 0.3 | 0.7 | 1.1 | 17.5 | 4.3 | 12.8 |
| JvsK | 0.5 | 0.2 | | 0.1 | | 2.7 | 43.4 | 46.9 | 0.5 | 0.1 | | 0.1 | 0.2 | 0.3 | 1.4 | 2.1 | 1.4 |
| **Entire network** | 5.4 | 13.1 | 2.5 | 0.9 | 7.0 | 7.1 | 12.1 | 7.0 | 4.0 | 5.3 | 4.9 | 4.7 | 3.9 | 7.2 | 6.3 | 3.8 | 4.7 |
| **Included studies** | 1 | 1 | 1 | 2 | 4 | 3 | 1 | 1 | 1 | 1 | 1 | 1 | 1 | 1 | 2 | 2 | 1 |

# Figure 1a. Contribution matrix for the network on interventions Pain Outcome 1 week

Label: direct comparisons in the network are presented in the columns, and their contributions to the combined treatment effect are presented in the rows. The entries of the matrix are the percentage weights attributed to each direct comparison. The intervention labels are: A (reference): Inert treatment; B:Acupuncture; C: Education; D: Exercise; E: Heat wrap; F: Manual therapy; G: Muscle relaxant; H: NSAIDs; I: Opioid; J: Paracetamol; K: Physical therapy

**Direct comparisons in the network**

| Network meta-analysis estimates | AvsB | AvsE | AvsF | AvsH | BvsG | CvsJ | DvsE | EvsJ | FvsJ | GvsH | GvsI |
|---|---|---|---|---|---|---|---|---|---|---|---|
| Mixed estimates | | | | | | | | | | | |
| AvsB | 40.4 | | | 19.9 | 19.9 | | | | | 19.9 | |
| AvsE | | 72.8 | 9.1 | | | | | 9.1 | 9.1 | | |
| AvsF | | 18.5 | 44.6 | | | | | 18.5 | 18.5 | | |
| AvsH | 2.2 | | | 93.5 | 2.2 | | | | | 2.2 | |
| BvsG | 23.7 | | | 23.7 | 29.0 | | | | | 23.7 | |
| CvsJ | | | | | | 100.0 | | | | | |
| DvsE | | | | | | | 100.0 | | | | |
| EvsJ | | 8.9 | 8.9 | | | | | 73.4 | 8.9 | | |
| FvsJ | | 24.7 | 24.7 | | | | | 24.7 | 25.8 | | |
| GvsH | 14.2 | | | 14.2 | 14.2 | | | | | 57.5 | |
| GvsI | | | | | | | | | | | 100.0 |
| Indirect estimates | | | | | | | | | | | |
| AvsC | | 26.1 | 7.3 | | | 33.3 | | 26.1 | 7.3 | | |
| AvsD | | 40.0 | 5.0 | | | | 45.0 | 5.0 | 5.0 | | |
| AvsG | 11.0 | | | 39.0 | 11.0 | | | | | 39.0 | |
| AvsI | 7.3 | | | 26.0 | 7.3 | | | | | 26.0 | 33.3 |
| AvsJ | | 39.1 | 10.9 | | | | | 39.1 | 10.9 | | |
| BvsC | 14.4 | 16.8 | 4.7 | 7.1 | 7.1 | 21.5 | | 16.8 | 4.7 | 7.1 | |
| BvsD | 17.3 | 22.9 | 2.8 | 8.5 | 8.5 | | 25.8 | 2.8 | 2.8 | 8.5 | |
| BvsE | 23.3 | 30.9 | 3.8 | 11.4 | 11.5 | | | 3.8 | 3.8 | 11.4 | |
| BvsF | 20.7 | 9.0 | 21.8 | 10.2 | 10.2 | | | 9.0 | 9.0 | 10.2 | |
| BvsH | 32.4 | | | 32.4 | 17.6 | | | | | 17.6 | |
| BvsI | 15.5 | | | 15.5 | 19.0 | | | | | 15.5 | 34.5 |
| BvsJ | 18.3 | 21.4 | 6.0 | 9.0 | 9.0 | | | 21.4 | 6.0 | 9.0 | |
| CvsD | | 3.4 | 3.4 | | | 31.1 | 31.1 | 27.7 | 3.4 | | |
| CvsE | | 4.9 | 4.9 | | | 45.1 | | 40.3 | 4.9 | | |
| CvsF | | 16.4 | 16.4 | | | 33.6 | | 16.4 | 17.2 | | |
| CvsG | 4.4 | 15.6 | 4.4 | 15.6 | 4.4 | 20.0 | | 15.6 | 4.4 | 15.6 | |
| CvsH | 0.6 | 19.3 | 5.4 | 24.2 | 0.6 | 24.7 | | 19.3 | 5.4 | 0.6 | |
| CvsI | 3.7 | 13.0 | 3.6 | 13.0 | 3.7 | 16.7 | | 13.0 | 3.6 | 13.0 | 16.7 |
| DvsF | | 19.9 | 19.9 | | | | 33.3 | 13.4 | 13.4 | | |
| DvsG | 5.2 | 21.1 | 2.6 | 18.5 | 5.2 | | 23.7 | 2.6 | 2.6 | 18.5 | |
| DvsH | 0.7 | 27.2 | 3.4 | 29.9 | 0.7 | | 30.6 | 3.4 | 3.4 | 0.7 | |
| DvsI | 4.2 | 17.0 | 2.1 | 14.9 | 4.2 | | 19.2 | 2.1 | 2.1 | 14.9 | 19.2 |
| DvsJ | | 4.9 | 4.9 | | | | 45.1 | 40.3 | 4.9 | | |
| EvsF | | 29.8 | 29.8 | | | | | 20.2 | 20.2 | | |
| EvsG | 6.8 | 27.6 | 3.4 | 24.2 | 6.8 | | | 3.4 | 3.4 | 24.2 | |
| EvsH | 1.0 | 39.2 | 4.9 | 43.1 | 1.0 | | | 4.9 | 4.9 | 1.0 | |
| EvsI | 5.2 | 21.1 | 2.6 | 18.5 | 5.2 | | | 2.6 | 2.6 | 18.5 | 23.7 |
| FvsG | 6.1 | 8.2 | 19.7 | 21.7 | 6.1 | | | 8.2 | 8.2 | 21.7 | |
| FvsH | 0.9 | 11.1 | 26.9 | 37.1 | 0.9 | | | 11.1 | 11.1 | 0.9 | |
| FvsI | 4.8 | 6.4 | 15.4 | 17.0 | 4.8 | | | 6.4 | 6.4 | 17.0 | 21.8 |
| GvsJ | 5.5 | 19.5 | 5.5 | 19.5 | 5.5 | | | 19.5 | 5.5 | 19.5 | |
| HvsI | 8.2 | | | 8.3 | 8.2 | | | | | 33.5 | 41.7 |
| HvsJ | 0.7 | 25.7 | 7.2 | 32.1 | 0.7 | | | 25.7 | 7.2 | 0.7 | |
| IvsJ | 4.4 | 15.6 | 4.4 | 15.6 | 4.4 | | | 15.6 | 4.4 | 15.6 | 20.0 |
| **Entire network** | 6.8 | 16.1 | 7.3 | 14.9 | 5.1 | 7.0 | 7.0 | 12.2 | 5.4 | 11.1 | 7.0 |
| **Included studies** | 1 | 1 | 2 | 1 | 1 | 1 | 1 | 1 | 2 | 1 | 1 |

# Figure 1b. Contribution matrix for the network on interventions Pain Outcome 1 month

Label: direct comparisons in the network are presented in the columns, and their contributions to the combined treatment effect are presented in the rows. The entries of the matrix are the percentage weights attributed to each direct comparison. The intervention labels are: A (reference): Inert treatment; B: Acupuncture; C: Cognitive CBT; D: Education; E: Exercise; F: Manual therapy; G: NSAIDs; H: Paracetamol; I: Steroids; J:Usual care

| Network meta-analysis estimates | | Direct comparisons in the network: AvsC | BvsE | CvsD | DvsE |
|---|---|---|---|---|---|
| **Mixed estimates** | | | | | |
| | AvsC | 100.0 | | | |
| | BvsE | | 100.0 | | |
| | CvsD | | | 100.0 | |
| | DvsE | | | | 100.0 |
| **Indirect estimates** | | | | | |
| | AvsB | 25.0 | 25.0 | 25.0 | 25.0 |
| | AvsD | 50.0 | | 50.0 | |
| | AvsE | 33.3 | | 33.3 | 33.3 |
| | BvsC | | 33.3 | 33.3 | 33.3 |
| | BvsD | | 50.0 | | 50.0 |
| | CvsE | | | 50.0 | 50.0 |
| **Entire network** | | 20.0 | 20.0 | 30.0 | 30.0 |
| **Included studies** | | 1 | 2 | 1 | 1 |

## Figure 1c. Contribution matrix for the network on interventions Pain Outcome 12 months

Label: direct comparisons in the network are presented in the columns, and their contributions to the combined treatment effect are presented in the rows. The entries of the matrix are the percentage weights attributed to each direct comparison. The intervention labels are: A (reference): Inert treatment; B: Cognitive CBT; C: Education; D: Exercise; E: Usual care

**Direct comparisons in the network**

| Network meta-analysis estimates | | AvsC | BvsE | CvsD | DvsE |
|---|---|---|---|---|---|
| Mixed estimates | | | | | |
| | AvsC | 100.0 | | | |
| | BvsE | | 100.0 | | |
| | CvsD | | | 100.0 | |
| | DvsE | | | | 100.0 |
| Indirect estimates | | | | | |
| | AvsB | 25.0 | 25.0 | 25.0 | 25.0 |
| | AvsD | 50.0 | | 50.0 | |
| | AvsE | 33.3 | | 33.3 | 33.3 |
| | BvsC | | 33.3 | 33.3 | 33.3 |
| | BvsD | | 50.0 | | 50.0 |
| | CvsE | | | 50.0 | 50.0 |
| **Entire network** | | 20.0 | 20.0 | 30.0 | 30.0 |
| **Included studies** | | 1 | 2 | 1 | 1 |

# Figure 2. Contribution matrix for the network on interventions - Disability

## Figure 2a. Contribution matrix for the network on interventions Disability Outcome 12 months

Label: direct comparisons in the network are presented in the columns, and their contributions to the combined treatment effect are presented in the rows. The entries of the matrix are the percentage weights attributed to each direct comparison. The intervention labels are: The intervention labels are: A (reference): B: Cognitive CBT; C: Education; D: Exercise; E: Usual care

| Network meta-analysis estimates | | Direct comparisons in the network | | | | |
|---|---|---|---|---|---|---|
| | | AvsC | BvsD | BvsE | CvsD | DvsE |
| Mixed estimates | AvsC | 100.0 | | | | |
| | BvsD | | 46.2 | 26.9 | | 26.9 |
| | BvsE | | 25.6 | 48.8 | | 25.6 |
| | CvsD | | | | 100.0 | |
| | DvsE | | 22.3 | 22.3 | | 55.3 |
| Indirect estimates | AvsB | 29.7 | 18.7 | 10.9 | 29.7 | 10.9 |
| | AvsD | 50.0 | | | 50.0 | |
| | AvsE | 30.4 | 8.8 | 8.8 | 30.4 | 21.7 |
| | BvsC | | 26.7 | 15.6 | 42.2 | 15.6 |
| | CvsE | | 12.6 | 12.6 | 43.7 | 31.1 |
| **Entire network** | | 20.7 | 16.1 | 13.6 | 31.1 | 18.6 |
| **Included studies** | | 1 | 1 | 3 | 1 | 2 |



Gianola S, *et al. Br J Sports Med* 2021; 56:41–50. doi: 10.1136/bjsports-2020-103596

# Supplement P. GRADE for Pain Outcome

### Introduction

CINeMA[26] considers 6 domains: (i) within-study bias, (ii) reporting bias, (iii) indirectness, (iv) imprecision, (v) heterogeneity, and (vi) incoherence. Features include the percentage contribution matrix, relative treatment effects for each comparison, estimation of the heterogeneity variance, prediction intervals, and tests for the evaluation of the assumption of coherence. In evaluating imprecision, heterogeneity, and incoherence, we consider the impact of these components of variability in forming clinical decisions.

### Table of reasons for downgrading

We use the CINeMA software for GRADE assessment.[26 27] We downgrade network estimate according to the following criteria.

(1) Study limitations: We downgraded by one level when the contributions from low RoB comparisons were less than 25% and contributions from moderate or high RoB comparisons were 75% or greater.

(2) Imprecision: We considered a clinically meaningful threshold for SMD to be 0.5 [28] and downgraded the estimate if the SMD point estimate is 0 or more and the lower limit of its CrI is below 0.5; or if the SMD point estimate is less than 0 and the upper limit of its CrI is above 0.5.

(3) Inconsistency: We rated two concepts, heterogeneity and incoherence (inconsistency), in this domain.

For heterogeneity, we looked at the common tau and found that it is low compared to the expected value as reported in the literature,[29] so we did not downgrade any network estimate for heterogeneity. For inconsistency, we looked at the results of side splitting and we downgraded the comparisons with important inconsistency ($p<0.10$), where we have not downgraded for imprecision (we did not downgrade the same network estimate for both imprecision and inconsistency).

(4) Indirectness: We have assured transitivity in our network by limiting the included studies to acute and subacute population and to non-mixed treatments for NS-LBP. Thus, we did not downgrade for indirectness.

(5) Reporting bias: We cannot completely rule out the possibility that some studies are still missing. However, we assumed that publication bias was undetected.



Gianola S, *et al. Br J Sports Med* 2021; 56:41–50. doi: 10.1136/bjsports-2020-103596

# 1) Pain at 1 week

## 1) Summary of study limitations of the included studies

The colours in the circles indicate the percentage of low RoB studies [green], moderate RoB studies [yellow] and high RoB studies [red] involving each intervention. The colours of the line then indicate the average RoB assessment of each comparison based on the above information – low RoB comparison [green], moderate RoB comparison [yellow] and high RoB comparison [red].

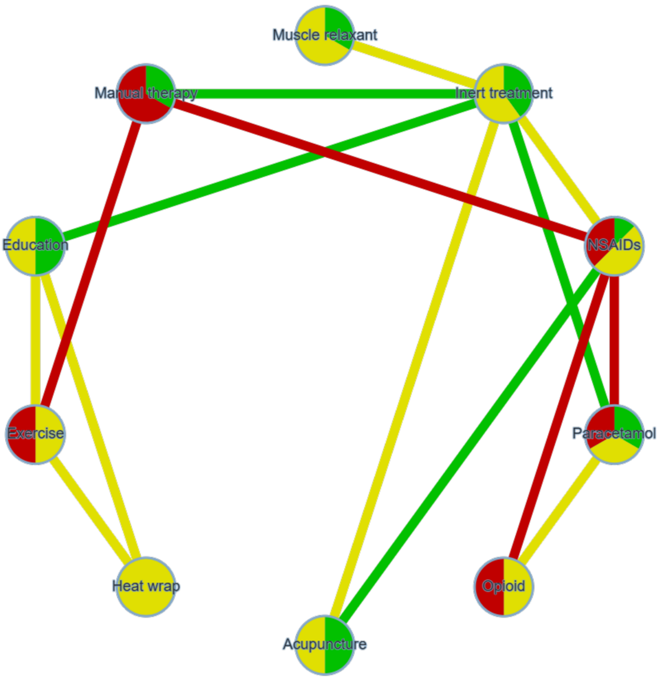


## 2) Contribution of low or moderate RoB comparisons to each network estimate

Based on the above assessment of RoB for each comparison and the contribution matrix detailing contribution of each direct comparison to all network estimates, the following bar graphs show the percentage of low or moderate RoB contributions for each network estimate. The judgements about study limitations in each direct comparison is shown at the beginning of the graph. Each bar corresponds to a NMA relative treatment effect and shows how much information comes from comparisons at moderate risk of bias [yellow].

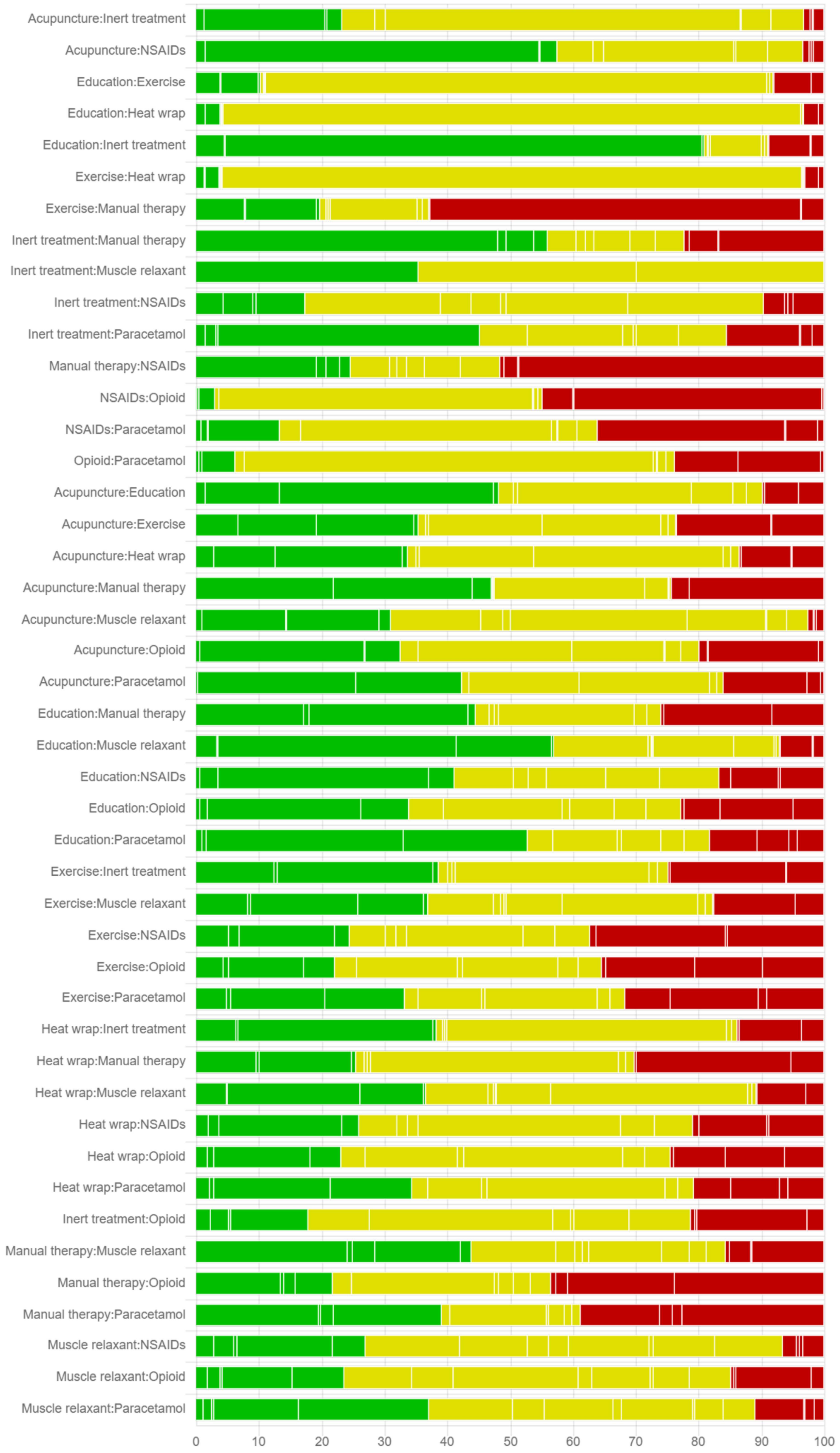
Acupuncture:Inert treatment
Acupuncture:NSAIDs
Education:Exercise
Education:Heat wrap
Education:Inert treatment
Exercise:Heat wrap
Exercise:Manual therapy
Inert treatment:Manual therapy
Inert treatment:Muscle relaxant
Inert treatment:NSAIDs
Inert treatment:Paracetamol
Manual therapy:NSAIDs
NSAIDs:Opioid
NSAIDs:Paracetamol
Opioid:Paracetamol
Acupuncture:Education
Acupuncture:Exercise
Acupuncture:Heat wrap
Acupuncture:Manual therapy
Acupuncture:Muscle relaxant
Acupuncture:Opioid
Acupuncture:Paracetamol
Education:Manual therapy
Education:Muscle relaxant
Education:NSAIDs
Education:Opioid
Education:Paracetamol
Exercise:Inert treatment
Exercise:Muscle relaxant
Exercise:NSAIDs
Exercise:Opioid
Exercise:Paracetamol
Heat wrap:Inert treatment
Heat wrap:Manual therapy
Heat wrap:Muscle relaxant
Heat wrap:NSAIDs
Heat wrap:Opioid
Heat wrap:Paracetamol
Inert treatment:Opioid
Manual therapy:Muscle relaxant
Manual therapy:Opioid
Manual therapy:Paracetamol
Muscle relaxant:NSAIDs
Muscle relaxant:Opioid
Muscle relaxant:Paracetamol
0
10
20
30
40
50
60
70
80
90
100

### 3) Summary grading of Evidence

| Comparison | Number of studies | Within-study bias | Reporting bias | Indirectness | Imprecision | Heterogeneity | Incoherence | Confidence rating |
|---|---|---|---|---|---|---|---|---|
| **Mixed evidence** | | | | | | | | |
| Acupuncture:Inert treatment | 1 | Some concerns | Undetected | No concerns | Major concerns | No concerns | No concerns | Very low |
| Acupuncture:NSAIDs | 1 | No concerns | Undetected | No concerns | Some concerns | Some concerns | No concerns | Moderate |
| Education:Exercise | 1 | Some concerns | Undetected | No concerns | No concerns | Some concerns | No concerns | Moderate |
| Education:Heat wrap | 1 | Some concerns | Undetected | No concerns | No concerns | Some concerns | No concerns | Moderate |
| Education:Inert treatment | 1 | No concerns | Undetected | No concerns | Major concerns | No concerns | No concerns | Low |
| Exercise:Heat wrap | 1 | Some concerns | Undetected | No concerns | Major concerns | No concerns | No concerns | Very low |
| Exercise:Manual therapy | 1 | Some concerns | Undetected | No concerns | Some concerns | Some concerns | No concerns | Low |
| Inert treatment:Manual therapy | 1 | Some concerns | Undetected | No concerns | Some concerns | Some concerns | No concerns | Low |
| Inert treatment:Muscle relaxant | 3 | Some concerns | Undetected | No concerns | No concerns | Some concerns | No concerns | Moderate |
| Inert treatment:NSAIDs | 3 | Some concerns | Undetected | No concerns | No concerns | Some concerns | No concerns | Moderate |
| Inert treatment:Paracetamol | 1 | Some concerns | Undetected | No concerns | Some concerns | Some concerns | No concerns | Low |
| Manual therapy:NSAIDs | 1 | Some concerns | Undetected | No concerns | Major concerns | No concerns | No concerns | Very low |
| NSAIDs:Opioid | 2 | Some concerns | Undetected | No concerns | Some concerns | Some concerns | No concerns | Low |
| NSAIDs:Paracetamol | 2 | Some concerns | Undetected | No concerns | Major concerns | No concerns | No concerns | Very low |

| | | | | | | | | |
|---|---|---|---|---|---|---|---|---|
| Opioid:Paracetamol | 1 | Some concerns | Undetected | No concerns | Some concerns | Some concerns | No concerns | Low |
| **Indirect evidence** | | | | | | | | |
| Acupuncture:Education | 0 | Some concerns | Undetected | No concerns | Major concerns | No concerns | No concerns | Very low |
| Acupuncture:Exercise | 0 | Some concerns | Undetected | No concerns | Some concerns | Some concerns | No concerns | Low |
| Acupuncture:Heat wrap | 0 | Some concerns | Undetected | No concerns | Some concerns | Some concerns | No concerns | Low |
| Acupuncture:Manual therapy | 0 | Some concerns | Undetected | No concerns | Some concerns | Some concerns | No concerns | Low |
| Acupuncture:Muscle relaxant | 0 | Some concerns | Undetected | No concerns | Some concerns | Some concerns | No concerns | Low |
| Acupuncture:Opioid | 0 | Some concerns | Undetected | No concerns | Some concerns | Some concerns | No concerns | Low |
| Acupuncture:Paracetamol | 0 | Some concerns | Undetected | No concerns | Major concerns | No concerns | No concerns | Very low |
| Education:Manual therapy | 0 | Some concerns | Undetected | No concerns | Some concerns | Some concerns | No concerns | Low |
| Education:Muscle relaxant | 0 | Some concerns | Undetected | No concerns | Major concerns | No concerns | No concerns | Very low |
| Education:NSAIDs | 0 | Some concerns | Undetected | No concerns | Major concerns | No concerns | No concerns | Very low |
| Education:Opioid | 0 | Some concerns | Undetected | No concerns | Major concerns | No concerns | No concerns | Very low |
| Education:Paracetamol | 0 | Some concerns | Undetected | No concerns | Major concerns | No concerns | No concerns | Very low |
| Exercise:Inert treatment | 0 | Some concerns | Undetected | No concerns | No concerns | Some concerns | No concerns | Moderate |
| Exercise:Muscle relaxant | 0 | Some concerns | Undetected | No concerns | Major concerns | No concerns | No concerns | Very low |
| Exercise:NSAIDs | 0 | Some concerns | Undetected | No concerns | Some concerns | Some concerns | No concerns | Low |
| Exercise:Opioid | 0 | Some concerns | Undetected | No concerns | Major concerns | No concerns | No concerns | Very low |

| | | | | | | | | |
|---|---|---|---|---|---|---|---|---|
| Exercise:Paracetamol | 0 | Some concerns | Undetected | No concerns | Some concerns | Some concerns | No concerns | Low |
| Heat wrap:Inert treatment | 0 | Some concerns | Undetected | No concerns | No concerns | Some concerns | No concerns | Moderate |
| Heat wrap:Manual therapy | 0 | Some concerns | Undetected | No concerns | Major concerns | No concerns | No concerns | Very low |
| Heat wrap:Muscle relaxant | 0 | Some concerns | Undetected | No concerns | Major concerns | No concerns | No concerns | Very low |
| Heat wrap:NSAIDs | 0 | Some concerns | Undetected | No concerns | Some concerns | Some concerns | No concerns | Low |
| Heat wrap:Opioid | 0 | Some concerns | Undetected | No concerns | Major concerns | No concerns | No concerns | Very low |
| Heat wrap:Paracetamol | 0 | Some concerns | Undetected | No concerns | Some concerns | Some concerns | No concerns | Low |
| Inert treatment:Opioid | 0 | Some concerns | Undetected | No concerns | No concerns | Some concerns | No concerns | Moderate |
| Manual therapy:Muscle relaxant | 0 | Some concerns | Undetected | No concerns | Major concerns | No concerns | No concerns | Very low |
| Manual therapy:Opioid | 0 | Some concerns | Undetected | No concerns | Major concerns | No concerns | No concerns | Very low |
| Manual therapy:Paracetamol | 0 | Some concerns | Undetected | No concerns | Major concerns | No concerns | No concerns | Very low |
| Muscle relaxant:NSAIDs | 0 | Some concerns | Undetected | No concerns | Major concerns | No concerns | No concerns | Very low |
| Muscle relaxant:Opioid | 0 | Some concerns | Undetected | No concerns | Major concerns | No concerns | No concerns | Very low |
| Muscle relaxant:Paracetamol | 0 | Some concerns | Undetected | No concerns | Major concerns | No concerns | No concerns | Very low |

## 2) Pain at 1 month

### 1) Summary of study limitations of the included studies

The colours in the circles indicate the percentage of low RoB studies [green], moderate RoB studies [yellow] and high RoB studies [red] involving each intervention. The colours of the line then indicate the average RoB assessment of each comparison based on the above information – low RoB comparison [green], moderate RoB comparison [yellow] and high RoB comparison [red].

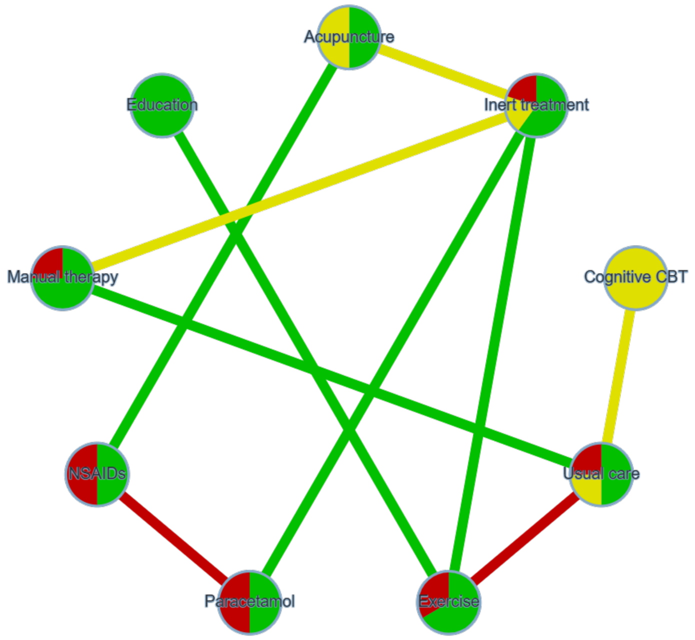


### 2) Contribution of low or moderate RoB comparisons to each network estimate

Based on the above assessment of RoB for each comparison and the contribution matrix detailing contribution of each direct comparison to all network estimates, the following bar graphs show the percentage of low or moderate RoB contributions for each network estimate. The judgements about study limitations in each direct comparison is shown at the beginning of the graph. Each bar corresponds to a NMA relative treatment effect and shows how much information comes from comparisons at moderate risk of bias [yellow].

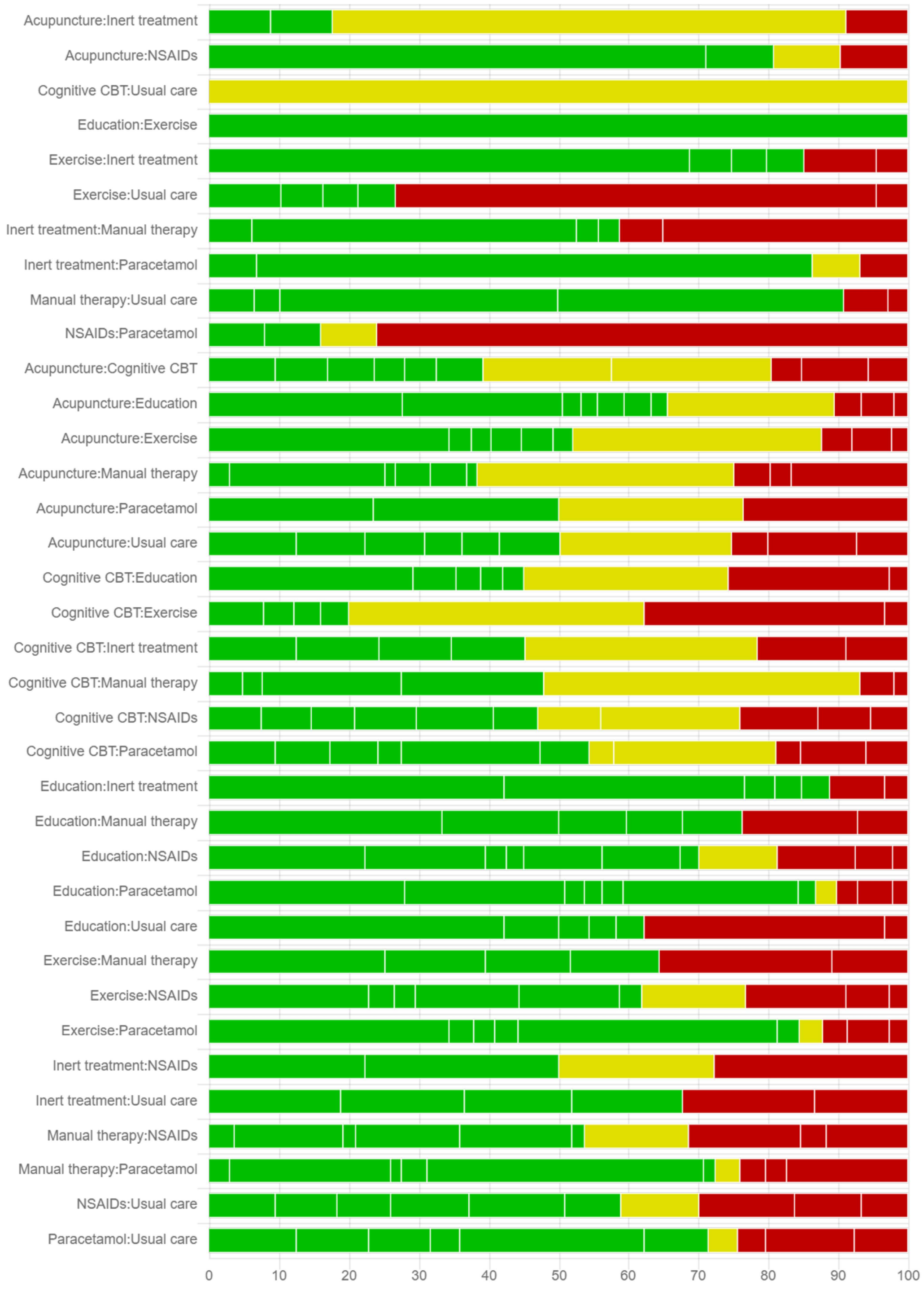
Acupuncture:Inert treatment
Acupuncture:NSAIDs
Cognitive CBT:Usual care
Education:Exercise
Exercise:Inert treatment
Exercise:Usual care
Inert treatment:Manual therapy
Inert treatment:Paracetamol
Manual therapy:Usual care
NSAIDs:Paracetamol
Acupuncture:Cognitive CBT
Acupuncture:Education
Acupuncture:Exercise
Acupuncture:Manual therapy
Acupuncture:Paracetamol
Acupuncture:Usual care
Cognitive CBT:Education
Cognitive CBT:Exercise
Cognitive CBT:Inert treatment
Cognitive CBT:Manual therapy
Cognitive CBT:NSAIDs
Cognitive CBT:Paracetamol
Education:Inert treatment
Education:Manual therapy
Education:NSAIDs
Education:Paracetamol
Education:Usual care
Exercise:Manual therapy
Exercise:NSAIDs
Exercise:Paracetamol
Inert treatment:NSAIDs
Inert treatment:Usual care
Manual therapy:NSAIDs
Manual therapy:Paracetamol
NSAIDs:Usual care
Paracetamol:Usual care
0
10
20
30
40
50
60
70
80
90
100

### 3) Summary grading of Evidence

| Comparison | Number of studies | Within-study bias | Reporting bias | Indirectness | Imprecision | Heterogeneity | Incoherence | Confidence rating |
|---|---|---|---|---|---|---|---|---|
| **Mixed evidence** | | | | | | | | |
| Acupuncture:Inert treatment | 1 | Some concerns | Undetected | No concerns | Some concerns | Some concerns | No concerns | Low |
| Acupuncture:NSAIDs | 1 | No concerns | Undetected | No concerns | Major concerns | No concerns | No concerns | Low |
| Cognitive CBT:Usual care | 1 | Some concerns | Undetected | No concerns | Major concerns | No concerns | No concerns | Very low |
| Education:Exercise | 1 | No concerns | Undetected | No concerns | Some concerns | Some concerns | No concerns | Moderate |
| Exercise:Inert treatment | 1 | No concerns | Undetected | No concerns | Major concerns | No concerns | No concerns | Low |
| Exercise:Usual care | 1 | Some concerns | Undetected | No concerns | Major concerns | No concerns | No concerns | Very low |
| Inert treatment:Manual therapy | 2 | Some concerns | Undetected | No concerns | No concerns | Some concerns | No concerns | Moderate |
| Inert treatment:Paracetamol | 1 | No concerns | Undetected | No concerns | Some concerns | Some concerns | No concerns | Moderate |
| Manual therapy:Usual care | 2 | No concerns | Undetected | No concerns | No concerns | Some concerns | No concerns | High |
| NSAIDs:Paracetamol | 1 | Major concerns | Undetected | No concerns | Major concerns | No concerns | No concerns | Very low |
| **Indirect evidence** | | | | | | | | |
| Acupuncture:Cognitive CBT | | Some concerns | Undetected | No concerns | Major concerns | No concerns | No concerns | Very low |
| Acupuncture:Education | | No concerns | Undetected | No concerns | Some concerns | Some concerns | No concerns | Moderate |
| Acupuncture:Exercise | | Some concerns | Undetected | No concerns | Major concerns | No concerns | No concerns | Very low |

| | | | | | | | |
|---|---|---|---|---|---|---|---|
| Acupuncture:Manual therapy | Some concerns | Undetected | No concerns | Some concerns | Some concerns | No concerns | Low |
| Acupuncture:Paracetamol | Some concerns | Undetected | No concerns | Major concerns | No concerns | No concerns | Very low |
| Acupuncture:Usual care | Some concerns | Undetected | No concerns | Major concerns | No concerns | No concerns | Very low |
| Cognitive CBT:Education | Some concerns | Undetected | No concerns | Some concerns | Some concerns | No concerns | Low |
| Cognitive CBT:Exercise | Some concerns | Undetected | No concerns | Major concerns | No concerns | No concerns | Very low |
| Cognitive CBT:Inert treatment | Some concerns | Undetected | No concerns | Major concerns | No concerns | No concerns | Very low |
| Cognitive CBT:Manual therapy | Some concerns | Undetected | No concerns | Some concerns | Some concerns | No concerns | Low |
| Cognitive CBT:NSAIDs | Some concerns | Undetected | No concerns | Major concerns | No concerns | No concerns | Very low |
| Cognitive CBT:Paracetamol | Some concerns | Undetected | No concerns | Major concerns | No concerns | No concerns | Very low |
| Education:Inert treatment | No concerns | Undetected | No concerns | Some concerns | Some concerns | No concerns | Moderate |
| Education:Manual therapy | No concerns | Undetected | No concerns | No concerns | Some concerns | No concerns | High |
| Education:NSAIDs | No concerns | Undetected | No concerns | Some concerns | Some concerns | No concerns | Moderate |
| Education:Paracetamol | No concerns | Undetected | No concerns | Some concerns | Some concerns | No concerns | Moderate |
| Education:Usual care | Some concerns | Undetected | No concerns | Some concerns | Some concerns | No concerns | Low |
| Exercise:Manual therapy | Some concerns | Undetected | No concerns | Some concerns | Some concerns | No concerns | Low |
| Exercise:NSAIDs | Some concerns | Undetected | No concerns | Major concerns | No concerns | No concerns | Very low |
| Exercise:Paracetamol | No concerns | Undetected | No concerns | Major concerns | No concerns | No concerns | Low |

| | | | | | | | |
|---|---|---|---|---|---|---|---|
| Inert treatment:NSAIDs | Some concerns | Undetected | No concerns | Some concerns | Some concerns | No concerns | Low |
| Inert treatment:Usual care | Some concerns | Undetected | No concerns | Major concerns | No concerns | No concerns | Very low |
| Manual therapy:NSAIDs | Some concerns | Undetected | No concerns | Major concerns | No concerns | No concerns | Very low |
| Manual therapy:Paracetamol | Some concerns | Undetected | No concerns | Some concerns | Some concerns | No concerns | Low |
| NSAIDs:Usual care | Some concerns | Undetected | No concerns | Major concerns | No concerns | No concerns | Very low |
| Paracetamol:Usual care | Some concerns | Undetected | No concerns | Major concerns | No concerns | No concerns | Very low |



Gianola S, *et al. Br J Sports Med* 2021; 56:41–50. doi: 10.1136/bjsports-2020-103596

## 3) Pain at 12 months

### 1) Summary of study limitations of the included studies

The colours in the circles indicate the percentage of low RoB studies [green], moderate RoB studies [yellow] and high RoB studies [red] involving each intervention. The colours of the line then indicate the average RoB assessment of each comparison based on the above information – low RoB comparison [green], moderate RoB comparison [yellow] and high RoB comparison [red].

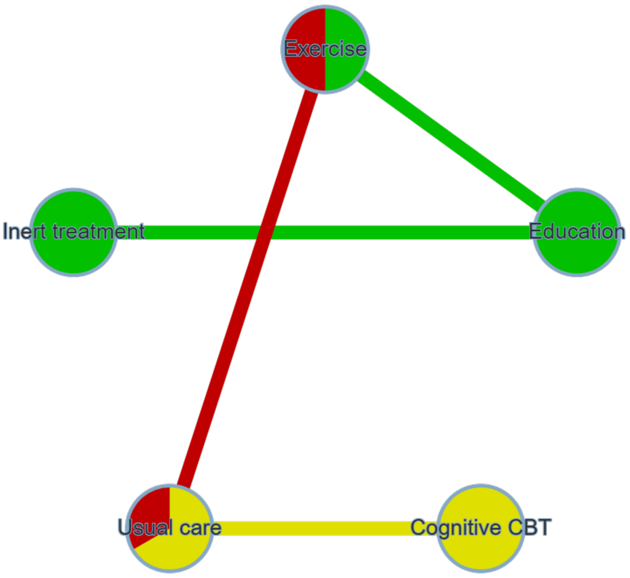


### 2) Contribution of low or moderate RoB comparisons to each network estimate

Based on the above assessment of RoB for each comparison and the contribution matrix detailing contribution of each direct comparison to all network estimates, the following bar graphs show the percentage of low or moderate RoB contributions for each network estimate. The judgements about study limitations in each direct comparison is shown at the beginning of the graph. Each bar corresponds to a NMA relative treatment effect and shows how much information comes from comparisons at moderate risk of bias [yellow].

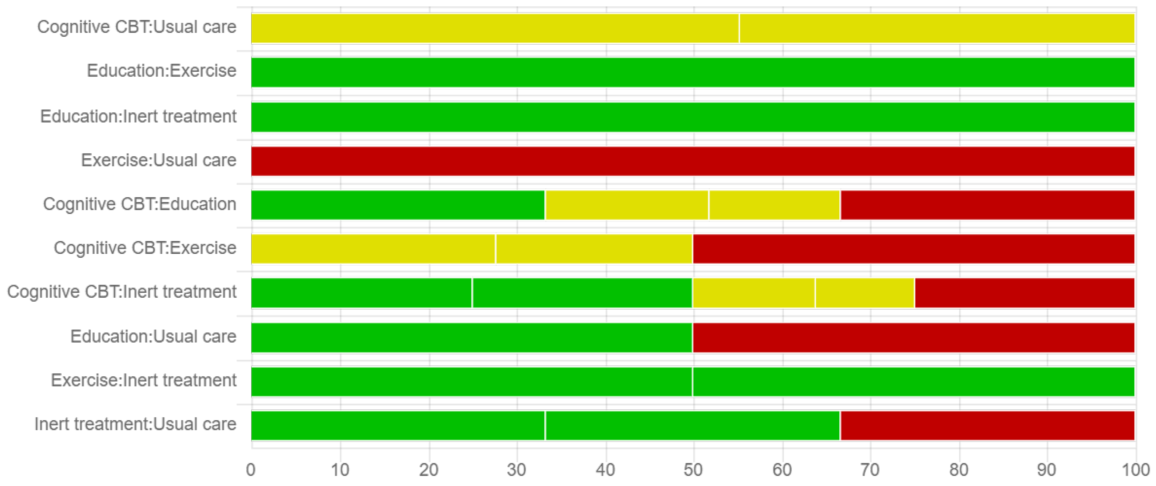


### 3) Summary grading of Evidence

| Comparison | Number of studies | Within-study bias | Reporting bias | Indirectness | Imprecision | Heterogeneity | Incoherence | Confidence rating |
|---|---|---|---|---|---|---|---|---|
| **Mixed treatment** | | | | | | | | |
| Cognitive CBT:Usual care | 2 | Some concerns | Undetected | No concerns | Some concerns | No concerns | No concerns | Low |
| Education:Exercise | 1 | No concerns | Undetected | No concerns | Some concerns | No concerns | No concerns | Moderate |
| Education:Inert treatment | 1 | No concerns | Undetected | No concerns | Some concerns | No concerns | No concerns | Moderate |
| Exercise:Usual care | 1 | Major concerns | Undetected | No concerns | Major concerns | No concerns | No concerns | Very low |
| **Indirect evidence** | | | | | | | | |
| Cognitive CBT:Education | - | Some concerns | Undetected | No concerns | Major concerns | No concerns | No concerns | Very low |

| | | | | | | | | |
|---|---|---|---|---|---|---|---|---|
| Cognitive CBT:Exercise | - | Major concerns | Undetected | No concerns | Major concerns | No concerns | No concerns | Very low |
| Cognitive CBT:Inert treatment | - | Some concerns | Undetected | No concerns | Major concerns | No concerns | No concerns | Very low |
| Education:Usual care | - | Some concerns | Undetected | No concerns | Major concerns | No concerns | No concerns | Very low |
| Exercise:Inert treatment | - | No concerns | Undetected | No concerns | Some concerns | No concerns | No concerns | Moderate |
| Inert treatment:Usual care | - | Some concerns | Undetected | No concerns | Major concerns | No concerns | No concerns | Very low |

## Supplement Q. GRADE for Disability Outcome

### Introduction

CINeMA considers 6 domains: (i) within-study bias, (ii) reporting bias, (iii) indirectness, (iv) imprecision, (v) heterogeneity, and (vi) incoherence. Features include the percentage contribution matrix, relative treatment effects for each comparison, estimation of the heterogeneity variance, prediction intervals, and tests for the evaluation of the assumption of coherence. In evaluating imprecision, heterogeneity, and incoherence, we consider the impact of these components of variability in forming clinical decisions.

### Table of reasons for downgrading

We use the CINeMA software for GRADE assessment.[26 27] We downgrade network estimate according to the following criteria.

(1) Study limitations: We downgraded by one level when the contributions from low RoB comparisons were less than 25% and contributions from moderate or high RoB comparisons were 75% or greater.

(2) Imprecision: We considered a clinically meaningful threshold for SMD to be 0.5 [28] and downgraded the estimate if the SMD point estimate is 0 or more and the lower limit of its CrI is below 0.5; or if the SMD point estimate is less than 0 and the upper limit of its CrI is above 0.5.

(3) Inconsistency: We rated two concepts, heterogeneity and incoherence (inconsistency), in this domain.

For heterogeneity, we looked at the common tau and found that it is low compared to the expected value as reported in the literature,[29] so we did not downgrade any network estimate for heterogeneity. For inconsistency, we looked at the results of side splitting and we downgraded the comparisons with important inconsistency (p<0.10), where we have not downgraded for imprecision (we did not downgrade the same network estimate for both imprecision and inconsistency).

(4) Indirectness: We have assured transitivity in our network by limiting the included studies to acute and subacute population and to non-mixed treatments for LBP. Thus, we did not downgrade for indirectness.

(5) Reporting bias: We cannot completely rule out the possibility that some studies are still missing. However, we assumed that publication bias was undetected.

# 1) Disability at 12 months

### 1- Summary of study limitations of the included studies

The colours in the circles indicate the percentage of low RoB studies [green], moderate RoB studies [yellow] and high RoB studies [red] involving each intervention. The colours of the line then indicate the average RoB assessment of each comparison based on the above information – low RoB comparison [green], moderate RoB comparison [yellow] and high RoB comparison [red].

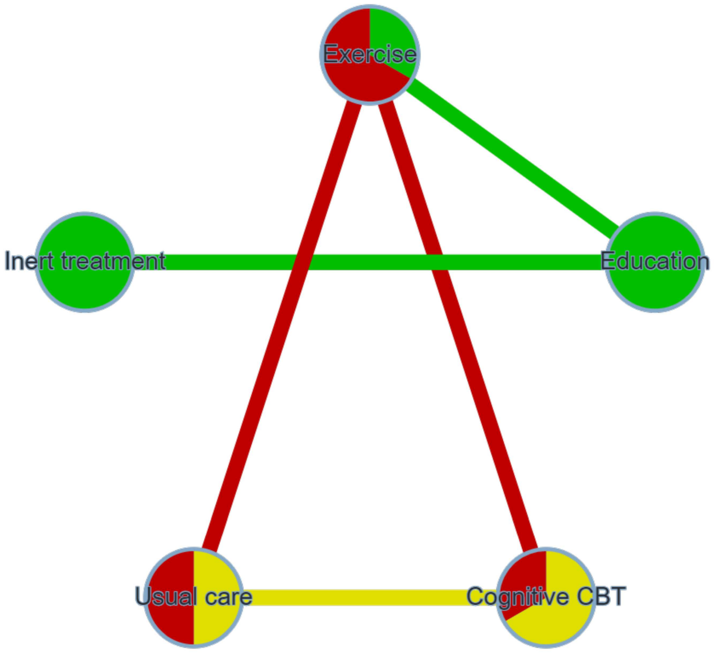

**2-Contribution of low or moderate RoB comparisons to each network estimate**

Based on the above assessment of RoB for each comparison and the contribution matrix detailing contribution of each direct comparison to all network estimates, the following bar graphs show the percentage of low or moderate RoB contributions for each network estimate. The judgements about study limitations in each direct comparison is shown at the beginning of the graph. Each bar corresponds to a NMA relative treatment effect and shows how much information comes from comparisons at low [green] and high risk of bias [high].

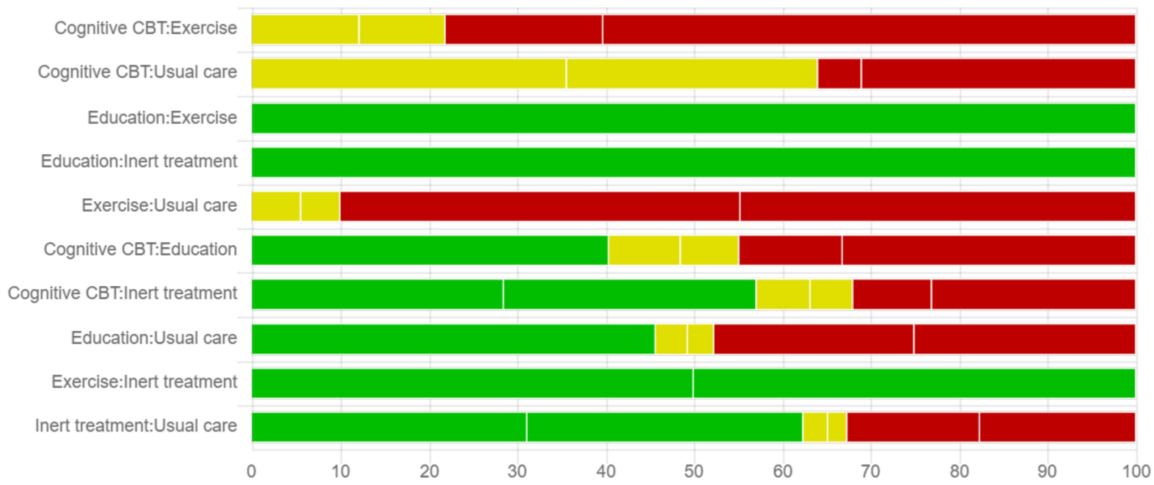

**3-Summary grading of Evidence**

| Comparison | Number of studies | Within-study bias | Reporting bias | Indirectness | Imprecision | Heterogeneity | Incoherence | Confidence rating |
|---|---|---|---|---|---|---|---|---|
| **Mixed evidence** | | | | | | | | |
| Cognitive CBT:Exercise | 1 | Major concerns | Undetected | No concerns | Some concerns | Some concerns | No concerns | Very low |
| Cognitive CBT:Usual care | 3 | Some concerns | Undetected | No concerns | Some concerns | Some concerns | No concerns | Low |
| Education:Exercise | 1 | No concerns | Undetected | No concerns | Some concerns | Some concerns | No concerns | Moderate |
| Education:Inert treatment | 1 | No concerns | Undetected | No concerns | Major concerns | No concerns | No concerns | Low |
| Exercise:Usual care | 2 | Major concerns | Undetected | No concerns | Some concerns | Some concerns | No concerns | Very low |
| **Indirect evidence** | | | | | | | | |
| Cognitive CBT:Education | - | Some concerns | Undetected | No concerns | Some concerns | Some concerns | No concerns | Low |
| Cognitive CBT:Inert treatment | - | Some concerns | Undetected | No concerns | Some concerns | Some concerns | No concerns | Low |
| Education:Usual care | - | Some concerns | Undetected | No concerns | Major concerns | No concerns | No concerns | Very low |
| Exercise:Inert treatment | - | No concerns | Undetected | No concerns | Some concerns | Some concerns | No concerns | Low |
| Inert treatment:Usual care | - | Some concerns | Undetected | No concerns | Major concerns | No concerns | No concerns | Very low |

## Supplement R. Data check

We checked the dataset for data extraction errors or "outlier effect sizes" having an influence on overall effects. We defined an "outlier effect sizes" of a study, visually inspecting forest plots of pairwise meta-analyses[30], when SMDs are greater than 1.5 [31 32] assuming 2 points of between population standard deviations across comparisons (resulting from the mean estimate of all final SD values in the control groups [33 34], see row dataset in OSF repository https://osf.io/sjr4y for 0-10 NRS scale). This calculation is coherent with literature where the MID between group difference is commonly set at 1 point (2 SD) on a NRS scale of 0-10 [35]. Coherently, in the Nice Guideline for Low Back Pain and Sciatica[36] the panel considered clinical important an improvement of 10% as a measure of clinical benefit e.g. 1 point decrease on a 0-10 scale for pain intensity [35].

# Supplement S. References


1. Hoffmann TC, Glasziou PP, Boutron I, et al. Better reporting of interventions: template for intervention description and replication (TIDieR) checklist and guide. *BMJ* 2014;348:g1687. doi: 10.1136/bmj.g1687 [published Online First: 2014/03/13]
2. Gianola S, Castellini G, Andreano A, et al. Effectiveness of treatments for acute and sub-acute mechanical non-specific low back pain: protocol for a systematic review and network meta-analysis. *Systematic reviews* 2019;8(1):196. doi: 10.1186/s13643-019-1116-3
3. Chou R, Deyo R, Friedly J, et al. Systemic Pharmacologic Therapies for Low Back Pain: A Systematic Review for an American College of Physicians Clinical Practice Guideline. *Ann Intern Med* 2017;166(7):480-92. doi: 10.7326/m16-2458 [published Online First: 2017/02/14]
4. Abdel Shaheed C, Maher CG, Williams KA, et al. Efficacy and tolerability of muscle relaxants for low back pain: Systematic review and meta-analysis. *European journal of pain* 2017;21(2):228-37. doi: 10.1002/ejp.907
5. van der Gaag WH, Roelofs PD, Enthoven WT, et al. Non-steroidal anti-inflammatory drugs for acute low back pain. *Cochrane Database Syst Rev* 2020;4:CD013581. doi: 10.1002/14651858.CD013581 [published Online First: 2020/04/16]
6. Sanger N, Bhatt M, Singhal N, et al. Adverse Outcomes Associated with Prescription Opioids for Acute Low Back Pain: A Systematic Review and Meta-Analysis. *Pain Physician* 2019;22(2):119-38. [published Online First: 2019/03/30]
7. Abdel Shaheed C, Maher CG, Williams KA, et al. Efficacy, Tolerability, and Dose-Dependent Effects of Opioid Analgesics for Low Back Pain: A Systematic Review and Meta-analysis. *JAMA Intern Med* 2016;176(7):958-68. doi: 10.1001/jamainternmed.2016.1251 [published Online First: 2016/05/24]
8. Saragiotto BT, Machado GC, Ferreira ML, et al. Paracetamol for low back pain. *Cochrane Database Syst Rev* 2016;2016(6):Cd012230. doi: 10.1002/14651858.Cd012230 [published Online First: 2016/06/09]
9. Shamliyan TA, Staal JB, Goldmann D, et al. Epidural steroid injections for radicular lumbosacral pain: a systematic review. *Phys Med Rehabil Clin N Am* 2014;25(2):471-89.e1-50. doi: 10.1016/j.pmr.2014.02.001 [published Online First: 2014/05/03]
10. Lee JH, Kim DH, Kim DH, et al. Comparison of Clinical Efficacy of Epidural Injection With or Without Steroid in Lumbosacral Disc Herniation: A Systematic Review and Meta-analysis. *Pain Physician* 2018;21(5):449-68. [published Online First: 2018/10/05]
11. Marin TJ, Van Eerd D, Irvin E, et al. Multidisciplinary biopsychosocial rehabilitation for subacute low back pain. *Cochrane Database Syst Rev* 2017;6(6):Cd002193. doi: 10.1002/14651858.CD002193.pub2 [published Online First: 2017/06/29]
12. Zahari Z, Ishak A, Justine M. The effectiveness of patient education in improving pain, disability and quality of life among older people with low back pain: A systematic review. *J Back Musculoskelet Rehabil* 2020;33(2):245-54. doi: 10.3233/bmr-181305 [published Online First: 2019/07/30]
13. Engers A, Jellema P, Wensing M, et al. Individual patient education for low back pain. *Cochrane Database Syst Rev* 2008;2008(1):Cd004057. doi: 10.1002/14651858.CD004057.pub3 [published Online First: 2008/02/07]
14. de Zoete RM, Armfield NR, McAuley JH, et al. Comparative effectiveness of physical exercise interventions for chronic non-specific neck pain: a systematic review with network meta-analysis of 40 randomised controlled trials. *Br J Sports Med* 2020 doi: 10.1136/bjsports-2020-102664 [published Online First: 2020/11/04]
15. Owen PJ, Miller CT, Mundell NL, et al. Which specific modes of exercise training are most effective for treating low back pain? Network meta-analysis. *Br J Sports Med* 2020;54(21):1279-87. doi: 10.1136/bjsports-2019-100886 [published Online First: 2019/11/02]
16. Kamonseki DH, Christenson P, Rezvanifar SC, et al. Effects of manual therapy on fear avoidance, kinesiophobia and pain catastrophizing in individuals with chronic musculoskeletal pain: Systematic review and meta-analysis. *Musculoskelet Sci Pract* 2020;51:102311. doi: 10.1016/j.msksp.2020.102311 [published Online First: 2020/12/11]

17. de Luca KE, Fang SH, Ong J, et al. The Effectiveness and Safety of Manual Therapy on Pain and Disability in Older Persons With Chronic Low Back Pain: A Systematic Review. *J Manipulative Physiol Ther* 2017;40(7):527-34. doi: 10.1016/j.jmpt.2017.06.008 [published Online First: 2017/10/29]
18. Thompson BT, Schoenfeld D. Usual care as the control group in clinical trials of nonpharmacologic interventions. *Proc Am Thorac Soc* 2007;4(7):577-82. doi: 10.1513/pats.200706-072JK [published Online First: 2007/09/20]
19. Kamper SJ, Logan G, Copsey B, et al. What is usual care for low back pain? A systematic review of health care provided to patients with low back pain in family practice and emergency departments. *Pain* 2020;161(4):694-702. doi: 10.1097/j.pain.0000000000001751 [published Online First: 2019/11/19]
20. Leucht S, Leucht C, Huhn M, et al. Sixty Years of Placebo-Controlled Antipsychotic Drug Trials in Acute Schizophrenia: Systematic Review, Bayesian Meta-Analysis, and Meta-Regression of Efficacy Predictors. *The American journal of psychiatry* 2017;174(10):927-42. doi: 10.1176/appi.ajp.2017.16121358
21. Diseases GBD, Injuries C. Global burden of 369 diseases and injuries in 204 countries and territories, 1990-2019: a systematic analysis for the Global Burden of Disease Study 2019. *Lancet* 2020;396(10258):1204-22. doi: 10.1016/S0140-6736(20)30925-9 [published Online First: 2020/10/19]
22. Casser HR, Seddigh S, Rauschmann M. Acute Lumbar Back Pain. *Dtsch Arztebl Int* 2016;113(13):223-34. doi: 10.3238/arztebl.2016.0223 [published Online First: 2016/04/28]
23. Higgins J, Green S. Cochrane Handbook for Systematic Reviews of Interventions Version 5.1.0 [updated March 2011]. Chichester, UK: Wiley and Sons, 2011.

2011.
24. Dias S, Welton NJ, Sutton AJ, et al. Evidence synthesis for decision making 4: inconsistency in networks of evidence based on randomized controlled trials. *Med Decis Making* 2013;33(5):641-56. doi: 10.1177/0272989X12455847 [published Online First: 2013/06/28]
25. Palmer SC, Mavridis D, Nicolucci A, et al. Comparison of Clinical Outcomes and Adverse Events Associated With Glucose-Lowering Drugs in Patients With Type 2 Diabetes: A Meta-analysis. *JAMA* 2016;316(3):313-24. doi: 10.1001/jama.2016.9400 [published Online First: 2016/07/21]
26. Nikolakopoulou A, Higgins JPT, Papakonstantinou T, et al. CINeMA: An approach for assessing confidence in the results of a network meta-analysis. *PLoS medicine* 2020;17(4):e1003082. doi: 10.1371/journal.pmed.1003082 [published Online First: 2020/04/04]
27. Papakonstantinou T, Nikolakopoulou A, Higgins J, et al. CINeMA: Software for semiautomated assessment of the confidence in the results of network meta-analysis *Campbell Systematic Reviews* 2020;16(e1080)
28. Guyatt GH, Oxman AD, Kunz R, et al. GRADE guidelines 6. Rating the quality of evidence--imprecision. *Journal of clinical epidemiology* 2011;64(12):1283-93. doi: 10.1016/j.jclinepi.2011.01.012
29. Turner RM, Davey J, Clarke MJ, et al. Predicting the extent of heterogeneity in meta-analysis, using empirical data from the Cochrane Database of Systematic Reviews. *International journal of epidemiology* 2012;41(3):818-27. doi: 10.1093/ije/dys041
30. Schoretsanitis G, de Filippis R, Ntogka M, et al. Matrix Metalloproteinase 9 Blood Alterations in Patients With Schizophrenia Spectrum Disorders: A Systematic Review and Meta-Analysis. *Schizophr Bull* 2021 doi: 10.1093/schbul/sbab001 [published Online First: 2021/01/26]
31. Faraone SV. Interpreting estimates of treatment effects: implications for managed care. *P t* 2008;33(12):700-11. [published Online First: 2009/09/15]
32. Sawilowsky, S (2009). "New effect size rules of thumb". Journal of Modern Applied Statistical Methods. 8 (2): 467–474. doi:10.22237/jmasm/1257035100.
33. WHO handbook https://www.who.int/hiv/topics/mtct/grade_handbook.pdf.
34. Higgins JPT, Deeks JJ, Altman DG (editors). Chapter 16: Special topics in statistics. In: Higgins JPT, Green S (editors), Cochrane Handbook for Systematic Reviews of Interventions Version 5.1.0 (updated March 2011). The Cochrane Collaboration, 2011. Available from www.handbook.cochrane.org.

35. Bagg MK, Hubscher M, Rabey M, et al. The RESOLVE Trial for people with chronic low back pain: protocol for a randomised clinical trial. *J Physiother* 2017;63(1):47-48. doi: 10.1016/j.jphys.2016.11.001 [published Online First: 2016/12/13]
36. Low back pain and sciatica in over 16s: assessment and management. NICE guideline [NG59]Published date: 30 November 2016 Last updated: 11 December 2020. https://www.nice.org.uk/guidance/ng59/evidence.
