# Supplementary material for: Effectiveness of treatments for acute and subacute mechanical non-specific low back pain: a systematic review with network meta-analysis
Source: Br J Sports Med. 2021 Apr 13;56(1):41–50. doi: 10.1136/bjsports-2020-103596 (PMC8685632; doi:10.1136/bjsports-2020-103596)
Supplement: Supplementary data [file bjsports-2020-103596supp002.pdf]

Multiple choice questions (MCQs)

1. Balancing benefits and harms, which is best strategy for the management of acute and subacute NS-LBP:

    A. pharmacological interventions
    B. non-pharmacological interventions
    C. bed rest
    D. surgery

2. Paracetamol can be recommended as a treatment choice for acute and subacute NS-LBP?

    A. yes, prescription of low dosage (500 mg/die)
    B. yes, prescription of higher dosage (4000 mg/die)
    C. yes, any dosage
    D. no, it is not superior to inert treatment

3. Among pharmacological interventions, which is best efficacious?

    A. Muscle relaxants
    B. NSAIDS
    C. opioids
    D. paracetamol

4. In which treatments mild and moderate adverse events are often present?
    A. manual therapy, heat wrap
    B. opioids, NSAIDS, steroids
    C. paracetamol
    D. muscle relaxant drugs

5. How was the overall certainty of the evidence for pain and disability outcomes in management of acute and subacute NS-LBP?

    A. the range of overall certainty of the evidence was high
    B. the range of overall certainty of the evidence was moderate
    C. the range of overall certainty of the evidence varied
    D. the overall certainty of the evidence was not assessed
